# Diet Quality Scores and Prediction of All-Cause, Cardiovascular and Cancer Mortality in a Pan-European Cohort Study

**DOI:** 10.1371/journal.pone.0159025

**Published:** 2016-07-13

**Authors:** Camille Lassale, Marc J. Gunter, Dora Romaguera, Linda M. Peelen, Yvonne T. Van der Schouw, Joline W. J. Beulens, Heinz Freisling, David C. Muller, Pietro Ferrari, Inge Huybrechts, Guy Fagherazzi, Marie-Christine Boutron-Ruault, Aurélie Affret, Kim Overvad, Christina C. Dahm, Anja Olsen, Nina Roswall, Konstantinos K. Tsilidis, Verena A. Katzke, Tilman Kühn, Brian Buijsse, José-Ramón Quirós, Emilio Sánchez-Cantalejo, Nerea Etxezarreta, José María Huerta, Aurelio Barricarte, Catalina Bonet, Kay-Tee Khaw, Timothy J. Key, Antonia Trichopoulou, Christina Bamia, Pagona Lagiou, Domenico Palli, Claudia Agnoli, Rosario Tumino, Francesca Fasanelli, Salvatore Panico, H. Bas Bueno-de-Mesquita, Jolanda M. A. Boer, Emily Sonestedt, Lena Maria Nilsson, Frida Renström, Elisabete Weiderpass, Guri Skeie, Eiliv Lund, Karel G. M. Moons, Elio Riboli, Ioanna Tzoulaki

**Affiliations:** 1 Department of Epidemiology and Biostatistics, School of Public Health, Imperial College London, United Kingdom; 2 Instituto de Investigación Sanitaria de Palma (IdISPa), Palma de Mallorca, Spain; 3 CIBER Fisiopatología de la Obesidad y Nutrición (CIBEROBN), Instituto de Salud Carlos III, Madrid, Spain; 4 Department of Epidemiology, Julius Center for Health Sciences and Primary Care, University Medical Center Utrecht, Utrecht, Netherlands; 5 International Agency for Research on Cancer, Lyon, France; 6 Institut National de la Santé et de la Recherche Médicale, Center for Research in Epidemiology and Population, Health, U1018, Team 9, Villejuif, France; 7 Institut Gustave Roussy, Villejuif, France; 8 Paris South University, Unité Mixte de Recherche 1018, Villejuif, France; 9 Section for Epidemiology, Department of Public Health, Aarhus University, Aarhus, Denmark; 10 Danish Cancer Society, Institute of Cancer Epidemiology, Copenhagen, Denmark; 11 German Cancer Research Center (DKFZ), Division of Cancer Epidemiology, Heidelberg, Germany; 12 Deutsches Institut für Ernährungsforschung Potsdam-Rehbrücke German Institute of Human Nutrition, Potsdam, Germany; 13 Department of Hygiene and Epidemiology, University of Ioannina School of Medicine, Ioannina, Greece; 14 Public Health Directorate, Asturias, Oviedo, Spain; 15 Andalusian School of Public Health, Granada, Spain; 16 Public Health Direction and Biodonostia Basque Regional Health Department, San Sebastian, Spain; 17 Department of Epidemiology, Murcia Regional Health Council, Murcia, Spain; 18 Navarre Public Health Institute, Pamplona, Spain; 19 Unit of Nutrition, Environment and Cancer, Catalan Institute of Oncology, Barcelona, Spain; 20 University of Cambridge School of Clinical Medicine, Clinical Gerontology Unit, Cambridge, United Kingdom; 21 Cancer Epidemiology Unit, Nuffield Department of Clinical Medicine, University of Oxford, United Kingdom; 22 Department of Hygiene, Epidemiology and Medical Statistics, University of Athens Medical School, Athens, Greece; 23 Hellenic Health Foundation, Athens, Greece; 24 Molecular and Nutritional Epidemiology Unit, Cancer Research and Prevention Institute—ISPO, Florence, Italy; 25 Epidemiology and Prevention Unit, Department of Preventive and Predictive Medicine, Foundation of the Carlo Besta Neurological Institute, Milan, Italy; 26 Cancer Registry and Histopathology Unit, “Civic MP Arezzo” Hospital, Ragusa, Italy; 27 Human Genetics Foundation, Turin, Italy; 28 Department of Clinical and Experimental Medicine, Federico II University, Naples, Italy; 29 Center for Nutrition, Prevention and Health Services, National Institute for Public Health and the Environment, Bilthoven, Netherlands; 30 Department of Clinical Sciences in Malmö, Lund University, Lund, Sweden; 31 Department of Public Health and Clinical Medicine, Nutritional Research, Umeå University, Umeå, Sweden; 32 Department of Community Medicine, Faculty of Health Sciences, University of Tromsø, The Arctic University of Norway, Tromsø, Norway; 33 Department of Research, Cancer Registry of Norway, Oslo, Norway; 34 Department of Medical Epidemiology and Biostatistics, Karolinska Institutet, Stockholm, Sweden; 35 Genetic Epidemiology Group, Folkhälsan Research Center, Helsinki, Finland; Tufts University, UNITED STATES

## Abstract

Scores of overall diet quality have received increasing attention in relation to disease aetiology; however, their value in risk prediction has been little examined. The objective was to assess and compare the association and predictive performance of 10 diet quality scores on 10-year risk of all-cause, CVD and cancer mortality in 451,256 healthy participants to the European Prospective Investigation into Cancer and Nutrition, followed-up for a median of 12.8y. All dietary scores studied showed significant inverse associations with all outcomes. The range of HRs (95% CI) in the top vs. lowest quartile of dietary scores in a composite model including non-invasive factors (age, sex, smoking, body mass index, education, physical activity and study centre) was 0.75 (0.72–0.79) to 0.88 (0.84–0.92) for all-cause, 0.76 (0.69–0.83) to 0.84 (0.76–0.92) for CVD and 0.78 (0.73–0.83) to 0.91 (0.85–0.97) for cancer mortality. Models with dietary scores alone showed low discrimination, but composite models also including age, sex and other non-invasive factors showed good discrimination and calibration, which varied little between different diet scores examined. Mean C-statistic of full models was 0.73, 0.80 and 0.71 for all-cause, CVD and cancer mortality. Dietary scores have poor predictive performance for 10-year mortality risk when used in isolation but display good predictive ability in combination with other non-invasive common risk factors.

## Introduction

Poor diet is a leading risk factor for all-cause mortality and mortality due to major non-communicable diseases including cardiovascular disease (CVD) and several cancers [1,2]. As foods are not consumed in isolation, scores of overall diet quality have received increased attention in disease prevention, compared to their single dietary components [3]. Numerous *a priori* diet quality scores have been developed in the medical literature ranging from regional/national dietary patterns, such as the Mediterranean diet [4], to indices based on national/international guidelines such as those from the World Health Organization (WHO) [5]. These scores have been thoroughly studied as etiological risk factors for all-cause or cause-specific mortality [6–13]. However, their potential value in risk prediction has been little examined [14,15].

Dietary scores could be useful for risk communication and targeted preventive lifestyle or pharmacological interventions. For example, risk prediction models including diet quality scores can be calculated non-invasively (i.e. do not require blood draw) and independently (self-assessment) which may enable better, earlier and continuous risk assessment as well as motivate adherence to lifestyle recommendations [16]. We used data from the large European Prospective Investigation into Cancer and Nutrition (EPIC) cohort study of men and women from 10 European countries to assess a comprehensive set of dietary scores in relation to all-cause and cause-specific (CVD and cancer) mortality. Our aim was to assess and compare the association and predictive performance of 10 diet quality scores on 10-year mortality risk, either alone or in combination with other non-invasively assessed predictors. A secondary objective was to examine the variability in their predictive performance between different countries.

## Methods

### Study population

EPIC is an on-going multicenter prospective cohort study investigating the role of diet, lifestyle, genetic and environmental factors on the risk of cancer and other chronic diseases. A detailed description of the methods employed has previously been published [17,18]. Briefly, 521,448 participants aged 25–70 years were recruited between 1992 and 2000, from 23 study centers in 10 European countries: Denmark, France, Germany, Greece, Italy, the Netherlands, Norway, Spain, Sweden, and the United Kingdom. Participants with previous cancer (n = 23,243), CVD (n = 7,007 myocardial infarction, n = 8,335 angina and n = 4,156 stroke) or diabetes (n = 13,844) diagnosis were excluded from this analysis. All study participants provided written informed consent. Ethical approval for the EPIC study was obtained from the review boards of the International Agency for Research on Cancer and local participating centres: National Committee on Health Research Ethics (Denmark); Comité de Protection des Personnes (France); Ethics Committee of the Heidelberg University Medical School (Germany); Ethikkommission der Landesärztekammer Brandenburg Cottbus (Germany); University of Athens Medical School (Greece) Comitato Etico Indipendente, Fondazione IRCCS Istituto Nazionale dei Tumori (Italy); Human Genetics Foundation Torino Ethics Committee (Italy); Medical Ethical Committee (METC) of the University Medical Center Utrecht (the Netherlands); Regional Ethical Committee for Northern Norway and the Norwegian Data Inspectorate (Norway); Comité de Ética de Investigación Clínica (Spain); Ethics Committee of Lund University (Sweden); Umea Regional Ethical Review Board (Sweden); Norwich District Ethics Committee (UK); Scotland A Research Ethics Committee (UK); and the Imperial College Research Ethics Committee (UK). Details on recruitment of participants, sample selection and dietary data collection can be found in the supporting information.

### Diet, lifestyle, and anthropometric data

Lifestyle questionnaires were used to obtain information on education, smoking habits, alcohol consumption, physical activity and breastfeeding. Data on occupational, recreational, and household PA during the past year either were obtained through a standardized questionnaire. The Cambridge Index of PA was derived by combining occupational with recreational activity level and is summarized into 4 groups: active, moderately active, moderately inactive, and inactive [19]. Anthropometric measures (body weight and height) were measured at physical examination (except in France, Norway and Oxford UK, self-reported). Body mass index (BMI) was defined as weight divided by squared height (kg/m^2^). Usual diet over the previous 12 months was assessed at study baseline using validated country/center specific dietary questionnaires [17,18], allowing the calculation of food group and individual nutrient intakes (derived from the EPIC nutrient database [20]). Food group classification in EPIC has been extensively described elsewhere [21]. A dietary calibration study was conducted on a random subsample of 36,308 participants who completed a standardized 24h dietary recall, hence dietary data across centers were scaled by using an additive calibration [22].

### Computation of diet quality scores

A total of ten scores were examined; two of them included a combination of dietary and lifestyle variables, while the remaining scores had dietary information only. A large number of diet quality scores exist in the literature [3,23,24]. We based our selection on existing reviews [24,25] and selected scores which have been widely examined, were developed for international comparisons, included only non-invasive data, are recommended by leading guidelines and if different versions existed, in their most recent version. Scores for which two components or more were unavailable in the study population were excluded e.g. alternate Healthy Eating Index (aHEI)[26], that requires data on sodium and trans fat, both unavailable in EPIC. The ten selected scores reflect different concepts and can be classified in three broad categories: 1) scores based on general nutritional guidelines: Diet Quality Index International (DQI-I) [27], Healthy Eating Index 2010 (HEI-2010) [28], WHO Healthy Diet Indicator (WHO HDI) [5] and Healthy Lifestyle Index (HLI) [29]; 2) scores which measure adherence to disease-specific dietary and lifestyle guidelines: WCRF/AICR guideline score for the prevention of cancer [13], the Dietary Approaches to Stop Hypertension (DASH) [30]; 3) scores that measure adherence to a regional diet, namely the Mediterranean diet through conceptually different scores, the Mediterranean Diet Score (MDS) [4], the relative Med diet score (rMED)[31] and the Mediterranean Style Dietary Pattern Score (MSDPS) [32], and a score that includes the healthy components of a Nordic diet, the Healthy Nordic Food Index (HNFI) [33]. Two scores also included lifestyle factors in addition to dietary information: HLI includes BMI, physical activity and smoking and WCRF/AICR score includes BMI, physical activity, and breastfeeding.

Computation and the scoring system for each score are summarized in Table 1 and the list of dietary items composing the different scores is provided in S1 Table. When one component of the score could not be computed due to data availability (sodium intake), we calculated a modified version of the score without the sodium component (total maximum score equals total score minus maximum score for the sodium component).

**Table 1:** Description of the scoring system of the 10 dietary (and lifestyle) scores.

Index	Type	Scoring	Cut-offs^a^	Ease of calculation
**Healthy Eating Index HEI-2010** Based on Dietary Guidelines for Americans 2010 [28]	Diet (Food and nutrients)	**12 components. Score range: 0–100 points**^b^ **Adequacy**: total fruit, whole fruit, total vegetables, greens and beans, whole grains, dairy, total protein foods, seafood and plant proteins, fatty acids balance **Moderation:** Refined grains, sodium, empty calories	Predefined	**Moderate** Available SAS macro^c^ Need to convert food group intake from grams to cup or ounce equivalents
**Diet Quality Index–International** DQI-I[27]	Diet (Food and nutrients)	**17 components. Score range: 0–100 points**^b^ **Variety**: overall food group variety (0–15 pts); within-group variety for protein source (0–5 pts) **Adequacy**: vegetables, fruits, cereals, fiber, protein, Fe, Ca, vitamin C (0–5 pts each). **Moderation**: total fat, saturated fat, cholesterol, sodium, empty-energy foods (0–6 pts) **Overall balance**: macronutrient ratio (carbohydrate:protein:fat, 0–6 pts); fatty acid ratio (PUFA:MUFA:SFA, 0–4 pts)	Predefined	**Complex** Definition of empty-energy foods requires complex calculation. Requires extensive nutrient information.
**WHO Healthy Diet Indicator** WHO HDI [5]	Diet (Food and nutrients)	**7 components. Score range: 0–7 points** SFA, PUFA, cholesterol, protein, dietary fiber; fruit and vegetables, free sugars	Predefined	**Simple**
**Healthy Lifestyle Index** HLI [29]	Diet (Food and nutrients) and lifestyle	**Healthy diet: 7 components. Score range: 0–63 points** Cereal fiber, folate, ratio PUFA:SFA, fatty fish (as a marker for omega-3 fatty acids), margarine (as a marker for trans-fats), glycaemic load, fruits and vegetables. **Center-specific deciles**, scored from 0 to 9 (inverse for trans-fat and glycaemic load), 0 = least healthy consumption **Health index: 5 components. Score range: 0–20** (4 points each) never smoking, no alcohol, intense physical activity, low BMI, healthy diet score (quintiles, the highest scores 4, the lowest 0)	Population dependent	**Moderate** Requires folate and glycaemic load and lifestyle indicators: smoking, BMI, physical activity and alcohol.
**Cancer WCRF guideline score** [13]	Diet (Food) and lifestyle	**7 components (women) or 6 components (men). Score range: 0–7 points (women), 0–6 (men)** Selected recommendations: weight management, physical activity, foods and drinks that promote weight gain (energy density and sugary drink intake), plant foods, animal foods, alcoholic drinks, breastfeeding (women only)	Predefined	**Moderate** Requires data on breastfeeding, BMI and physical activity.
**Hypertension** Dietary Approaches to Stop Hypertension **DASH** diet score[30]	Diet (Food)	**8 components. Score range 8–40 points (5 points each)**^b^ Negative components: sweet beverages, meat, sodium Beneficial components: Fruit, vegetables, legumes and nuts, wholegrain, low fat dairy. Sex-specific quintiles, scoring from 1 to 5 in each category.	Population dependent	**Simple** Rank intake into quintiles Requires detail on wholegrain and low fat dairy.
Mediterranean Diet Score **MDS**[4]	Diet (Food and nutrients)	**9 components. Score range: 0–9 points 5 beneficial** components 1 point if above sex-specific median, 0 if below: fruit, vegetable, legumes, grains, fish **2 detrimental** components 1 point if below median, 0 if above: meat, dairy products **1** component on **fat**: Mono-unsaturated Fatty Acids / Saturated Fatty Acids ratio **1** component on **ethanol**: 1 point if within a range of intake (10-50g/day for men, 5-25g/d for women)	Population dependent	**Simple** Rank intake according to the median
Relative Mediterranean score **rMED**[31]	Diet (Food and nutrients)	**9 components. Score range: 0–18 points** Same 5 beneficial components as MDS but points 0, 1, 2 attributed to tertiles 1, 2 and 3, inverse quotation for the 2 detrimental components Component on fat: olive oil intake 0 point for non-consumption, 1 point below median, 2 points above	Population dependent	**Simple** Rank intake into tertiles Requires data on olive oil.
Mediterranean Style Dietary Pattern Score **MSDPS**[32]	Diet (Food)	**13 components. Score range: up to 100 points** (10 points each) Whole-grain, cereals, fruits, vegetables, dairy, wine, fish, poultry, olives-legumes-nuts, potatoes, eggs, sweets, meats, olive oil. Scoring depends on number of servings recommended per day or week (e.g. consume 60% of recommended serving, score = 6). If consumption is above recommended number of servings, points are deducted accordingly. Negative points are possible.	Predefined	**Complex** Requires to classify “Mediterranean” and “non-mediterranean” food and their contribution to the overall diet.
Healthy Nordic Food Index **HNFI** [33]	Diet (Food)	**6 components**. **Score range**: **0–6 points** 1 point if above sex-specific median, 0 if below: fish and shellfish, cabbage, whole grain bread, apples and pears, root vegetables	Population dependent	**Simple** Rank intake according to the median Requires data on sub groups of vegetables (cabbage and root) and wholegrain.

^a^ Refers to the thresholds used to allocate points for each component.

^b^ Sodium data was not available. HEI-2010 score ranged between 0 and 95; DQI-I score ranged between 0 and 94 points; DASH score ranged between 7–35 points

^c^ http://appliedresearch.cancer.gov/hei/tools.html#calculating

### Outcomes

All-cause mortality risk was the primary outcome of this analysis and CVD and cancer mortality the secondary outcomes. We also performed sensitivity analysis looking at cancers strongly associated with obesity (including esophagus, liver, pancreas, colorectal, breast, endometrial, kidney, prostate and gall bladder cancer mortality) as their association with diet might be stronger compared to the overall cancer mortality group [34]. Data on causes of deaths were coded according to the International Classification of Diseases, 10^th^ Revision (ICD-10) [35]. Due to differences across participating centers in time to reporting the causes of deaths, follow-up length was truncated at the date when 80% of causes were known. Causal mortality information was available for 82% of all recorded deaths. The following causes of death were investigated in the present study: cancer (ICD-10: C00-D48) and circulatory diseases (I00-I99).

### Statistical analyses

We truncated follow-up at 10 years and derived Cox proportional hazard regression models, using time of follow-up as the primary time metric, allowing the estimation of hazard ratios (HRs) and 95% confidence intervals (95% CIs) for the risk of death at 10 years. Exit time was date of death or the last date at which follow-up was considered complete in each center (censoring), whichever came first. All scores were standardized (separately for men and women in each study center), to allow comparison of HRs, interpreted as a mortality ratio associated with the increase of 1 SD of the score. We also categorized individuals according to sex- and center–specific quartiles for each score to assess the shape of the association between diet scores and outcome (all-cause, CVD and cancer mortality).

In a first step, for each score we created a model that only included the standardized score and age, stratified by study center and sex (Model 1). In a second step, we added lifestyle indicators: BMI (continuous), smoking status (never, former, current smoker), physical activity level (inactive, moderately inactive, moderately active, active) and educational level (primary, technical/professional, secondary, longer education) in the model (Model 2). For the WCRF/AICR score, we only added educational level and smoking (because physical activity and BMI are included as components of the score) as predictors; similarly analyses with HLI were only adjusted for education (BMI, physical activity and smoking are components of the index).

Performance of the models in predicting risk of all-cause death at 10 years of follow-up was evaluated by their discrimination (whether the model can distinguish between individuals who did and did not have the mortality event) and calibration (to what extent the predicted probabilities agree with the reported risk) measures. Discrimination was assessed by the Harrell’s C-statistic, similar to the area under the receiver operator characteristic curve, AUC, adapted to survival analysis [36]. This indicator ranges from 0.5 (no discrimination) to 1 (perfect discrimination). Calibration was assessed graphically by plotting the observed risk per decile of predicted risk. A perfect calibration would be seen if the curve falls along the identity line. The predicted-to-observed risk ratios were also calculated.

To investigate heterogeneity between different countries we performed sensitivity analyses by fitting our Cox regression models in each country. We estimated the summary effect with random-effect meta-analyses and heterogeneity via the I^2^ metric [37]. We also used random effects meta-analyses to summarize the discrimination of the models across countries. Stratified analyses by sex and age categories (<50 and ≥50 years old) were carried out as sensitivity analyses.

All analyses were conducted using SAS (Cary, NC), version 9.3, R version 3.0.1 and Stata MP version 13.1.

## Results

The analysis sample consisted of 451,256 participants of the EPIC cohort, aged 50.8 (± 9.8) years at baseline, with a median follow-up of 12.8 years (Table 2). After 10 years of follow-up, 15,200 fatal events had occurred, of which 3,761 were due to cardiovascular causes and 7,475 due to cancer.

**Table 2:** Characteristics of the study population included, n = 451,256 participants from the European Prospective Investigation into Cancer and Nutrition (EPIC).

	Males	n = 130,370	Females	n = 320,886	Total n = 451,256	
	Mean^a^	SD^a^	Mean^a^	SD^a^	Mean^a^	SD^a^
**Age**	51.5	10.0	50.6	9.7	50.8	9.8
**Length of follow-up (Median, interquartile range)**	12.6	11.4–14.0	12.9	11.4–14.6	12.8	11.4–14.5
**Energy intake (kcal)**	2427.9	662.3	1935.4	539.5	2077.7	619.3
**BMI**	26.4	3.6	24.9	4.3	25.3	4.2
	**n**	**%**	**n**	**%**	**n**	**%**
**BMI category**						
Underweight (<18.5)	534	0.4	6,483	2.0	7,017	1.6
Normal weight (18.5–24.9)	47,513	36.4	184,186	57.4	231,699	51.4
Overweight (25–29.9)	63,429	48.7	92,162	28.7	155,591	34.5
Obese (≥30)	18,894	14.5	38,055	11.9	56,949	12.6
**Smoking status**						
Never	44,208	33.9	177,886	55.4	222,094	49.2
Former	45,635	35.0	72,251	22.5	117,886	26.1
Current	38,677	29.7	63,206	19.7	101,883	22.6
**Education**						
None/Primary	42,526	33.5	88,685	28.7	131,211	30.1
Technical/professional	31,590	24.9	69,451	22.5	101,041	23.2
Secondary	17,534	13.8	76,728	24.9	94,262	21.6
Longer education	35,403	27.9	73,845	23.9	109,248	25.1
**Physical activity (Cambridge index)**						
Inactive	22,788	17.5	66,330	20.7	89,118	19.8
Moderately inactive	39,947	30.6	101,731	31.7	141,678	31.4
Moderately active	32,240	24.7	69,401	21.6	101,641	22.5
Active	32,451	24.9	45,267	14.1	77,718	17.2
**Number of fatal events**						
All-cause	11,118	8.5	13,876	4.3	24,994	5.5
Cancer	4,312	3.3	5,936	1.9	10,248	2.3
**Obesity-related cancer**	1,668	1.3	2,250	0.7	3,938	0.9
CVD	2,782	2.1	2,336	0.7	5,118	1.1
**Number of fatal events after 10 years**						
All-cause	7,181	5.5	8,019	2.5	15,200	3.4
Cancer	3,221	2.5	4,254	1.33	7,475	1.7
Obesity-related cancer	1,226	0.9	1,645	0.5	2,871	0.6
CVD	2,133	1.6	1,628	0.5	3,761	0.8

^a^ Values are means and standard deviation, unless otherwise stated

### Associations with all-cause, CVD and cancer mortality

Comparing quartiles for each score, there was a statistically significant inverse linear trend in all-cause mortality for all scores. In Model 1, the effect size of the association with mortality varied little between scores and consistently showed a lower risk of all-cause mortality per SD increase, with HR ranging from 0.83 (DQI-I) to 0.92 (WHO HDI) for diet only scores (Table 3). The two scores, HLI and WCRF/AICR, which included other lifestyle factors beyond diet displayed stronger associations. Further inclusion of lifestyle variables (Model 2, Table 4), resulted in attenuation of HRs which still remained highly significant (ranging from 0.89 [rMED] to 0.95 [WHO HDI]).

**Table 3:** Hazard ratios (Model 1)^a^ for 10-year mortality risk by quartile (Q) of diet quality score and for a 1SD increase of diet quality among 451,256 participants of the EPIC study.

	HR	HR (95% CI)	HR (95% CI)	HR (95% CI)	HR (95% CI)	P trend^c^
All-cause mortality	Q1	Q2	Q3	Q4	Continuous^b^	
MDS	1 (ref)	0.86 (0.82–0.90)	0.79 (0.75–0.82)	0.70 (0.67–0.74)	0.87 (0.86–0.89)	<0.0001
rMED	1 (ref)	0.82 (0.79–0.86)	0.73 (0.70–0.77)	0.67 (0.64–0.70)	0.85 (0.83–0.86)	<0.0001
MSDPS	1 (ref)	0.88 (0.84–0.92)	0.81 (0.78–0.85)	0.72 (0.69–0.76)	0.88 (0.86–0.89)	<0.0001
DQI-I	1 (ref)	0.82 (0.78–0.85)	0.71 (0.68–0.74)	0.63 (0.60–0.66)	0.83 (0.82–0.85)	<0.0001
HNFI	1 (ref)	0.89 (0.85–0.93)	0.79 (0.76–0.83)	0.73 (0.70–0.76)	0.89 (0.87–0.90)	<0.0001
HEI 2010	1 (ref)	0.83 (0.80–0.87)	0.76 (0.73–0.79)	0.71 (0.68–0.75)	0.86 (0.85–0.88)	<0.0001
WHO HDI	1 (ref)	0.91 (0.87–0.95)	0.83 (0.79–0.87)	0.80 (0.76–0.84)	0.92 (0.90–0.93)	<0.0001
DASH	1 (ref)	0.85 (0.81–0.88)	0.76 (0.73–0.79)	0.69 (0.66–0.72)	0.86 (0.85–0.87)	<0.0001
HLI–diet	1 (ref)	0.86 (0.82–0.90)	0.78 (0.75–0.82)	0.72 (0.69–0.75)	0.88 (0.87–0.90)	<0.0001
HLI—total^d^	1 (ref)	0.75 (0.71–0.78)	0.65 (0.62–0.68)	0.54 (0.52–0.57)	0.78 (0.77–0.80)	<0.0001
WCRF^e^	1 (ref)	0.83 (0.80–0.87)	0.72 (0.69–0.76)	0.63 (0.60–0.66)	0.83 (0.81–0.84)	<0.0001
**CVD mortality**						
MDS	1 (ref)	0.77 (0.71–0.84)	0.73 (0.67–0.80)	0.69 (0.63–0.76)	0.87 (0.84–0.89)	0.03
rMED	1 (ref)	0.82 (0.76–0.89)	0.72 (0.65–0.78)	0.65 (0.59–0.71)	0.83 (0.81–0.86)	<0.0001
MSDPS	1 (ref)	0.88 (0.81–0.96)	0.83 (0.76–0.90)	0.72 (0.66–0.79)	0.88 (0.85–0.91)	<0.0001
DQI-I	1 (ref)	0.83 (0.76–0.90)	0.69 (0.63–0.76)	0.62 (0.56–0.68)	0.83 (0.81–0.86)	<0.0001
HNFI	1 (ref)	0.87 (0.79–0.95)	0.79 (0.72–0.87)	0.71 (0.65–0.78)	0.88 (0.85–0.91)	<0.0001
HEI 2010	1 (ref)	0.84 (0.77–0.91)	0.77 (0.71–0.84)	0.72 (0.65–0.78)	0.87 (0.85–0.90)	0.001
WHO HDI	1 (ref)	0.92 (0.84–1.00)	0.81 (0.74–0.89)	0.73 (0.67–0.81)	0.89 (0.86–0.91)	<0.0001
DASH	1 (ref)	0.80 (0.74–0.88)	0.71 (0.65–0.77)	0.63 (0.57–0.69)	0.83 (0.80–0.86)	<0.0001
HLI–diet	1 (ref)	0.88 (0.81–0.96)	0.76 (0.70–0.83)	0.67 (0.61–0.73)	0.85 (0.83–0.88)	<0.0001
HLI—total^d^	1 (ref)	0.71 (0.65–0.78)	0.58 (0.53–0.64)	0.46 (0.41–0.50)	0.74 (0.71–0.76)	<0.0001
WCRF^e^	1 (ref)	0.78 (0.71–0.85)	0.71 (0.64–0.78)	0.56 (0.51–0.62)	0.80 (0.77–0.83)	<0.0001
**Cancer mortality**						
MDS	1 (ref)	0.92 (0.86–0.98)	0.85 (0.80–0.91)	0.75 (0.70–0.80)	0.90 (0.88–0.92)	<0.0001
rMED	1 (ref)	0.87 (0.82–0.92)	0.79 (0.75–0.85)	0.73 (0.69–0.78)	0.88 (0.86–0.90)	<0.0001
MSDPS	1 (ref)	0.92 (0.87–0.98)	0.87 (0.81–0.92)	0.77 (0.72–0.82)	0.91 (0.88–0.93)	<0.0001
DQI-I	1 (ref)	0.82 (0.77–0.87)	0.73 (0.69–0.78)	0.66 (0.62–0.71)	0.86 (0.84–0.88)	<0.0001
HNFI	1 (ref)	0.94 (0.88–1.00)	0.84 (0.79–0.90)	0.77 (0.72–0.82)	0.90 (0.88–0.93)	<0.0001
HEI 2010	1 (ref)	0.88 (0.83–0.94)	0.78 (0.74–0.84)	0.77 (0.72–0.82)	0.88 (0.86–0.90)	<0.0001
WHO HDI	1 (ref)	0.93 (0.87–1.00)	0.86 (0.80–0.92)	0.84 (0.78–0.90)	0.94 (0.92–0.96)	0.0013
DASH	1 (ref)	0.89 (0.84–0.95)	0.85 (0.80–0.90)	0.75 (0.70–0.80)	0.89 (0.87–0.91)	<0.0001
HLI–diet	1 (ref)	0.86 (0.81–0.92)	0.81 (0.76–0.86)	0.76 (0.72–0.81)	0.90 (0.88–0.92)	0.0002
HLI—total^d^	1 (ref)	0.75 (0.70–0.80)	0.67 (0.62–0.71)	0.56 (0.52–0.60)	0.80 (0.78–0.82)	<0.0001
WCRF^e^	1 (ref)	0.85 (0.79–0.90)	0.76 (0.71–0.81)	0.70 (0.65–0.75)	0.86 (0.84–0.88)	<0.0001

**Abbreviations**: MDS, Mediterranean Diet Scale; rMED, relative Mediterranean diet score; MSDPS, Mediterranean Style Dietary Pattern Score; DQI-I, Diet Quality Index–International; HNFI, Healthy Nordic Food Index; HEI-2010, Healthy Eating Index 2010; WHO HDI, World Health Organization Healthy Diet Index; DASH, Dietary Approaches to Stop Hypertension; HLI, Healthy Lifestyle Index; HLI-diet, diet component of the HLI; WCRF, World Cancer Research Fund / American Institute for Cancer Research; Q, quartile of diet quality score

^**a**^ Model including only the dietary score as a predictor and age, stratified by sex and study center

^b^ HR for the increase of 1 SD of score

^c^ p-value for linear trend across quartiles

^d^ n = 376,553

^e^ n = 363,207

**Table 4:** Multivariate hazard ratios (Model 2)^a^ for 10-year mortality risk by quartile of diet quality and for a 1SD increase of score among 451,256 participants of the EPIC study.

	HR	HR (95% CI)	HR (95% CI)	HR (95% CI)	HR (95% CI)	P trend^c^
All-cause mortality	Q1	Q2	Q3	Q4	Continuous^b^	
MDS	1 (ref)	0.90 (0.86–0.94)	0.84 (0.81–0.88)	0.79 (0.76–0.83)	0.91 (0.90–0.93)	<0.0001
rMED	1 (ref)	0.87 (0.83–0.91)	0.81 (0.77–0.84)	0.77 (0.73–0.81)	0.89 (0.88–0.91)	<0.0001
MSDPS	1 (ref)	0.92 (0.89–0.96)	0.88 (0.84–0.92)	0.80 (0.76–0.84)	0.92 (0.90–0.93)	<0.0001
DQI-I	1 (ref)	0.89 (0.85–0.93)	0.81 (0.77–0.85)	0.75 (0.72–0.79)	0.90 (0.88–0.91)	<0.0001
HNFI	1 (ref)	0.94 (0.90–0.98)	0.87 (0.83–0.91)	0.83 (0.79–0.87)	0.93 (0.92–0.95)	<0.0001
HEI 2010	1 (ref)	0.89 (0.85–0.93)	0.84 (0.80–0.88)	0.82 (0.78–0.86)	0.91 (0.90–0.93)	0.0004
WHO HDI	1 (ref)	0.93 (0.89–0.98)	0.88 (0.84–0.92)	0.88 (0.84–0.92)	0.95 (0.94–0.97)	0.01
DASH	1 (ref)	0.90 (0.87–0.94)	0.85 (0.81–0.89)	0.82 (0.78–0.86)	0.92 (0.90–0.93)	<0.0001
HLI–diet	1 (ref)	0.91 (0.88–0.96)	0.86 (0.83–0.90)	0.83 (0.79–0.87)	0.93 (0.92–0.95)	<0.0001
HLI—total^d^	1 (ref)	0.75 (0.72–0.79)	0.65 (0.62–0.68)	0.55 (0.53–0.58)	0.79 (0.78–0.80)	<0.0001
WCRF^e^	1 (ref)	0.87 (0.83–0.91)	0.78 (0.74–0.81)	0.70 (0.67–0.74)	0.86 (0.85–0.88)	<0.0001
**CVD mortality**						
MDS	1 (ref)	0.81 (0.74–0.88)	0.80 (0.73–0.87)	0.80 (0.73–0.88)	0.91 (0.88–0.94)	0.79
rMED	1 (ref)	0.88 (0.81–0.96)	0.80 (0.73–0.88)	0.77 (0.71–0.85)	0.89 (0.86–0.92)	0.01
MSDPS	1 (ref)	0.93 (0.85–1.01)	0.90 (0.83–0.99)	0.81 (0.74–0.89)	0.92 (0.89–0.95)	0.004
DQI-I	1 (ref)	0.91 (0.83–0.99)	0.80 (0.73–0.88)	0.76 (0.69–0.83)	0.90 (0.87–0.93)	0.0002
HNFI	1 (ref)	0.91 (0.83–1.00)	0.88 (0.81–0.97)	0.82 (0.74–0.90)	0.93 (0.90–0.96)	0.03
HEI 2010	1 (ref)	0.89 (0.82–0.97)	0.85 (0.78–0.93)	0.82 (0.75–0.90)	0.93 (0.90–0.96)	0.10
WHO HDI	1 (ref)	0.95 (0.87–1.04)	0.88 (0.80–0.96)	0.84 (0.76–0.92)	0.93 (0.90–0.96)	0.01
DASH	1 (ref)	0.86 (0.79–0.94)	0.81 (0.74–0.88)	0.77 (0.70–0.84)	0.90 (0.87–0.93)	0.02
HLI–diet	1 (ref)	0.93 (0.85–1.01)	0.85 (0.77–0.93)	0.78 (0.71–0.86)	0.91 (0.88–0.94)	0.0003
HLI—total^d^	1 (ref)	0.72 (0.66–0.79)	0.59 (0.54–0.65)	0.47 (0.42–0.52)	0.74 (0.72–0.77)	<0.0001
WCRF^e^	1 (ref)	0.81 (0.74–0.88)	0.76 (0.69–0.84)	0.63 (0.57–0.70)	0.83 (0.81–0.86)	<0.0001
**Cancer mortality**						
MDS	1 (ref)	0.95 (0.89–1.01)	0.91 (0.85–0.96)	0.82 (0.77–0.88)	0.93 (0.91–0.95)	<0.0001
rMED	1 (ref)	0.92 (0.86–0.97)	0.86 (0.81–0.92)	0.82 (0.77–0.88)	0.92 (0.90–0.94)	0.001
MSDPS	1 (ref)	0.96 (0.91–1.02)	0.93 (0.87–0.99)	0.84 (0.79–0.90)	0.94 (0.92–0.96)	<0.0001
DQI-I	1 (ref)	0.88 (0.83–0.94)	0.83 (0.78–0.88)	0.78 (0.73–0.83)	0.91 (0.89–0.93)	0.0003
HNFI	1 (ref)	0.98 (0.92–1.05)	0.92 (0.86–0.98)	0.86 (0.80–0.92)	0.95 (0.92–0.97)	<0.0001
HEI 2010	1 (ref)	0.94 (0.88–1.00)	0.86 (0.81–0.92)	0.87 (0.81–0.92)	0.93 (0.90–0.95)	0.02
WHO HDI	1 (ref)	0.95 (0.89–1.02)	0.90 (0.84–0.96)	0.91 (0.85–0.97)	0.97 (0.94–0.99)	0.16
DASH	1 (ref)	0.94 (0.89–1.01)	0.94 (0.88–1.00)	0.87 (0.81–0.93)	0.94 (0.92–0.96)	0.01
HLI–diet	1 (ref)	0.91 (0.85–0.97)	0.88 (0.83–0.94)	0.86 (0.81–0.92)	0.94 (0.92–0.97)	0.10
HLI—total^d^	1 (ref)	0.75 (0.70–0.80)	0.67 (0.63–0.72)	0.57 (0.53–0.61)	0.80 (0.78–0.82)	<0.0001
WCRF^e^	1 (ref)	0.88 (0.82–0.94)	0.81 (0.76–0.87)	0.77 (0.72–0.83)	0.90 (0.88–0.92)	0.0003

**Abbreviations:** MDS, Mediterranean Diet Scale; rMED, relative Mediterranean diet score; MSDPS, Mediterranean Style Dietary Pattern Score; DQI-I, Diet Quality Index–International; HNFI, Healthy Nordic Food Index; HEI-2010, Healthy Eating Index 2010; WHO HDI, World Health Organization Healthy Diet Index; DASH, Dietary Approaches to Stop Hypertension; HLI, Healthy Lifestyle Index; HLI-diet, diet component of the HLI; WCRF, World Cancer Research Fund / American Institute for Cancer Research; Q, quartile of diet quality score

^**a**^ Model including the following predictors: dietary score and age at baseline, BMI (continuous), Physical activity (Cambridge index), smoking status (3 categories) and educational level, unless otherwise stated. Stratified by sex and study center.

^b^ HR for the increase of 1 SD of score

^c^ p-value for linear trend across quartiles

^d^ Model only including HLI, age and educational level because BMI, physical activity, smoking are components of the Healthy Lifestyle Index, n = 376,553

^e^ Model only including WCRF score, age, smoking and educational level as BMI and physical activity are components of the WCRF score, n = 363,207

Qualitatively similar results were observed for CVD mortality. All scores were inversely statistically significantly associated with CVD mortality in all models; HRs ranged between 0.89 (rMED) and 0.93 (HNFI, HEI-2010, WHO HDI) per SD increase of diet quality (Model 2).

All scores were also inversely associated with risk of cancer mortality but showed smaller effect sizes compared to total and CVD mortality; HRs ranged from 0.91 (DQI-I) to 0.97 (WHO HDI) (Model 2). The estimates were in the same range, although confidence intervals were wider, for mortality of obesity-related cancers (S2 Table). As observed in previous analyses, diet and lifestyle combined scores showed lower HRs than diet only: HLI (includes smoking) showed the strongest association (HR = 0.74 [0.72–0.77] for CVD and 0.80 [0.78–0.82] for cancer mortality). Further adjustment for total energy intake did not qualitatively change these results (S3 Table).

### Discrimination and calibration

**Fig 1:**
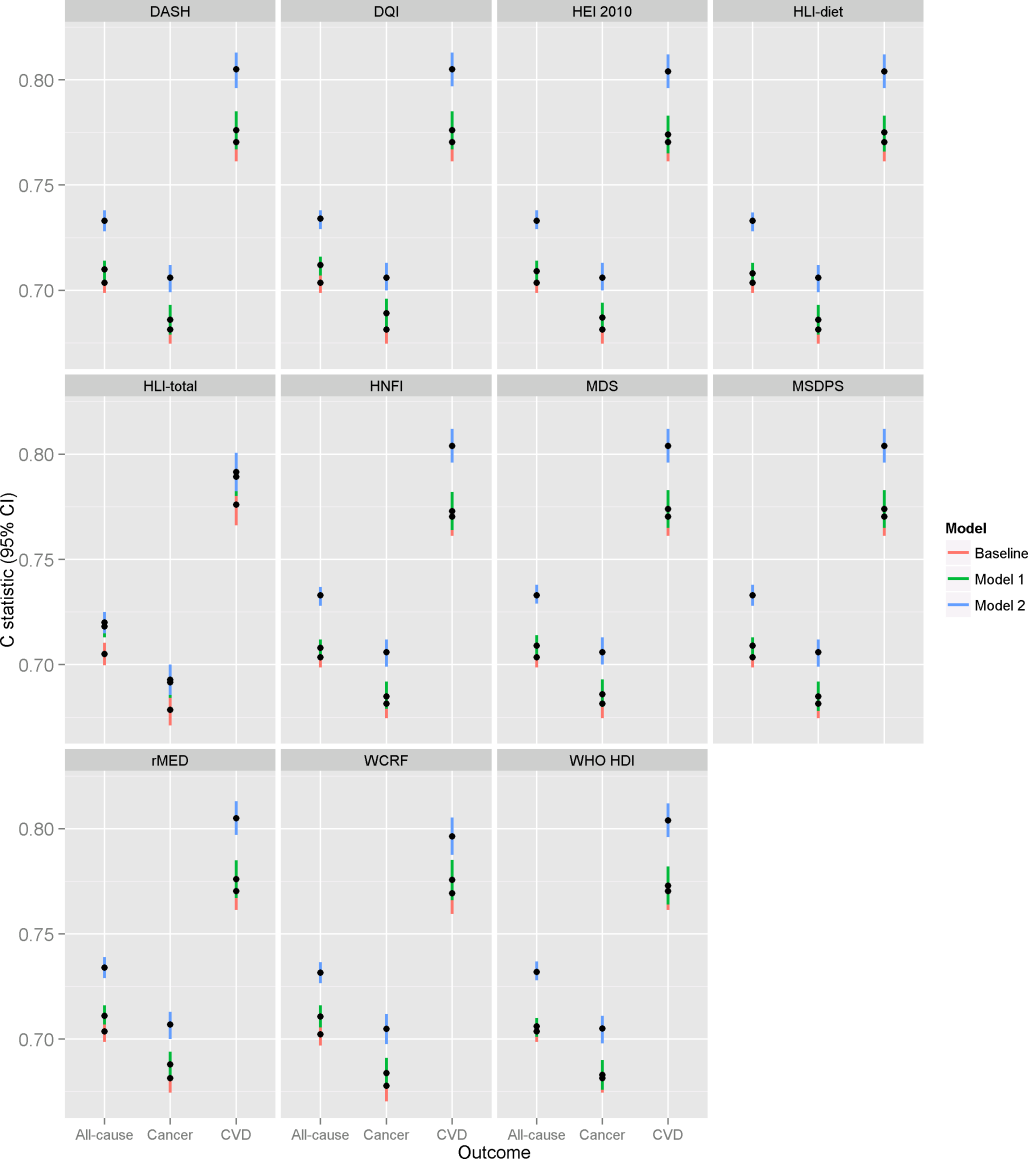
Discrimination (Harrell’s C statistic) of the baseline model ^a^, Model 1 ^b^ and Model 2 ^c^ for the prediction of 10-year mortality risk in 451,256 ^d,e^ participants to the EPIC study. Abbreviations: MDS, Mediterranean Diet Scale; rMED, relative Mediterranean diet score; MSDPS, Mediterranean Style Dietary Pattern Score; DQI-I, Diet Quality Index–International; HNFI, Healthy Nordic Food Index; HEI-2010, Healthy Eating Index 2010; WHO HDI, World Health Organization Healthy Diet Index; DASH, Dietary Approaches to Stop Hypertension; HLI, Healthy Lifestyle Index; HLI-diet, diet component of the HLI; WCRF, World Cancer Research Fund / American Institute for Cancer Research ^a^ Baseline model includes only age as a predictor, stratified by sex and center; ^b^ Model 1 = baseline + dietary score; ^c^ Model 2 = Model 1+ lifestyle factors: smoking, BMI, physical activity, educational level unless otherwise stated. ^d^ Model 2 = Model 1 + educational level because BMI, physical activity, smoking are components of the Healthy Lifestyle Index, n = 376,553. ^e^ Model 2 = Model 1 + smoking and educational level as BMI and physical activity are components of the WCRF score, n = 363,207.

The discrimination performance of dietary scores alone was low, with a C statistic ranging from 0.51 (HNFI for all-cause and cancer mortality) to 0.56 (rMED for CVD mortality), as presented in S4 Table. The discrimination performance of all models, for all three outcomes is presented in Fig 1 and S5 Table and compared to a baseline model only including age, stratified by sex and center. For all-cause mortality, in Model 1, i.e. considering predictive ability of the dietary score along with age, sex and center only, the discrimination was good for all dietary scores examined and the C-statistic ranged from 0.706 (WHO HDI) to 0.712 (DQI-I). Nonetheless, improved prediction compared to the baseline model of age and sex alone was small. Improvements of the C statistic were higher for DQI-I (difference in C statistic = 0.008), and for the two scores which include lifestyle components (WCRF (0.008) and HLI (0.013)). Addition of other lifestyle predictors (Model 2) further increased the C-statistic which now ranged from between 0.732 (WHO HDI) to 0.734 (DQI-I and rMED). Discrimination of all models was higher for CVD mortality and lower for cancer mortality compared to all-cause mortality. For CVD, dietary scores along with age and sex (Model 1) displayed very good discrimination with C-statistics ranging from 0.773 (WHO HDI, HNFI) to 0.776 (rMED, DQI-I and DASH). Those were improved in Model 2 reaching discrimination as high as 0.805 (DQI-I, rMED and DASH). For cancer, discrimination of the scores in Model 1 ranged between 0.685 (MSDPS, HNFI) to 0.689 (DQI-I). Again, addition of other lifestyle variables (Model 2) improved discrimination which reached 0.707 (rMED). In line with all-cause mortality analyses, dietary scores offered little improved prediction over the baseline model. For CVD, the difference was 0.006 (DQI-I), 0.013 (HLI) and 0.006 (WCRF) and for cancer 0.008 (DQI-I), 0.013 (HLI) and 0.006 (WCRF). Models predicting obesity-related cancers achieved slightly worse discrimination (median C-statistic 0.690 vs 0.706 in all cancer, S2 Table).

Calibration plots (S1 Fig) indicated that all models predicting all-cause, CVD and cancer mortality were well calibrated. The calibration slope was greater than 0.95 for all models (close to 1, which represents perfect calibration) (Model 2). The mean predicted-to-observed ratio, ranging from 0.97 to 0.98 for all scores, also indicated little evidence of under-prediction of the all-cause mortality risk. However, underestimation of the risk was observed for individuals classified as low risk, with a ratio ranging from 0.55 to 0.80, in the first three deciles of predicted risk in most models (corresponding to a mortality risk of <1%). The mean predicted-to-observed ratio was 0.93 for CVD and 0.95 for cancer mortality. The predicted (and observed) risk was <1% up to the 8^th^ decile for CVD and up to the 4^th^ decile for cancer mortality.

### Country-specific analyses

Differences in mean scores per country show consistently higher dietary scores (in particular MDS, rMED, MSDPS, HEI-2010 and DQI-I) in Mediterranean countries (Greece, Italy, Spain, France) and lower in the Scandinavian and north European region (Norway, Sweden, Denmark, Holland, UK and Germany) (S6 Table). Modest to high heterogeneity between countries in HR estimates for all-cause mortality was observed (S2 Fig). The I^2^ ranged 45% (HEI-2010) to 76% (MSDPS). Overall estimates by random-effect meta-analysis were similar to those obtained with the stratified model in the pooled analysis. Results for cancer and CVD mortality were comparable (S2 Fig); however, smaller heterogeneity was found overall for CVD mortality.

Heterogeneity between countries was very high with respect to discrimination (S3 Fig). For all-cause mortality, I^2^ was as high as 99% (Model 2) and C-statistic ranged from 0.65 (France) to 0.85 (UK-health conscious: Oxford). For CVD and cancer mortality, heterogeneity was elevated as well (I^2^ 97%); the C-statistic ranged from 0.72 (Denmark) to 0.92 (Oxford) for CVD and 0.63 (France) to 0.81 (Oxford) for cancer mortality. Forest plots are presented for DQI-I as the remaining diet-only quality scores presented comparable results, and for WCRF and HLI which include also lifestyle components. Calibration showed less heterogeneity across countries, with mean predicted-to-observed ratios ranging from 0.96 to 1.01 for all-cause mortality and 0.84 to 1.04 for cancer. CVD mortality models showed poorer calibration in Italy (predicted-to-observed ratio = 0.85) and Spain (0.83).

### Sex and age specific analyses

Similar hazard ratios were observed between men and women for all models and outcomes examined (S7 Table). Discrimination varied little by sex and age in relation to all-cause and cancer mortality. However, model discrimination for CVD mortality (S4 Fig) was consistently higher for women compared to men and for older (≥50 years) compared to younger (<50 years) participants, which can be partly explained by the very low number of CVD death cases at 10 years in the younger group compared to the older (n = 373 vs 3,388). In the younger age group, the added value of dietary scores (Model 1) and dietary scores along with lifestyle variables (Model 2) on top of age and sex was higher than that observed in the overall population for CVD mortality, with change in C statistic reaching 0.091 in <50 years vs 0.044 in ≥50 years for DQI-I in Model 2.

## Discussion

We present the first comparative examination of the association and predictive performance of 10 different diet quality scores for 10-year risk of all-cause, CVD and cancer mortality across 10 countries with over 450,000 participants without previous diagnosis of major diseases. Dietary scores, albeit strongly associated with mortality risk, are poor predictors of mortality when used in isolation. At the same time, models incorporating non-invasively assessed lifestyle predictors, including dietary scores, have good predictive ability (in terms of discrimination and calibration) for 10-year risk of mortality, both within and across countries, and for all outcomes examined (CVD, cancer and all-cause mortality).

As expected, age and sex were the strongest dominant predictors and dietary scores alone offered small added value in addition to these parameters. Given the fact that dietary assessment is challenging and prone to measurement error its role in risk assessment may be limited. However, dietary scores can provide personalized feedback and their role in lifestyle-based risk assessment through promoting behavior change and adherence to lifestyle modification for chronic disease prevention merits further investigation. As dietary scores are difficult to capture and assess, particularly when they include nutrient information, easy to measure food-based scores including only a limited number of components are preferable given that all scores examined here show comparable predictive performance. For instance, an adaptation of the DASH, MDS or HNFI (in the Northern countries), applying predefined rather than population-dependent cutoffs (e.g. Mediterranean diet score [12]), would be most pragmatic for individual risk prediction and health promotion.

Discrimination for 10-year risk of CVD was high (0.80), which is comparable to all major CVD risk prediction scores which include invasive measurements such as blood lipids and glucose [38–40]. Discrimination for all-cause and cancer mortality was also good and consistent with other risk scores used in clinical practice [41–43]. Cancer risk prediction models are often considered useful for early disease detection rather than disease prevention while primary prevention algorithms are only used for CVD in primary care [44]. However, we show here that lifestyle-based models may achieve good predictive ability for cancer mortality as well, and therefore could be useful for cancer prevention through targeted lifestyle modification. The fact that the same, easily-assessed and inexpensively measured risk factors could predict CVD and cancer mortality makes these scores promising for use in population-wide risk assessment. Still, such predictive models are showing better predictive value for CVD mortality compared to cancer mortality. This is probably driven by the weaker association of diet with all cancers combined compared to CVD. Previous studies found a weaker or null association of Mediterranean diet scores with cancer in many different populations [12], and similarly of four key diet quality indices in three U.S. cohorts as part of the Dietary Pattern Method Project [6,7,10]. Models limited to obesity-related cancers only did not show stronger association or discrimination, which can partly be explained by smaller sample size of these analyses.

Diet quality and their association with mortality risk varied substantially between European countries. For instance, a Mediterranean dietary pattern was strongly inversely associated with mortality in Spain but not in the Netherlands, where the Healthy Nordic Dietary Pattern was strongly associated with lower mortality. These results, reflecting cultural differences, e.g. in the much higher intake of olive oil in the Mediterranean countries, or dark bread or root vegetables in the northern European countries, can be useful to guide national dietary guidelines to fit in the cultural context and be relevant in the prevention of chronic disease mortality. Similarly, the prediction ability of scores varied widely across countries. This could be due to the differences in baseline risk in different populations (for calibration) and differences in measurement of predictors in different countries.

We observed that all models consistently underestimated the risk in the very low risk groups. This indicates that if such models would be used in practice they should be adapted in local circumstances and recalibrated to the outcome incidence observed in particular populations. This underestimation is unlikely to have clinical implication as the predicted and observed risk in the first third of the distribution were below 1% for all outcomes, hence the actual difference between observed and predicted risk was very small.

### Strengths and limitations

The main strength of this study is the large sample size that allowed investigating predictive value of dietary scores with adequate precision. However, several limitations should be noted. The first one is the absence of data on dietary sodium intake, which did not allow calculation of some of the original dietary scores, namely the HEI-2010, DQI-I, and DASH score, and did not capture all of the components of the diet intended by their authors. Moreover, dietary data are subject to measurement error, particularly when collected through food frequency questionnaires [45]; even if dietary data were calibrated in each center [22] we cannot preclude misclassification of dietary scores and that the observed associations with mortality were biased towards the null [45]. Dietary data in EPIC have been subject to thorough standardization across countries; however, differences in dietary questionnaires may still have affected the calculation and interpretation of some scores in different countries. Despite these sources of bias, we observed significant inverse associations between every dietary score and mortality and relatively high discrimination of the models. The estimated C-statistics are likely optimistic, however correction for optimism in such a large sample would only make a small difference [46].

### Conclusions

Our study is among the first to investigate the predictive value of dietary scores and their comparative value on mortality risk. We have shown that various dietary scores are associated with all-cause mortality, as well as cause-specific (CVD and cancer) mortality, with stronger associations with CVD compared to cancer. These scores have poor predictive performance for 10-year mortality risk when used in isolation. In combination with other non-invasive common risk factors such as age, sex, center, smoking, body weight, physical activity and educational level, these composite scores display good predictive ability. However, the increase in discriminatory predictive value for 10-year mortality (total, CVD, cancer) that would come from collecting dietary assessments is small when data on the other non-invasive measures are already accounted for. Potential use of dietary information in risk prediction scores, in the form of simplified food based dietary assessment which could be easily collected by individuals themselves or in primary care consultations, could be justified if dietary assessment is shown to enhance personalized feedback and motivate lifestyle modifications. The fact that diet and other lifestyle risk factors such as physical activity could be included within predictive models for both CVD and cancer mortality merits further consideration for population-wide screening or self-assessment for timely disease prevention. Impact studies are now needed to show the effect on risk communication of dietary and other lifestyle variables and to guide efficient chronic disease prevention in populations.

## Supporting Information

S1 Fig **Calibration plots for all diet and lifestyle quality scores associated with 10-year risk of all-cause (S1A Fig), CVD (S1B Fig), and cancer (S1C Fig) mortality. Observed risk is plotted against predicted risk in Model 2** ^**a**^**, by decile of predicted risk. S1D Fig is the calibration table and predicted/observed risk for DQI-I associated with 10-year risk of all-cause mortality.** ^a^ Model 2 includes the following predictors: age at baseline, BMI, Physical activity, smoking status and educational level, stratified by sex and study centre. For HLI total, the model only includes HLI, age and educational level because BMI, physical activity, smoking are components of the score. For WCRF, the model only includes the WCRF score, smoking and educational level as BMI and physical activity are components of the score.
(PDF)

S2 Fig Multivariate hazard ratios (Model 2: adjusted for age and lifestyle risk factors) for 10-year all-cause (S2A Fig), CVD (S2B Fig), and cancer (S2C Fig) mortality risk for a 1SD increase of score among 451,256 participants of the EPIC study, by country.
(PDF)

S3 Fig Discrimination (Harrell’s C statistic) of diet/lifestyle quality scores in Model 2 (predictors: age, diet quality score, physical activity, smoking, BMI, educational level, stratified by sex and center) for 10-year risk of all-cause (S3A Fig), CVD (S3B Fig), and cancer (S3C Fig) mortality among 451,256 participants of the EPIC study, by country.
(PDF)

S4 Fig **Cstatistic of the baseline model** ^**a**^**, Model 1** ^**b**^ **and Model 2** ^**c**^ **in 451,256 participants to the EPIC study, by sex (S4A Fig) and by age category (S4B Fig).** ^a^ Baseline model includes only age as a predictor, stratified by sex and center; ^b^ Model 1 also includes the dietary score; ^c^ Model 2 also includes lifestyle factors: smoking, BMI, physical activity, educational level for DQI-I, smoking and educational level for WCRF, educational level for HLI.
(PDF)

S1 File Details on recruitment of participants to the European Prospective Investigation into Cancer and Nutrition, selection of analysis sample and dietary data management. (PDF)

S1 Table Description of the food and nutrient components used for the calculation of the 11 dietary scores. + indicates positive weighting (encourages consumption);—indicates negative weighting (limits consumption).
(PDF)

S2 Table Multivariate hazard ratios and C-statistic (Model 2^a^) for 10-year mortality risk due to obesity-related cancer for a 1SD increase of score among 451,256 participants of the EPIC study. ^a^ Model including the following predictors: age at baseline, Physical activity (Cambridge index), smoking status (3 categories) and educational level, unless otherwise stated. Stratified by study center and sex. ^b^ HR for the increase of 1 SD of score. ^c^ p-value for linear trend across quartiles. ^d^ Model only including HLI, age and educational level because BMI, physical activity, smoking are components of the Healthy Lifestyle Index, n = 376,553. ^e^ Model only including WCRF score, smoking and educational level as BMI and physical activity are components of the WCRF score, n = 363,207.
(PDF)

S3 Table Multivariate hazard ratios adjusted for total energy intake a for 10-year mortality risk by quartile of score and for a 1SD increase of score among 451,256 participants of the EPIC study. ^a^ Model including the following predictors: dietary score, energy intake and age at baseline, BMI (continuous), Physical activity (Cambridge index), smoking status (3 categories) and educational level, unless otherwise stated. Stratified by sex and study center. ^b^ HR for the increase of 1 SD of score. ^c^ p-value for linear trend across quartiles. ^d^ Model only including HLI, age, energy intake and educational level because BMI, physical activity, smoking are components of the Healthy Lifestyle Index, n = 376,553. ^e^ Model only including WCRF score, age, energy intake, smoking and educational level as BMI and physical activity are components of the WCRF score, n = 363,207.
(PDF)

S4 Table C statistic of the baseline model ^a^, Model 1 ^b^ and Model 2 ^c^ for the prediction of 10-year mortality risk in 451,256 participants to the EPIC study. ^a^ Baseline model includes only age as a predictor, stratified by sex and center; ^b^ Model 1 = baseline + dietary score; ^c^ Model 2 = Model 1+ lifestyle factors: smoking, BMI, physical activity, educational level unless otherwise stated. ^d^ Model 2 = Model 1 + educational level because BMI, physical activity, smoking are components of the Healthy Lifestyle Index, n = 376,553. ^e^ Model 2 = Model 1 + smoking and educational level as BMI and physical activity are components of the WCRF score, n = 363,207.
(PDF)

S5 Table C statistic of the dietary scores alone for 10-year risk of all-cause, CVD and cancer mortality in 451,256 participants to the EPIC study. (PDF)

S6 Table Geographical differences in dietary scores across EPIC centres. (PDF)

S7 Table Age and sex specific multivariate hazard ratios (Model 2: adjusted for age and lifestyle risk factors) for 10-year mortality risk for a 1SD increase of score among 451,256 participants of the EPIC study. ^**a**^ Model including the following predictors: age at baseline, BMI (continuous), Physical activity (Cambridge index), smoking status (3 categories) and educational level, unless otherwise stated. Stratified by study center. ^**b**^ Model including the following predictors: age at baseline, BMI (continuous), Physical activity (Cambridge index), smoking status (3 categories) and educational level, unless otherwise stated. Stratified by sex and study center. ^c^ HR for the increase of 1 SD of score. ^d^ Model only including HLI, age and educational level because BMI, physical activity, smoking are components of the Healthy Lifestyle Index, n = 376,553. ^e^ Model only including WCRF score, smoking and educational level as BMI and physical activity are components of the WCRF score, n = 363,207.
(PDF)
