# Supplementary material for: Diet Quality Scores and Prediction of All-Cause, Cardiovascular and Cancer Mortality in a Pan-European Cohort Study
Source: PLoS One. 2016 Jul 13;11(7):e0159025. doi: 10.1371/journal.pone.0159025 (PMC4943719; doi:10.1371/journal.pone.0159025)
Supplement: S1 Fig — Calibration plots for all diet and lifestyle quality scores associated with 10-year risk of all-cause (S1A Fig), CVD (S1B Fig), and cancer (S1C Fig) mortality. Observed risk is plotted against predicted risk in Model 2 a, by decile of predicted risk. S1D Fig is the calibration table and predicted/observed risk for DQI-I associated with 10-year risk of all-cause mortality. a Model 2 includes the following predictors: age at baseline, BMI, Physical activity, smoking status and educational level, stratified by sex and study centre. For HLI total, the model only includes HLI, age and educational level because BMI, physical activity, smoking are components of the score. For WCRF, the model only includes the WCRF score, smoking and educational level as BMI and physical activity are components of the score. (PDF) [file pone.0159025.s001.pdf]

S1a Fig. Calibration plots for all diet and lifestyle quality scores associated with 10-year risk of all-cause mortality among 451,256 participants of the EPIC study

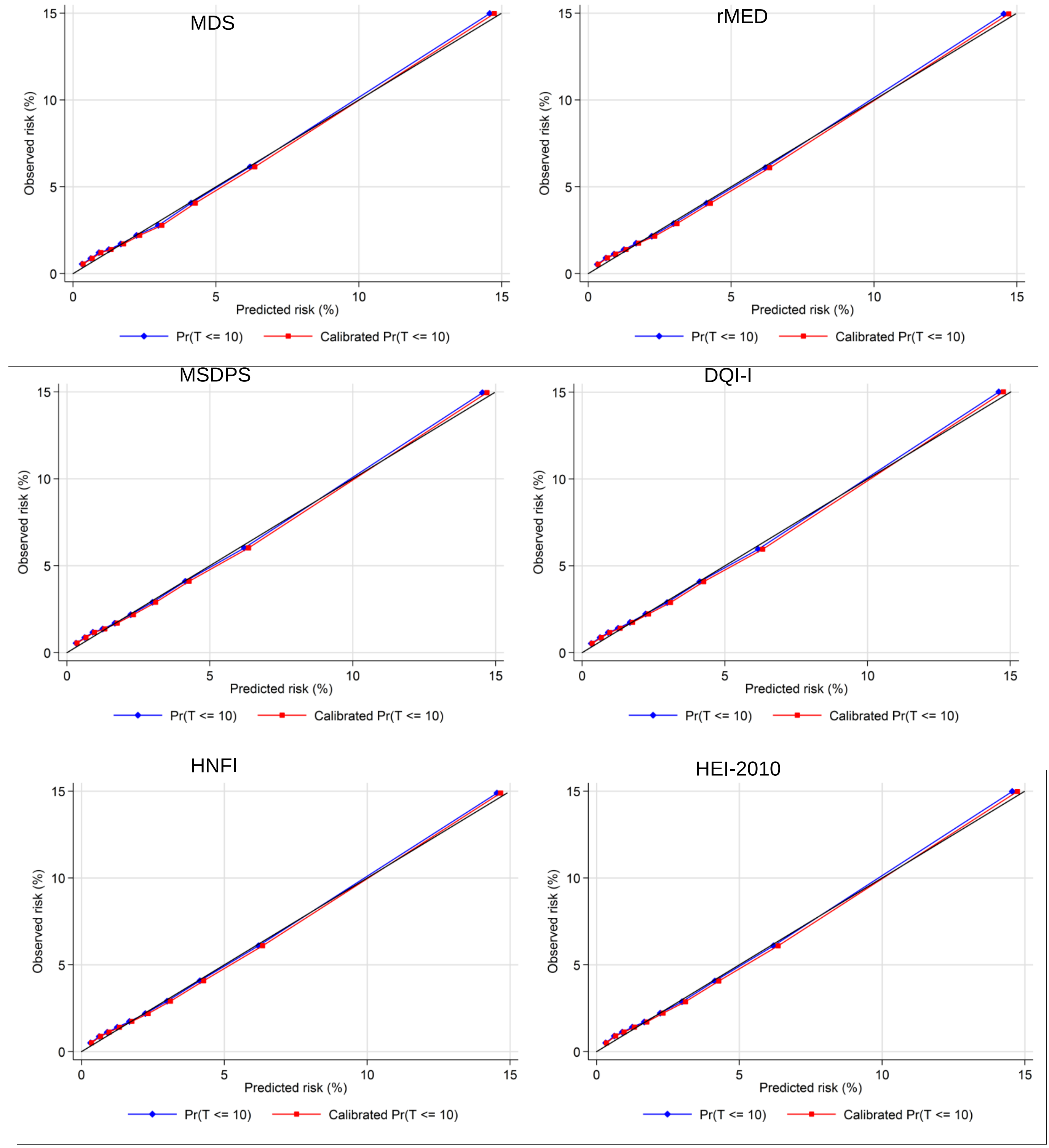

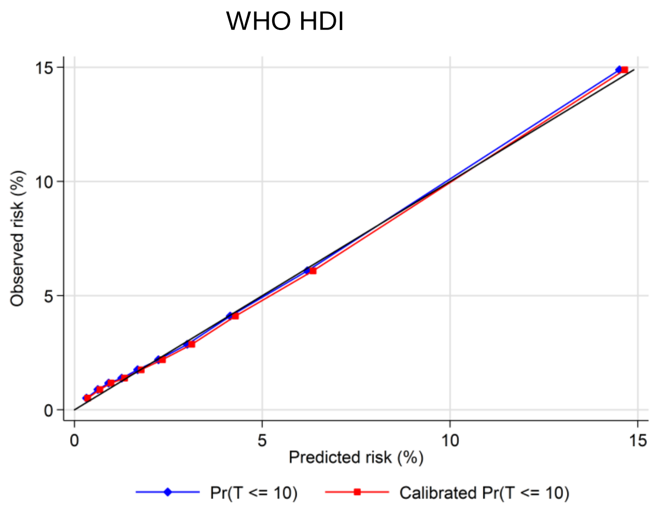
WHO HDI
Observed risk (%)
Predicted risk (%)
0
5
10
15
Pr(T <= 10)
Calibrated Pr(T <= 10)

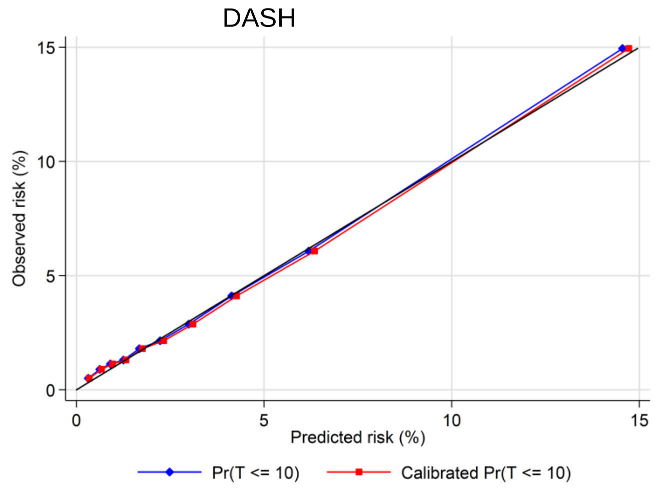
DASH
Observed risk (%)
Predicted risk (%)
0
5
10
15
Pr(T <= 10)
Calibrated Pr(T <= 10)

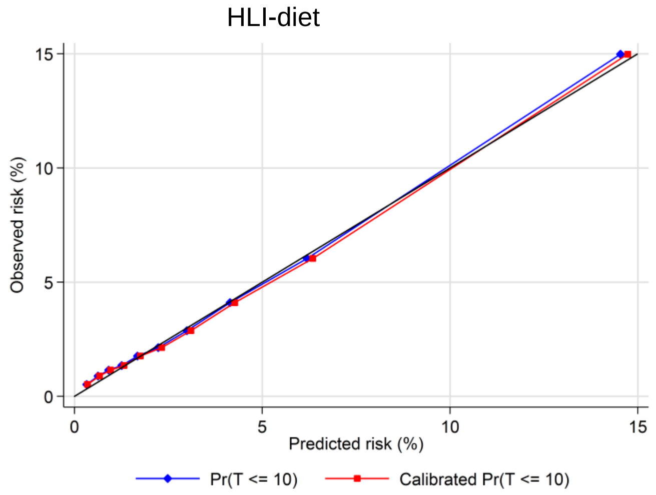
HLI-diet
Observed risk (%)
Predicted risk (%)
0
5
10
15
Pr(T <= 10)
Calibrated Pr(T <= 10)

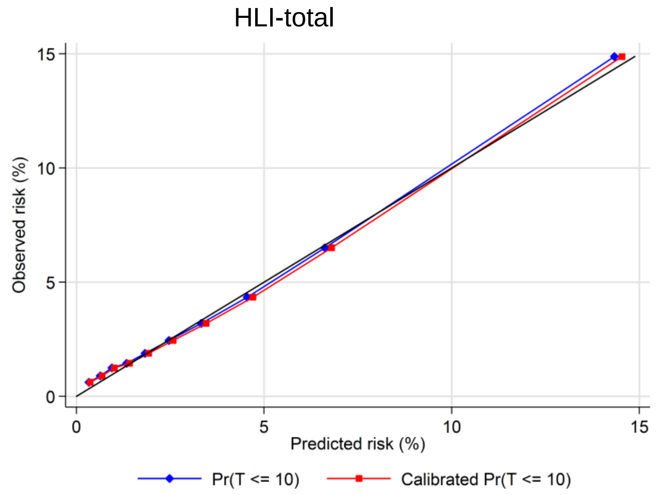
HLI-total
Observed risk (%)
Predicted risk (%)
0
5
10
15
Pr(T <= 10)
Calibrated Pr(T <= 10)

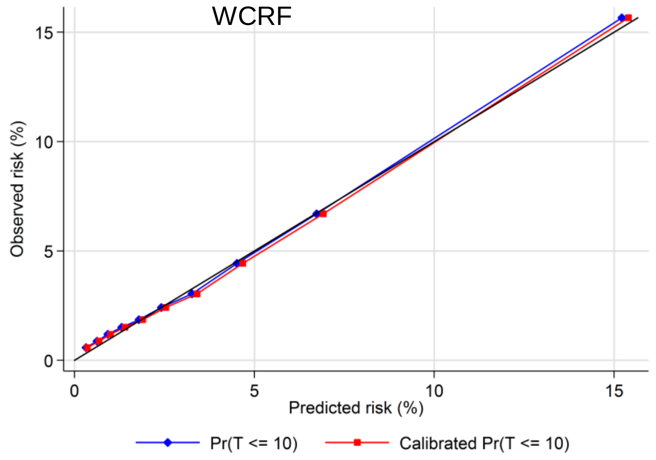
WCRF
Observed risk (%)
Predicted risk (%)
0
5
10
15
Pr(T <= 10)
Calibrated Pr(T <= 10)

S1b Fig. Calibration plots for all diet and lifestyle quality scores associated with 10-year risk of CVD mortality among 451,256 participants of the EPIC study.

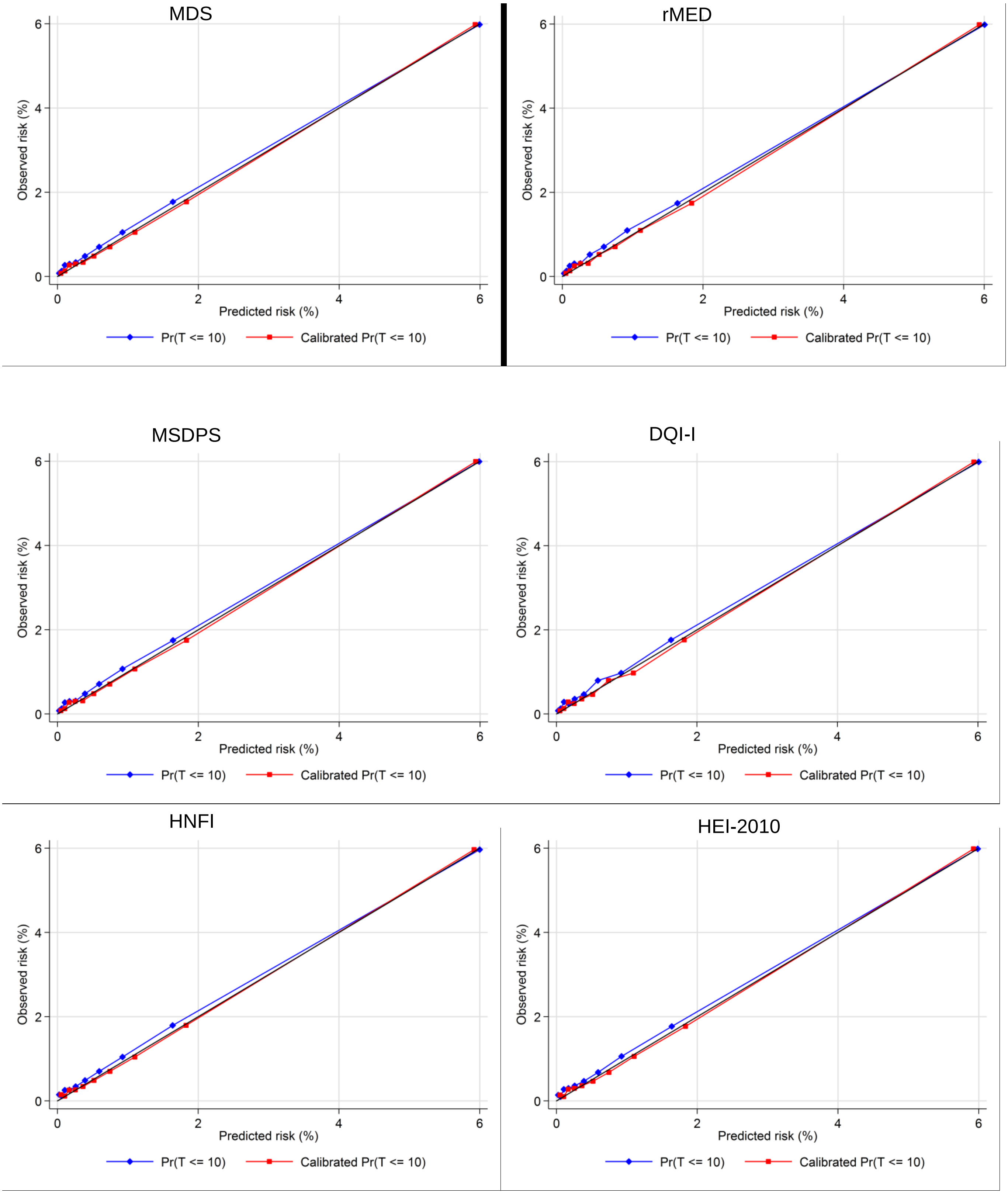

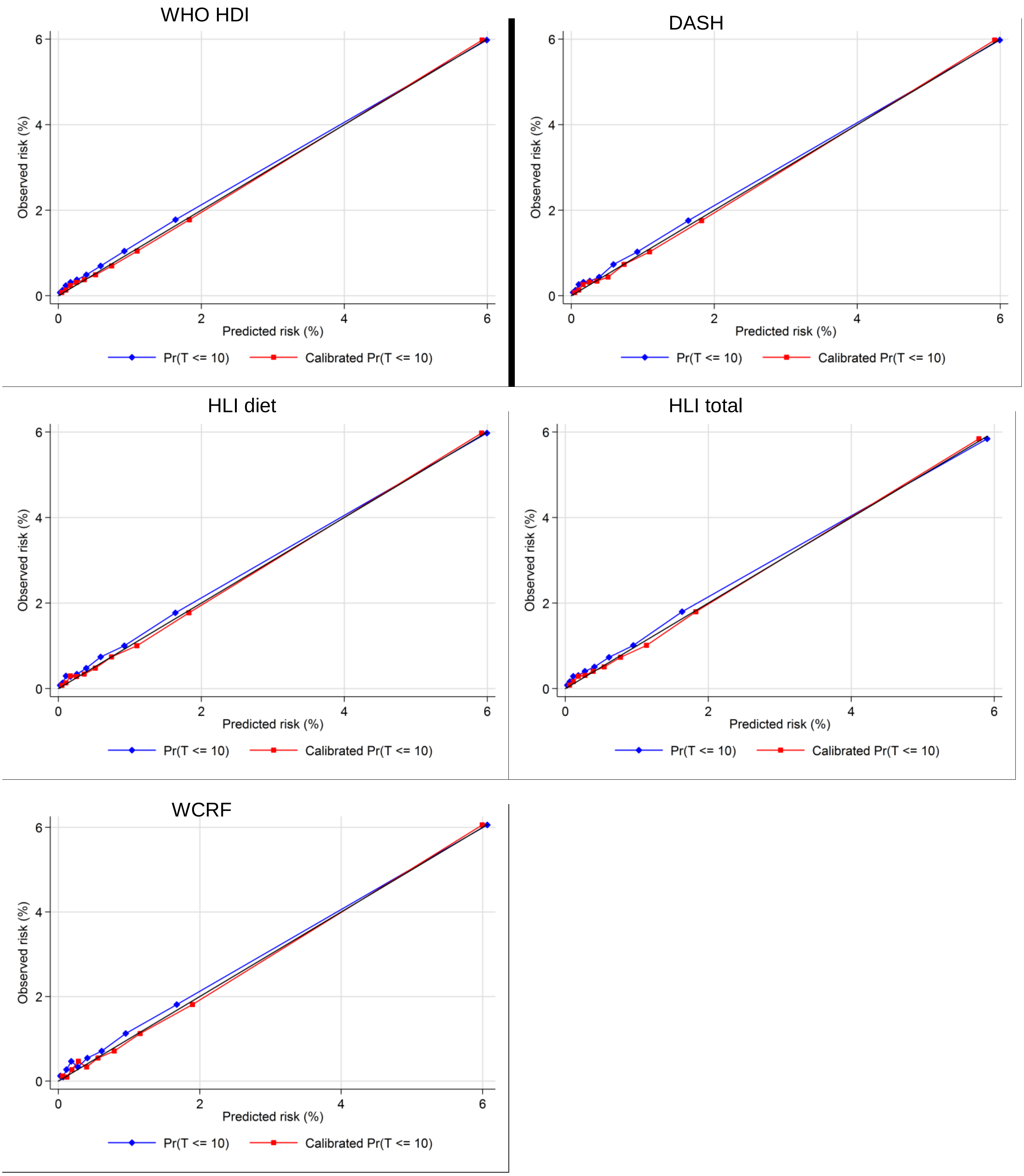
WHO HDI
DASH
HLI diet
HLI total
WCRF
Observed risk (%)
Predicted risk (%)
0
2
4
6
Pr(T <= 10)
Calibrated Pr(T <= 10)

S1c Fig. Calibration plots for all diet and lifestyle quality scores associated with 10-year risk of cancer mortality among 451,256 participants of the EPIC study.

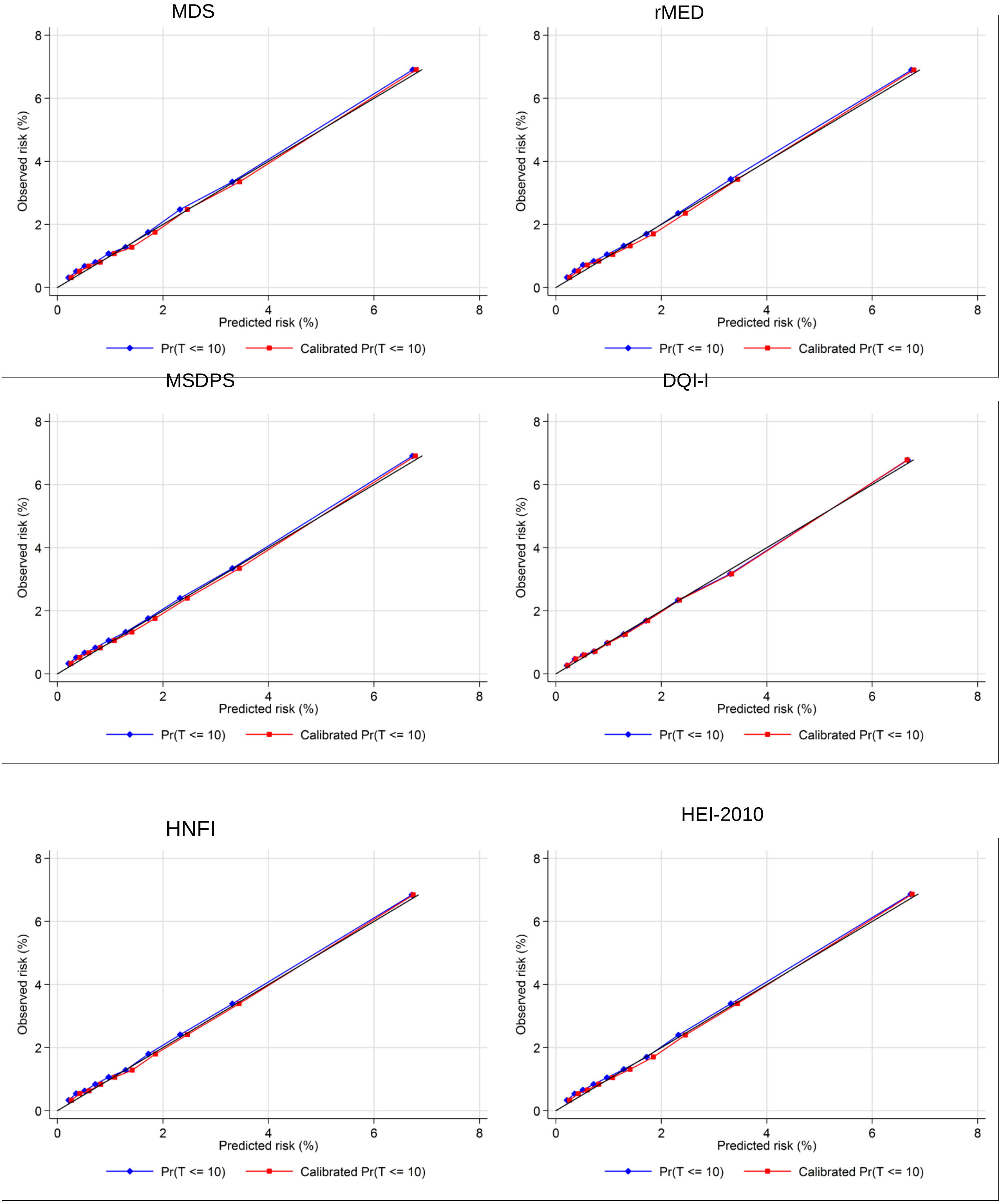

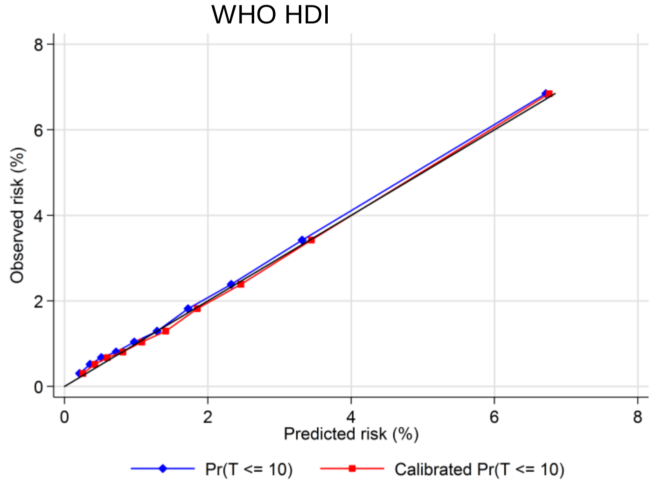
WHO HDI
Observed risk (%)
Predicted risk (%)
0
2
4
6
8
Pr(T <= 10)
Calibrated Pr(T <= 10)

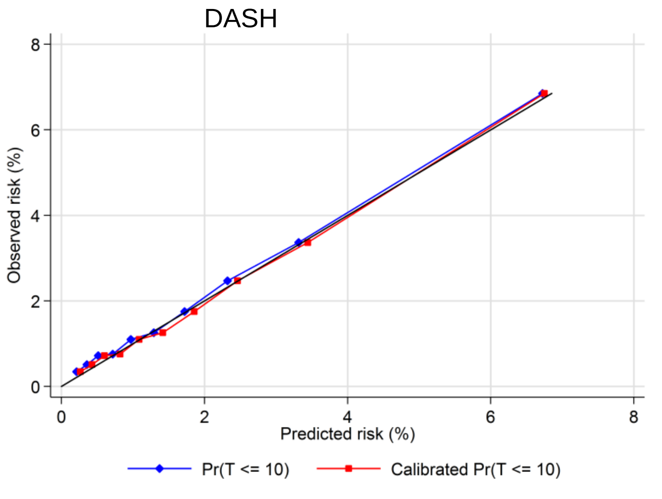
DASH
Observed risk (%)
Predicted risk (%)
0
2
4
6
8
Pr(T <= 10)
Calibrated Pr(T <= 10)

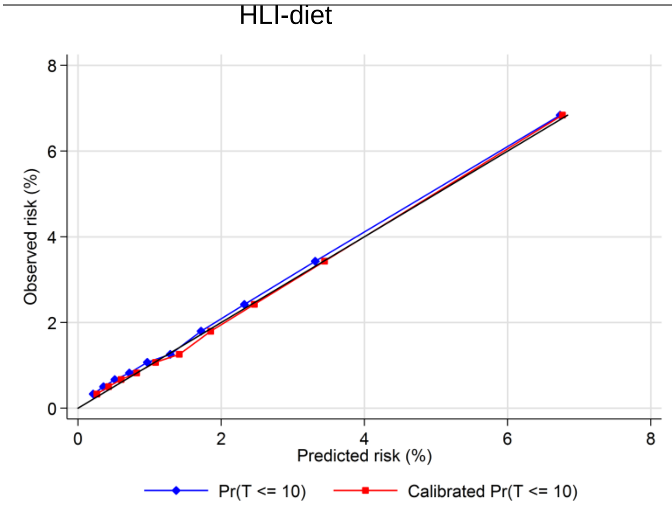
HLI-diet
Observed risk (%)
Predicted risk (%)
0
2
4
6
8
Pr(T <= 10)
Calibrated Pr(T <= 10)

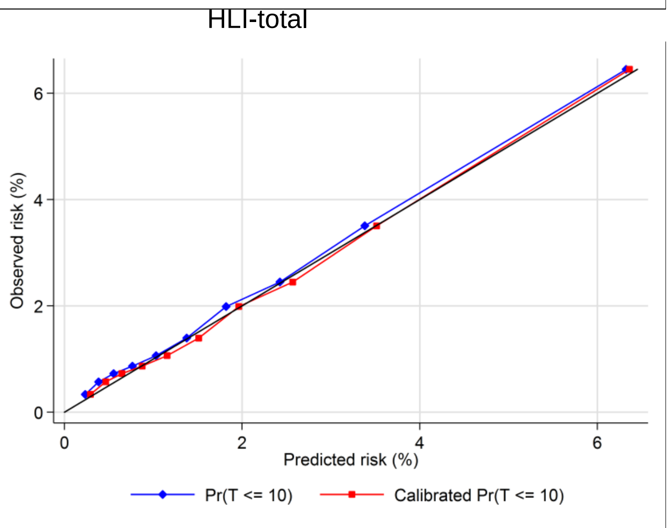
HLI-total
Observed risk (%)
Predicted risk (%)
0
2
4
6
Pr(T <= 10)
Calibrated Pr(T <= 10)

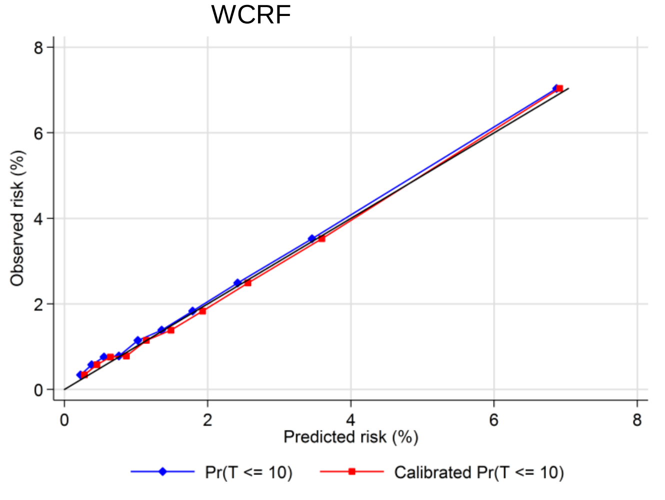
WCRF
Observed risk (%)
Predicted risk (%)
0
2
4
6
8
Pr(T <= 10)
Calibrated Pr(T <= 10)

S1d Fig. Calibration table and predicted/observed risk for DQI-I associated with 10-year risk of all-cause mortality

Decile of predicted risk	Predicted risk (%)	Observed risk (%)	Number of events at 10 years	Pred/Obs ratio
1	0.32	0.54	194	0.59
2	0.63	0.91	362	0.69
3	0.91	1.13	453	0.81
4	1.26	1.39	569	0.9
5	1.68	1.75	736	0.96
6	2.23	2.16	916	1.03
7	2.99	2.88	1237	1.04
8	4.14	4.05	1769	1.02
9	6.20	6.10	2665	1.02
10	14.55	14.96	6299	0.97

Mean Predicted/Observed ratio = 0.97

Calibration slope = 0.99
