# Supplementary material for: Diet Quality Scores and Prediction of All-Cause, Cardiovascular and Cancer Mortality in a Pan-European Cohort Study
Source: PLoS One. 2016 Jul 13;11(7):e0159025. doi: 10.1371/journal.pone.0159025 (PMC4943719; doi:10.1371/journal.pone.0159025)
Supplement: S2 Fig — Multivariate hazard ratios (Model 2: adjusted for age and lifestyle risk factors) for 10-year all-cause (S2A Fig), CVD (S2B Fig), and cancer (S2C Fig) mortality risk for a 1SD increase of score among 451,256 participants of the EPIC study, by country. (PDF) [file pone.0159025.s002.pdf]

S2a Fig. Multivariate hazard ratios (Model 2: adjusted for age and lifestyle risk factors) for 10-year all-cause mortality risk for a 1SD increase of score among 451,256 participants of the EPIC study, by country

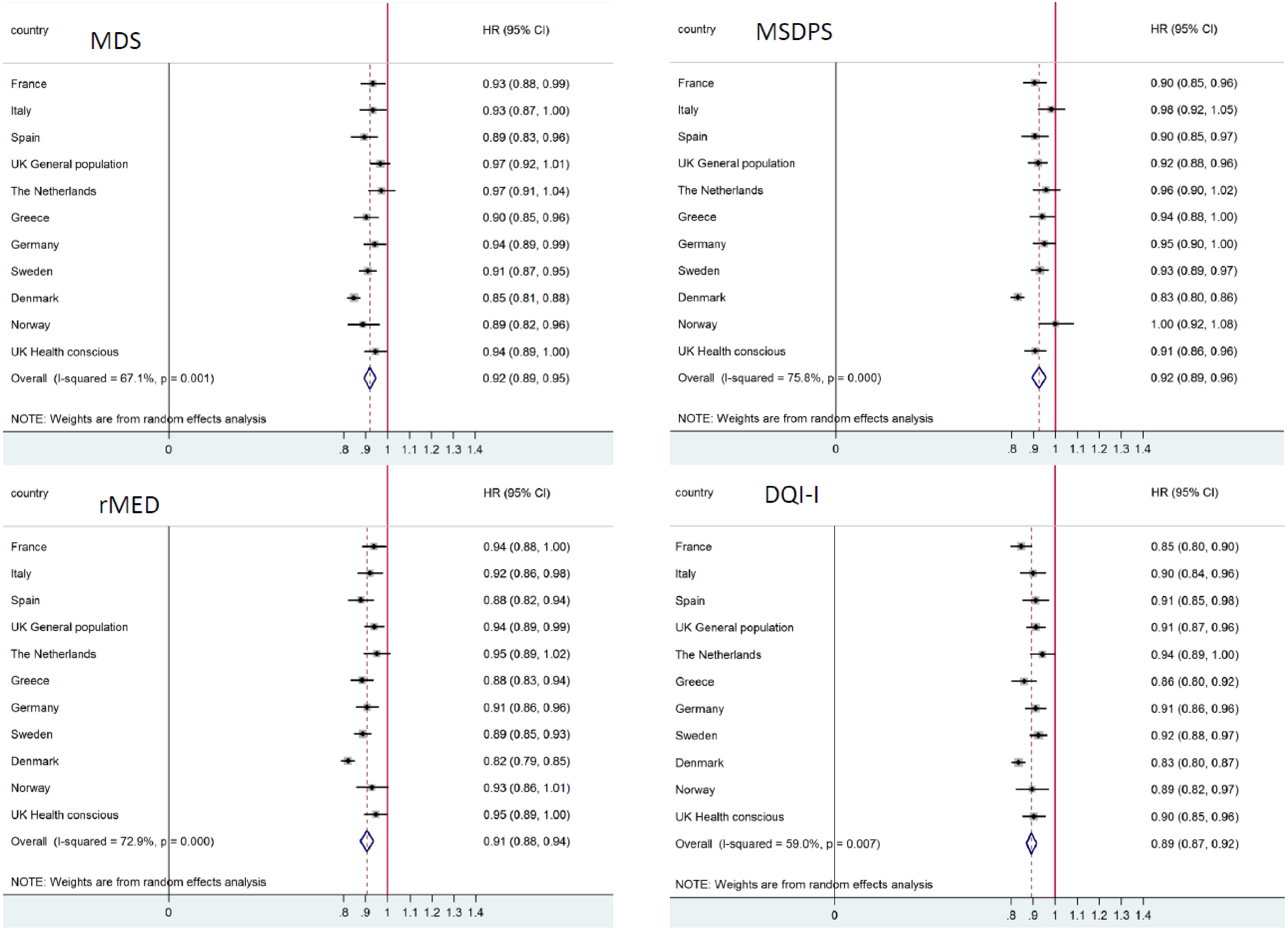

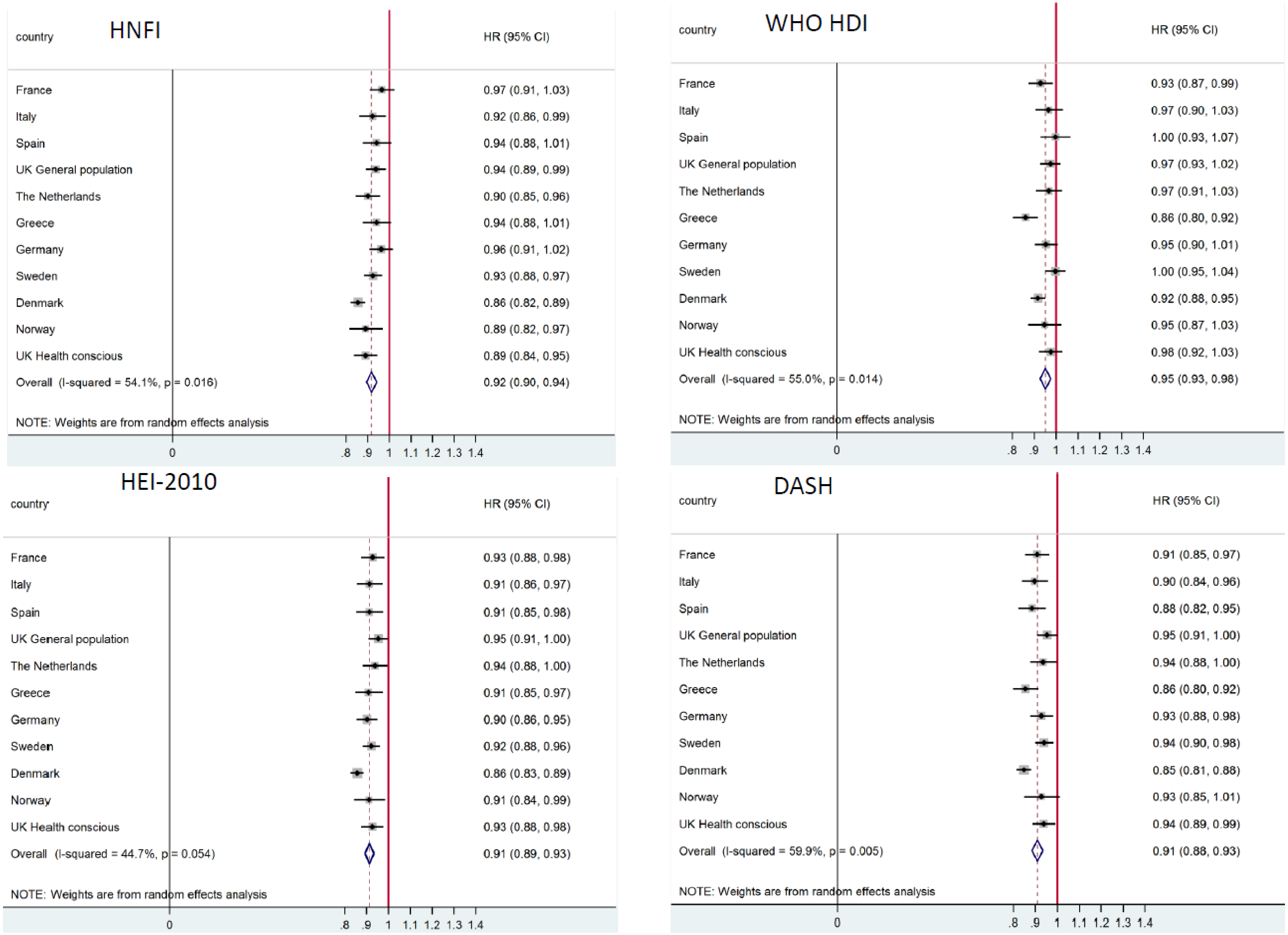
HNFI
country
HR (95% CI)
France 0.97 (0.91, 1.03)
Italy 0.92 (0.86, 0.99)
Spain 0.94 (0.88, 1.01)
UK General population 0.94 (0.89, 0.99)
The Netherlands 0.90 (0.85, 0.96)
Greece 0.94 (0.88, 1.01)
Germany 0.96 (0.91, 1.02)
Sweden 0.93 (0.88, 0.97)
Denmark 0.86 (0.82, 0.89)
Norway 0.89 (0.82, 0.97)
UK Health conscious 0.89 (0.84, 0.95)
Overall (I-squared = 54.1%, p = 0.016) 0.92 (0.90, 0.94)
NOTE: Weights are from random effects analysis
0 .8 .9 1 1.1 1.2 1.3 1.4
WHO HDI
country
HR (95% CI)
France 0.93 (0.87, 0.99)
Italy 0.97 (0.90, 1.03)
Spain 1.00 (0.93, 1.07)
UK General population 0.97 (0.93, 1.02)
The Netherlands 0.97 (0.91, 1.03)
Greece 0.86 (0.80, 0.92)
Germany 0.95 (0.90, 1.01)
Sweden 1.00 (0.95, 1.04)
Denmark 0.92 (0.88, 0.95)
Norway 0.95 (0.87, 1.03)
UK Health conscious 0.98 (0.92, 1.03)
Overall (I-squared = 55.0%, p = 0.014) 0.95 (0.93, 0.98)
NOTE: Weights are from random effects analysis
0 .8 .9 1 1.1 1.2 1.3 1.4
HEI-2010
country
HR (95% CI)
France 0.93 (0.88, 0.98)
Italy 0.91 (0.86, 0.97)
Spain 0.91 (0.85, 0.98)
UK General population 0.95 (0.91, 1.00)
The Netherlands 0.94 (0.88, 1.00)
Greece 0.91 (0.85, 0.97)
Germany 0.90 (0.86, 0.95)
Sweden 0.92 (0.88, 0.96)
Denmark 0.86 (0.83, 0.89)
Norway 0.91 (0.84, 0.99)
UK Health conscious 0.93 (0.88, 0.98)
Overall (I-squared = 44.7%, p = 0.054) 0.91 (0.89, 0.93)
NOTE: Weights are from random effects analysis
0 .8 .9 1 1.1 1.2 1.3 1.4
DASH
country
HR (95% CI)
France 0.91 (0.85, 0.97)
Italy 0.90 (0.84, 0.96)
Spain 0.88 (0.82, 0.95)
UK General population 0.95 (0.91, 1.00)
The Netherlands 0.94 (0.88, 1.00)
Greece 0.86 (0.80, 0.92)
Germany 0.93 (0.88, 0.98)
Sweden 0.94 (0.90, 0.98)
Denmark 0.85 (0.81, 0.88)
Norway 0.93 (0.85, 1.01)
UK Health conscious 0.94 (0.89, 0.99)
Overall (I-squared = 59.9%, p = 0.005) 0.91 (0.88, 0.93)
NOTE: Weights are from random effects analysis
0 .8 .9 1 1.1 1.2 1.3 1.4

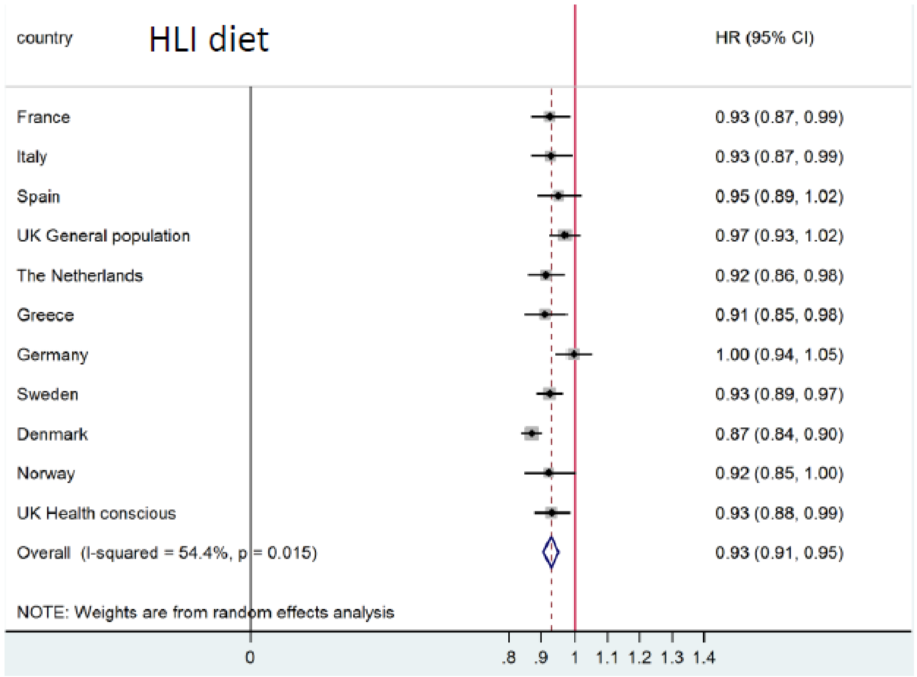
country
HLI diet
HR (95% CI)
France 0.93 (0.87, 0.99)
Italy 0.93 (0.87, 0.99)
Spain 0.95 (0.89, 1.02)
UK General population 0.97 (0.93, 1.02)
The Netherlands 0.92 (0.86, 0.98)
Greece 0.91 (0.85, 0.98)
Germany 1.00 (0.94, 1.05)
Sweden 0.93 (0.89, 0.97)
Denmark 0.87 (0.84, 0.90)
Norway 0.92 (0.85, 1.00)
UK Health conscious 0.93 (0.88, 0.99)
Overall (I-squared = 54.4%, p = 0.015) 0.93 (0.91, 0.95)
NOTE: Weights are from random effects analysis
0
.8
.9
1
1.1
1.2
1.3
1.4

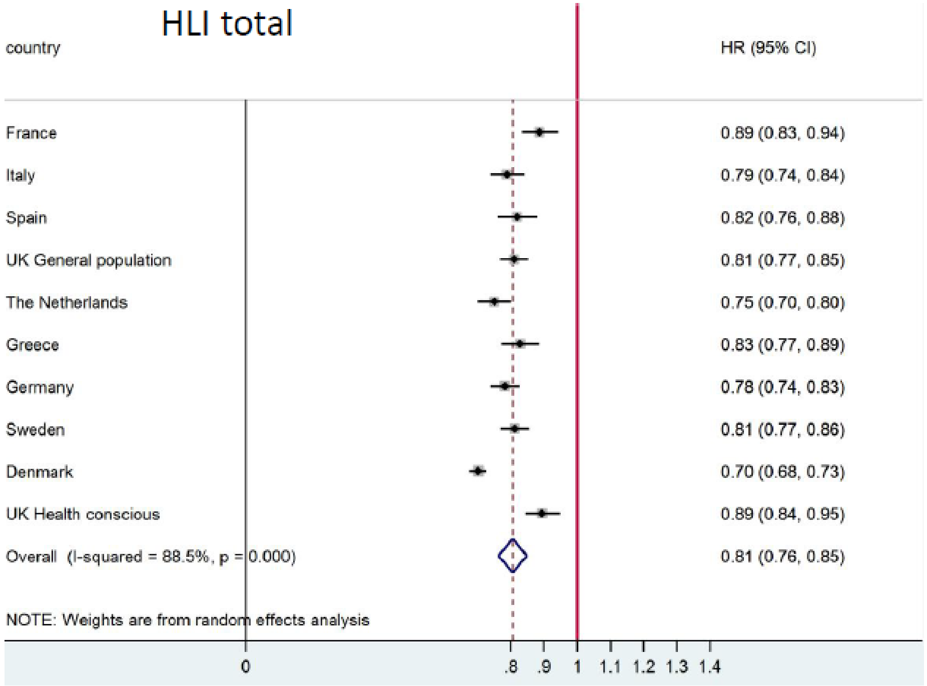
HLI total
country
HR (95% CI)
France 0.89 (0.83, 0.94)
Italy 0.79 (0.74, 0.84)
Spain 0.82 (0.76, 0.88)
UK General population 0.81 (0.77, 0.85)
The Netherlands 0.75 (0.70, 0.80)
Greece 0.83 (0.77, 0.89)
Germany 0.78 (0.74, 0.83)
Sweden 0.81 (0.77, 0.86)
Denmark 0.70 (0.68, 0.73)
UK Health conscious 0.89 (0.84, 0.95)
Overall (I-squared = 88.5%, p = 0.000) 0.81 (0.76, 0.85)
NOTE: Weights are from random effects analysis
0
.8
.9
1
1.1
1.2
1.3
1.4

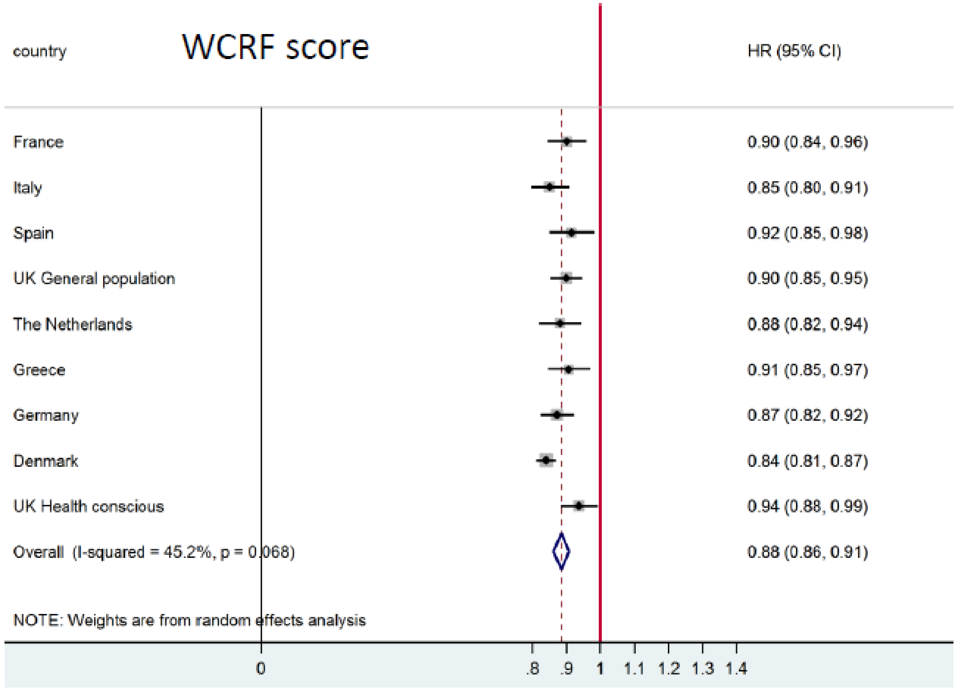
country
WCRF score
HR (95% CI)
France 0.90 (0.84, 0.96)
Italy 0.85 (0.80, 0.91)
Spain 0.92 (0.85, 0.98)
UK General population 0.90 (0.85, 0.95)
The Netherlands 0.88 (0.82, 0.94)
Greece 0.91 (0.85, 0.97)
Germany 0.87 (0.82, 0.92)
Denmark 0.84 (0.81, 0.87)
UK Health conscious 0.94 (0.88, 0.99)
Overall (I-squared = 45.2%, p = 0.068) 0.88 (0.86, 0.91)
NOTE: Weights are from random effects analysis
0
.8
.9
1
1.1
1.2
1.3
1.4

S2b Fig. Multivariate hazard ratios (Model 2: adjusted for age and lifestyle risk factors) for 10-year CVD mortality risk for a 1SD increase of score among 451,256 participants of the EPIC study, by country.

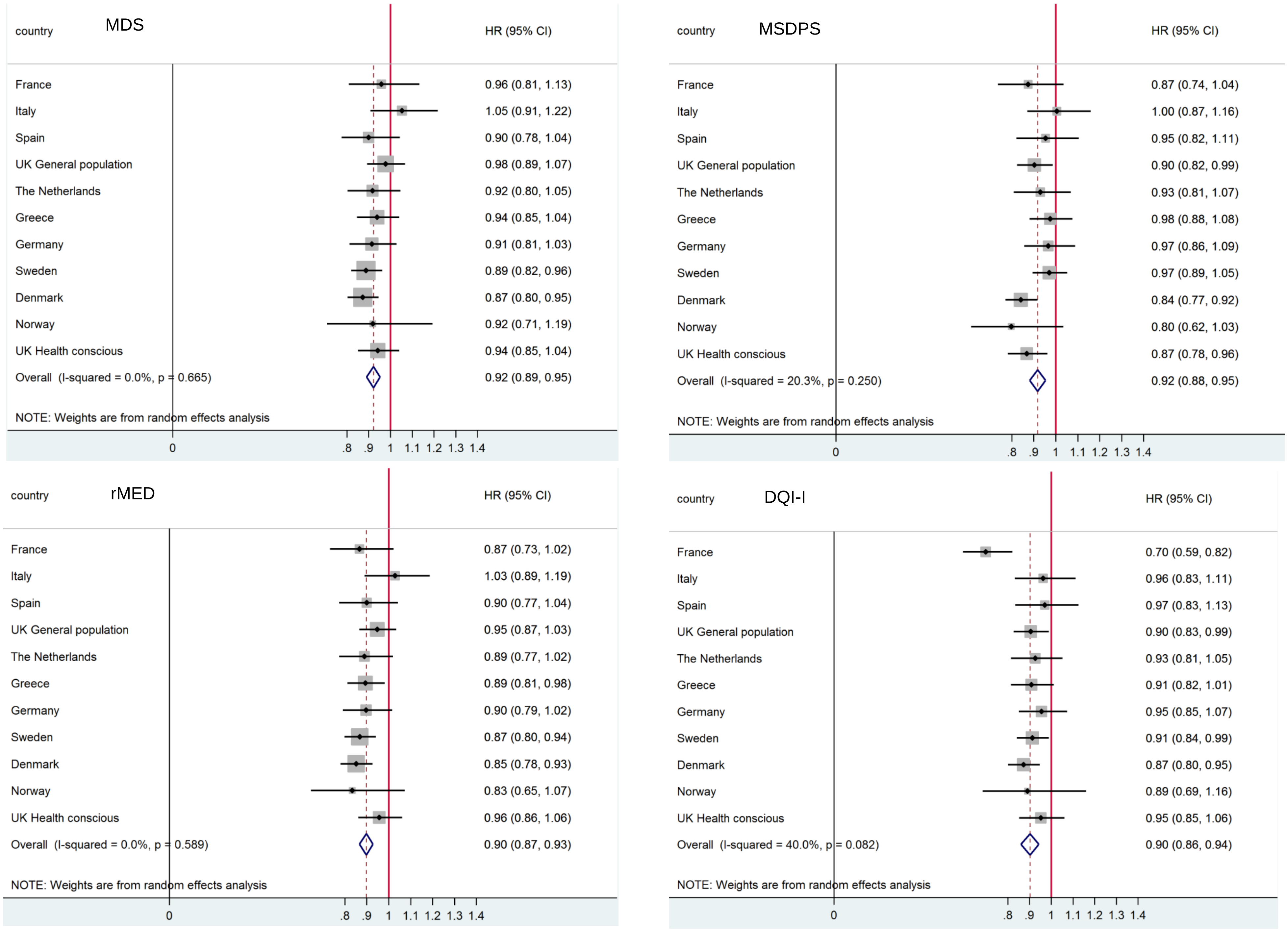

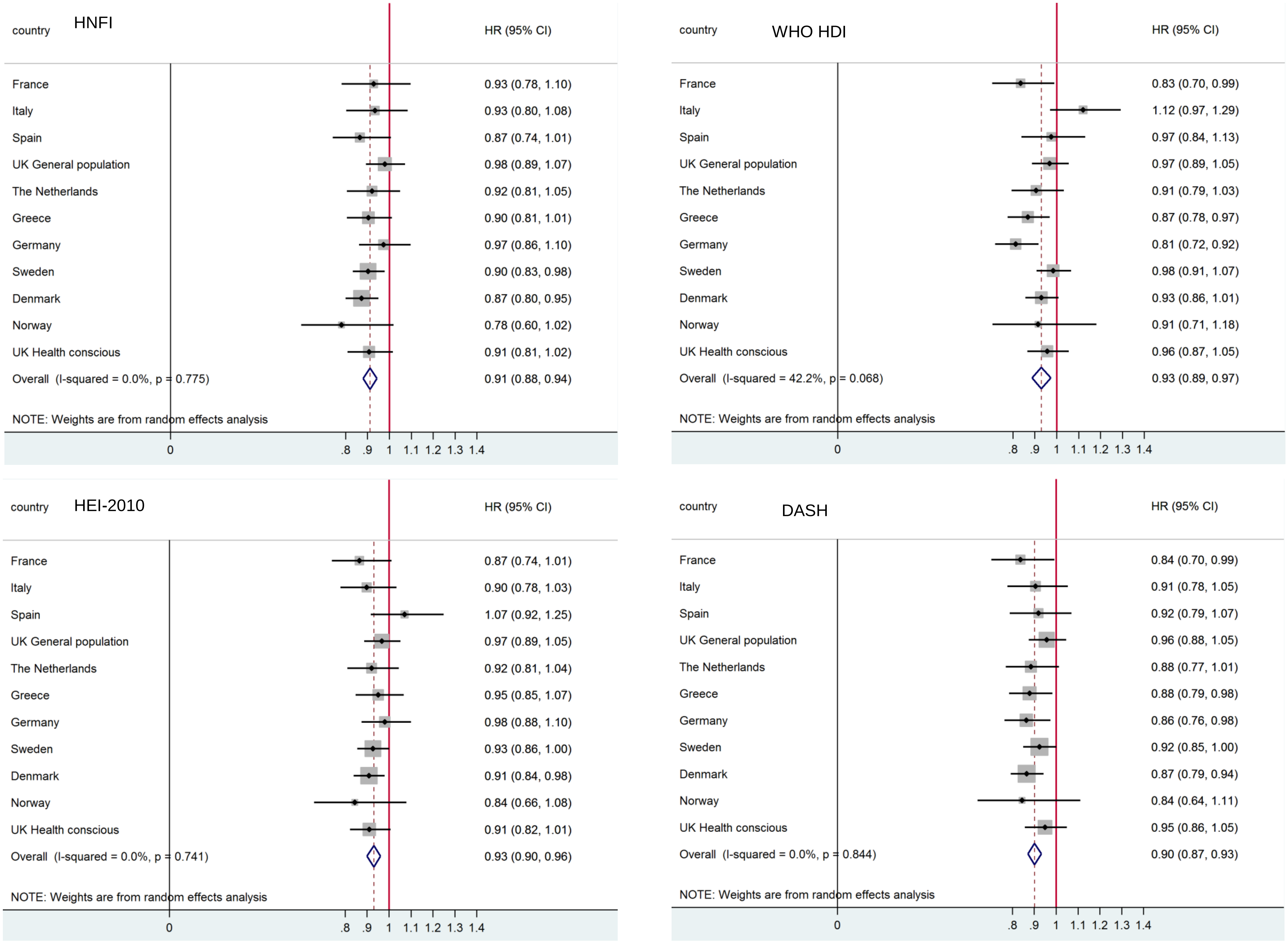

country
HNFI
HR (95% CI)
France 0.93 (0.78, 1.10)
Italy 0.93 (0.80, 1.08)
Spain 0.87 (0.74, 1.01)
UK General population 0.98 (0.89, 1.07)
The Netherlands 0.92 (0.81, 1.05)
Greece 0.90 (0.81, 1.01)
Germany 0.97 (0.86, 1.10)
Sweden 0.90 (0.83, 0.98)
Denmark 0.87 (0.80, 0.95)
Norway 0.78 (0.60, 1.02)
UK Health conscious 0.91 (0.81, 1.02)
Overall (I-squared = 0.0%, p = 0.775) 0.91 (0.88, 0.94)
NOTE: Weights are from random effects analysis
0 .8 .9 1 1.1 1.2 1.3 1.4
country
WHO HDI
HR (95% CI)
France 0.83 (0.70, 0.99)
Italy 1.12 (0.97, 1.29)
Spain 0.97 (0.84, 1.13)
UK General population 0.97 (0.89, 1.05)
The Netherlands 0.91 (0.79, 1.03)
Greece 0.87 (0.78, 0.97)
Germany 0.81 (0.72, 0.92)
Sweden 0.98 (0.91, 1.07)
Denmark 0.93 (0.86, 1.01)
Norway 0.91 (0.71, 1.18)
UK Health conscious 0.96 (0.87, 1.05)
Overall (I-squared = 42.2%, p = 0.068) 0.93 (0.89, 0.97)
NOTE: Weights are from random effects analysis
0 .8 .9 1 1.1 1.2 1.3 1.4
country
HEI-2010
HR (95% CI)
France 0.87 (0.74, 1.01)
Italy 0.90 (0.78, 1.03)
Spain 1.07 (0.92, 1.25)
UK General population 0.97 (0.89, 1.05)
The Netherlands 0.92 (0.81, 1.04)
Greece 0.95 (0.85, 1.07)
Germany 0.98 (0.88, 1.10)
Sweden 0.93 (0.86, 1.00)
Denmark 0.91 (0.84, 0.98)
Norway 0.84 (0.66, 1.08)
UK Health conscious 0.91 (0.82, 1.01)
Overall (I-squared = 0.0%, p = 0.741) 0.93 (0.90, 0.96)
NOTE: Weights are from random effects analysis
0 .8 .9 1 1.1 1.2 1.3 1.4
country
DASH
HR (95% CI)
France 0.84 (0.70, 0.99)
Italy 0.91 (0.78, 1.05)
Spain 0.92 (0.79, 1.07)
UK General population 0.96 (0.88, 1.05)
The Netherlands 0.88 (0.77, 1.01)
Greece 0.88 (0.79, 0.98)
Germany 0.86 (0.76, 0.98)
Sweden 0.92 (0.85, 1.00)
Denmark 0.87 (0.79, 0.94)
Norway 0.84 (0.64, 1.11)
UK Health conscious 0.95 (0.86, 1.05)
Overall (I-squared = 0.0%, p = 0.844) 0.90 (0.87, 0.93)
NOTE: Weights are from random effects analysis
0 .8 .9 1 1.1 1.2 1.3 1.4

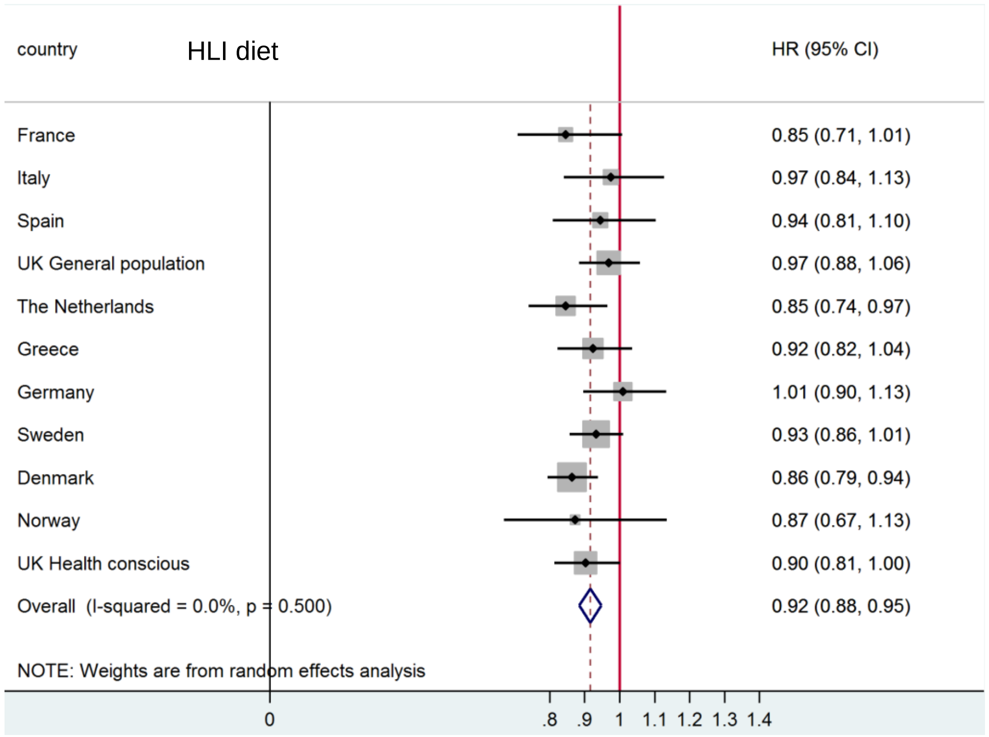
country
HLI diet
HR (95% CI)
France 0.85 (0.71, 1.01)
Italy 0.97 (0.84, 1.13)
Spain 0.94 (0.81, 1.10)
UK General population 0.97 (0.88, 1.06)
The Netherlands 0.85 (0.74, 0.97)
Greece 0.92 (0.82, 1.04)
Germany 1.01 (0.90, 1.13)
Sweden 0.93 (0.86, 1.01)
Denmark 0.86 (0.79, 0.94)
Norway 0.87 (0.67, 1.13)
UK Health conscious 0.90 (0.81, 1.00)
Overall (I-squared = 0.0%, p = 0.500) 0.92 (0.88, 0.95)
NOTE: Weights are from random effects analysis
0 .8 .9 1 1.1 1.2 1.3 1.4

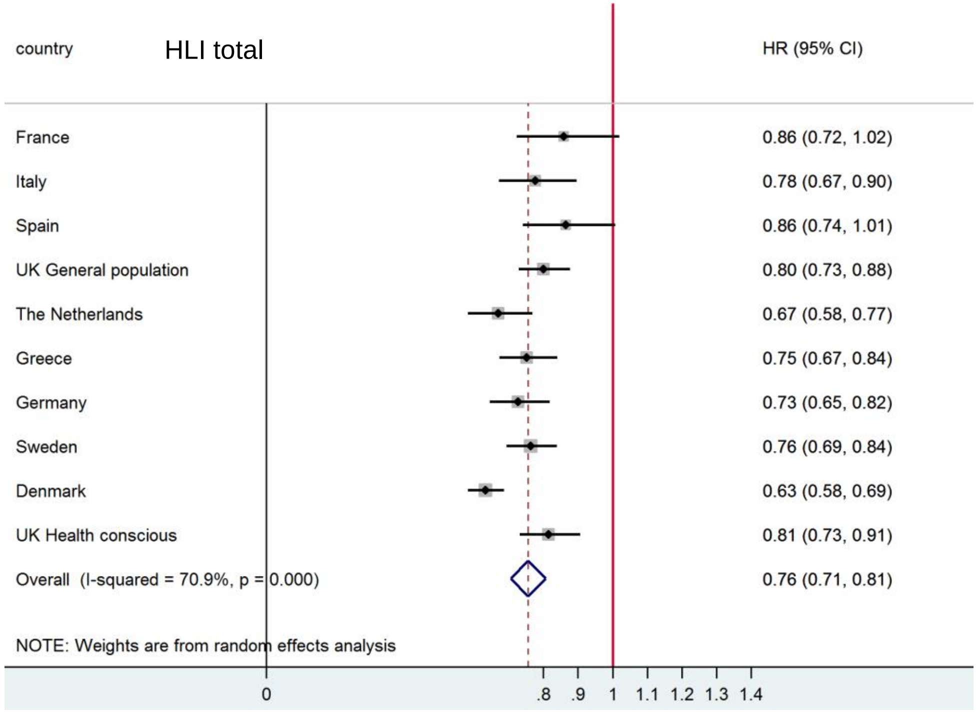
country
HLI total
HR (95% CI)
France 0.86 (0.72, 1.02)
Italy 0.78 (0.67, 0.90)
Spain 0.86 (0.74, 1.01)
UK General population 0.80 (0.73, 0.88)
The Netherlands 0.67 (0.58, 0.77)
Greece 0.75 (0.67, 0.84)
Germany 0.73 (0.65, 0.82)
Sweden 0.76 (0.69, 0.84)
Denmark 0.63 (0.58, 0.69)
UK Health conscious 0.81 (0.73, 0.91)
Overall (I-squared = 70.9%, p = 0.000) 0.76 (0.71, 0.81)
NOTE: Weights are from random effects analysis
0 .8 .9 1 1.1 1.2 1.3 1.4

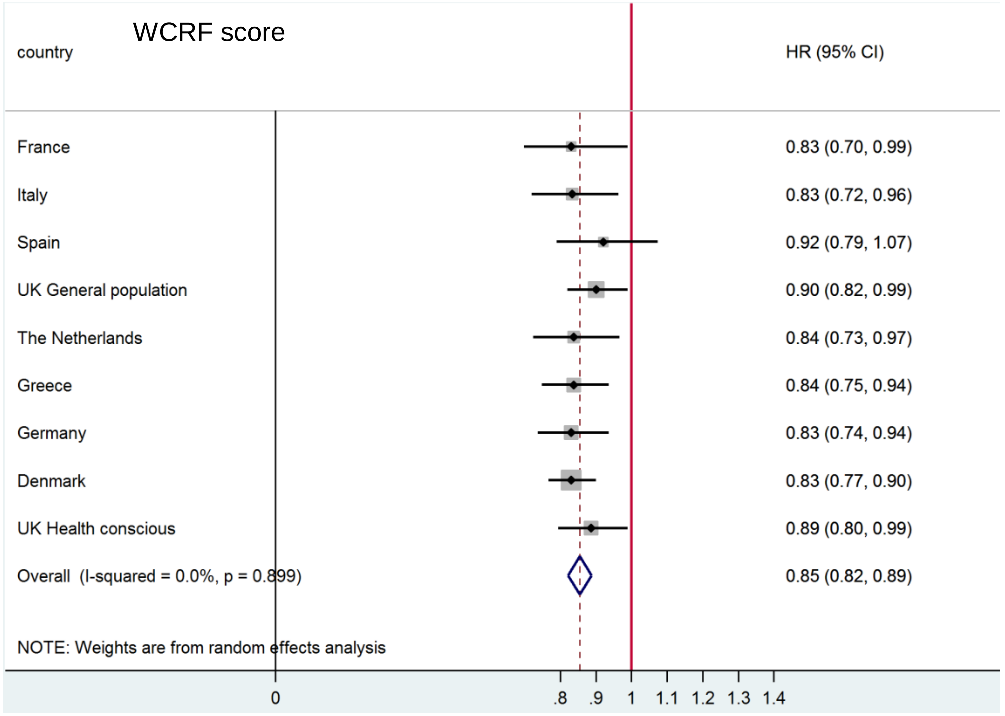
WCRF score
country
HR (95% CI)
France 0.83 (0.70, 0.99)
Italy 0.83 (0.72, 0.96)
Spain 0.92 (0.79, 1.07)
UK General population 0.90 (0.82, 0.99)
The Netherlands 0.84 (0.73, 0.97)
Greece 0.84 (0.75, 0.94)
Germany 0.83 (0.74, 0.94)
Denmark 0.83 (0.77, 0.90)
UK Health conscious 0.89 (0.80, 0.99)
Overall (I-squared = 0.0%, p = 0.899) 0.85 (0.82, 0.89)
NOTE: Weights are from random effects analysis
0 .8 .9 1 1.1 1.2 1.3 1.4

S2c Fig. Multivariate hazard ratios (Model 2: adjusted for age and lifestyle risk factors) for 10-year cancer mortality risk for a 1SD increase of score among 451,256 participants of the EPIC study, by country.

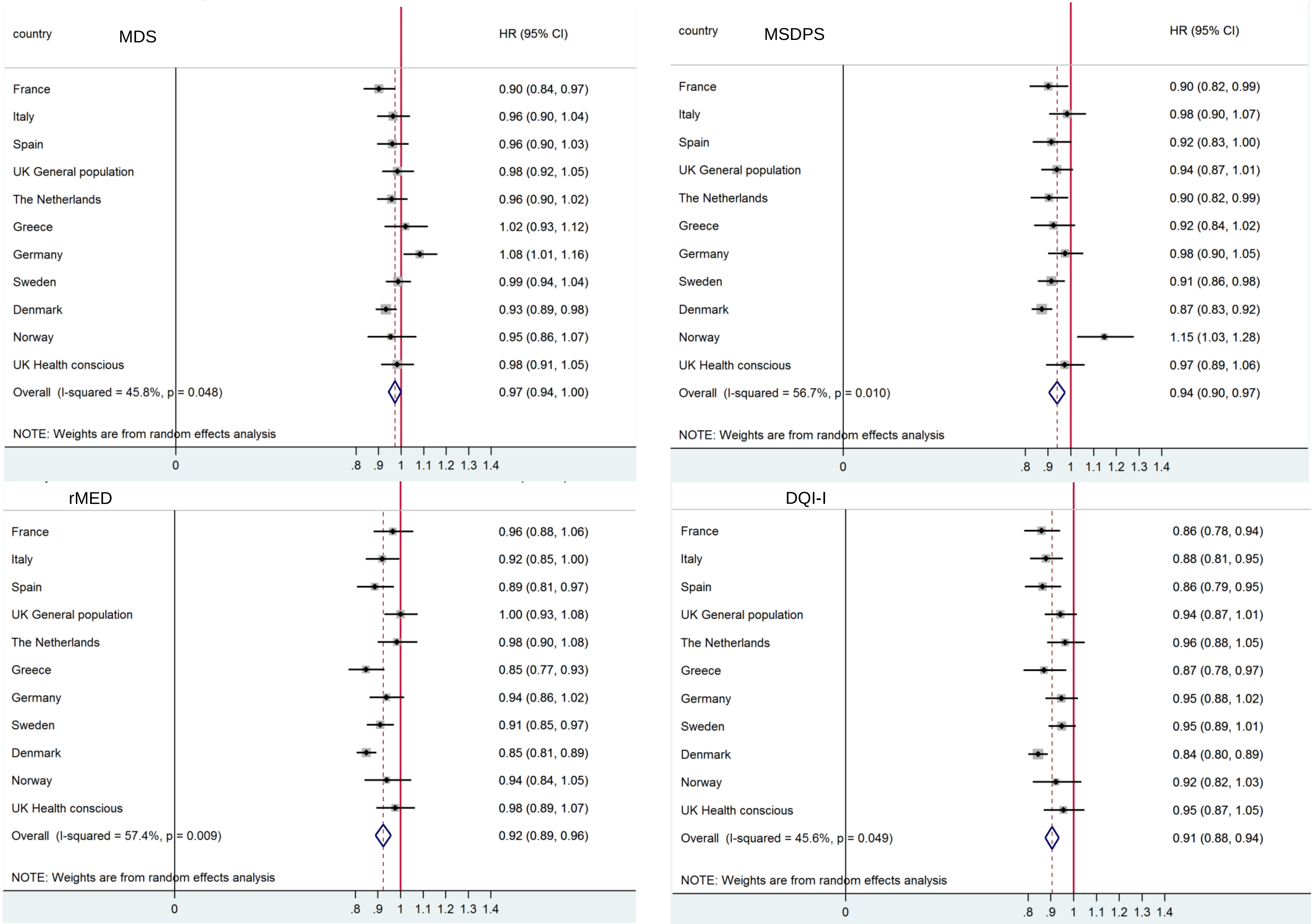

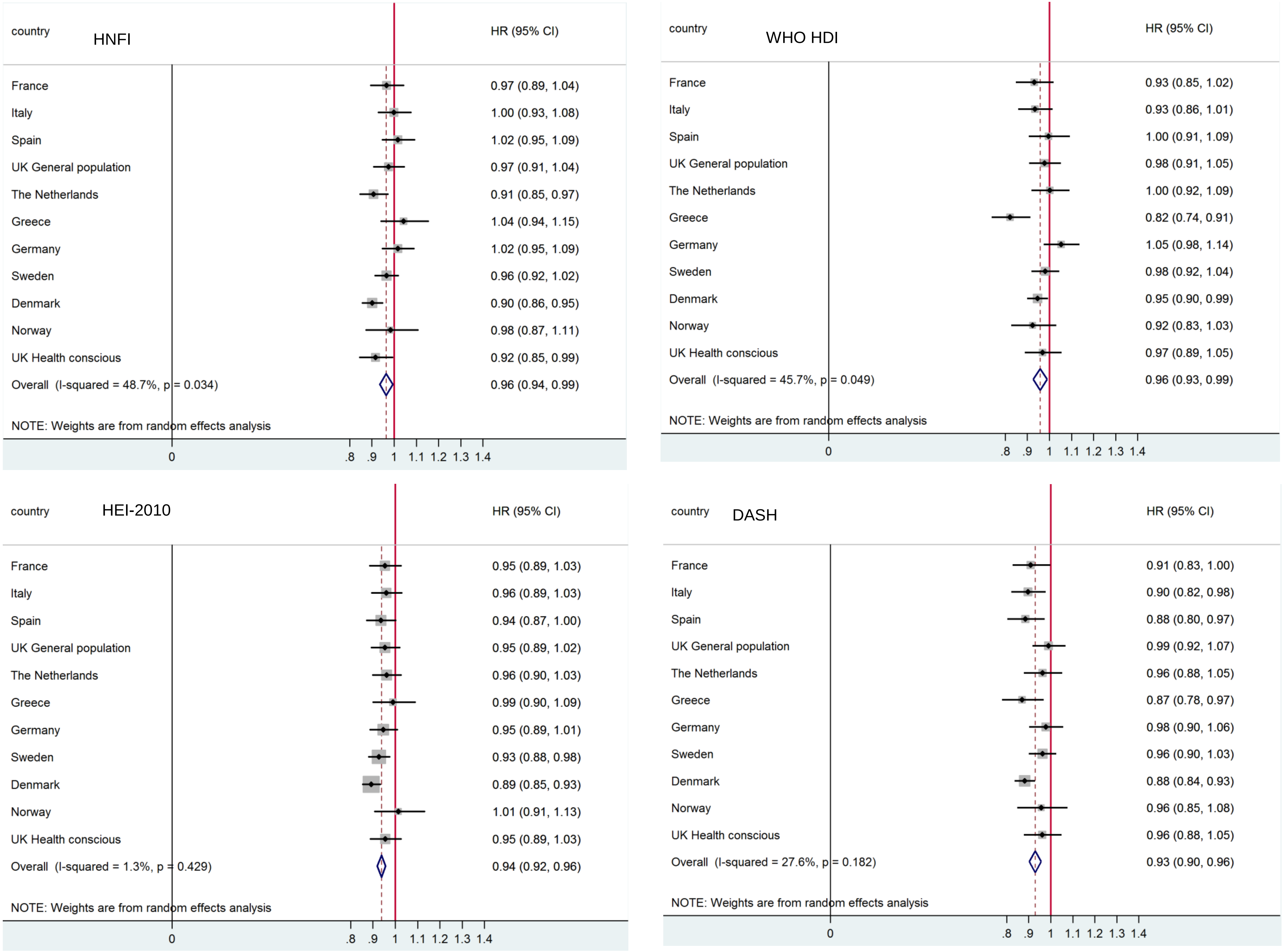
country
HNFI
HR (95% CI)
France 0.97 (0.89, 1.04)
Italy 1.00 (0.93, 1.08)
Spain 1.02 (0.95, 1.09)
UK General population 0.97 (0.91, 1.04)
The Netherlands 0.91 (0.85, 0.97)
Greece 1.04 (0.94, 1.15)
Germany 1.02 (0.95, 1.09)
Sweden 0.96 (0.92, 1.02)
Denmark 0.90 (0.86, 0.95)
Norway 0.98 (0.87, 1.11)
UK Health conscious 0.92 (0.85, 0.99)
Overall (I-squared = 48.7%, p = 0.034) 0.96 (0.94, 0.99)
NOTE: Weights are from random effects analysis
0 .8 .9 1 1.1 1.2 1.3 1.4
country
WHO HDI
HR (95% CI)
France 0.93 (0.85, 1.02)
Italy 0.93 (0.86, 1.01)
Spain 1.00 (0.91, 1.09)
UK General population 0.98 (0.91, 1.05)
The Netherlands 1.00 (0.92, 1.09)
Greece 0.82 (0.74, 0.91)
Germany 1.05 (0.98, 1.14)
Sweden 0.98 (0.92, 1.04)
Denmark 0.95 (0.90, 0.99)
Norway 0.92 (0.83, 1.03)
UK Health conscious 0.97 (0.89, 1.05)
Overall (I-squared = 45.7%, p = 0.049) 0.96 (0.93, 0.99)
NOTE: Weights are from random effects analysis
0 .8 .9 1 1.1 1.2 1.3 1.4
country
HEI-2010
HR (95% CI)
France 0.95 (0.89, 1.03)
Italy 0.96 (0.89, 1.03)
Spain 0.94 (0.87, 1.00)
UK General population 0.95 (0.89, 1.02)
The Netherlands 0.96 (0.90, 1.03)
Greece 0.99 (0.90, 1.09)
Germany 0.95 (0.89, 1.01)
Sweden 0.93 (0.88, 0.98)
Denmark 0.89 (0.85, 0.93)
Norway 1.01 (0.91, 1.13)
UK Health conscious 0.95 (0.89, 1.03)
Overall (I-squared = 1.3%, p = 0.429) 0.94 (0.92, 0.96)
NOTE: Weights are from random effects analysis
0 .8 .9 1 1.1 1.2 1.3 1.4
country
DASH
HR (95% CI)
France 0.91 (0.83, 1.00)
Italy 0.90 (0.82, 0.98)
Spain 0.88 (0.80, 0.97)
UK General population 0.99 (0.92, 1.07)
The Netherlands 0.96 (0.88, 1.05)
Greece 0.87 (0.78, 0.97)
Germany 0.98 (0.90, 1.06)
Sweden 0.96 (0.90, 1.03)
Denmark 0.88 (0.84, 0.93)
Norway 0.96 (0.85, 1.08)
UK Health conscious 0.96 (0.88, 1.05)
Overall (I-squared = 27.6%, p = 0.182) 0.93 (0.90, 0.96)
NOTE: Weights are from random effects analysis
0 .8 .9 1 1.1 1.2 1.3 1.4

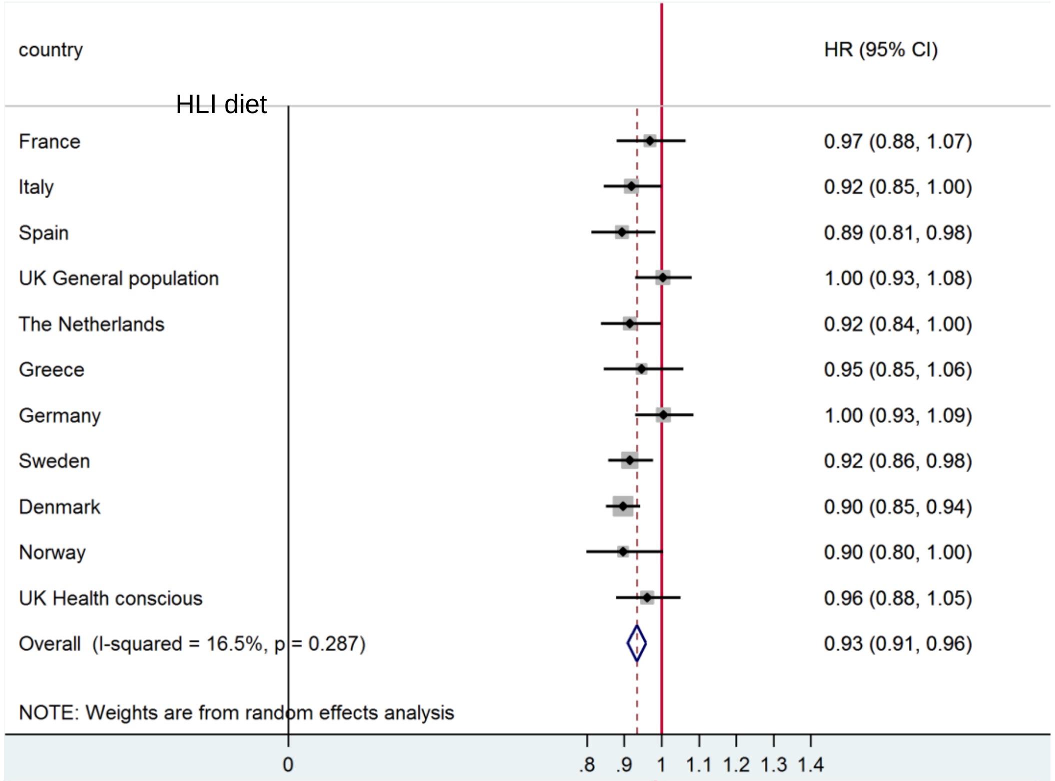
HLI diet
country
HR (95% CI)
France 0.97 (0.88, 1.07)
Italy 0.92 (0.85, 1.00)
Spain 0.89 (0.81, 0.98)
UK General population 1.00 (0.93, 1.08)
The Netherlands 0.92 (0.84, 1.00)
Greece 0.95 (0.85, 1.06)
Germany 1.00 (0.93, 1.09)
Sweden 0.92 (0.86, 0.98)
Denmark 0.90 (0.85, 0.94)
Norway 0.90 (0.80, 1.00)
UK Health conscious 0.96 (0.88, 1.05)
Overall (I-squared = 16.5%, p = 0.287) 0.93 (0.91, 0.96)
NOTE: Weights are from random effects analysis
0
.8
.9
1
1.1
1.2
1.3
1.4

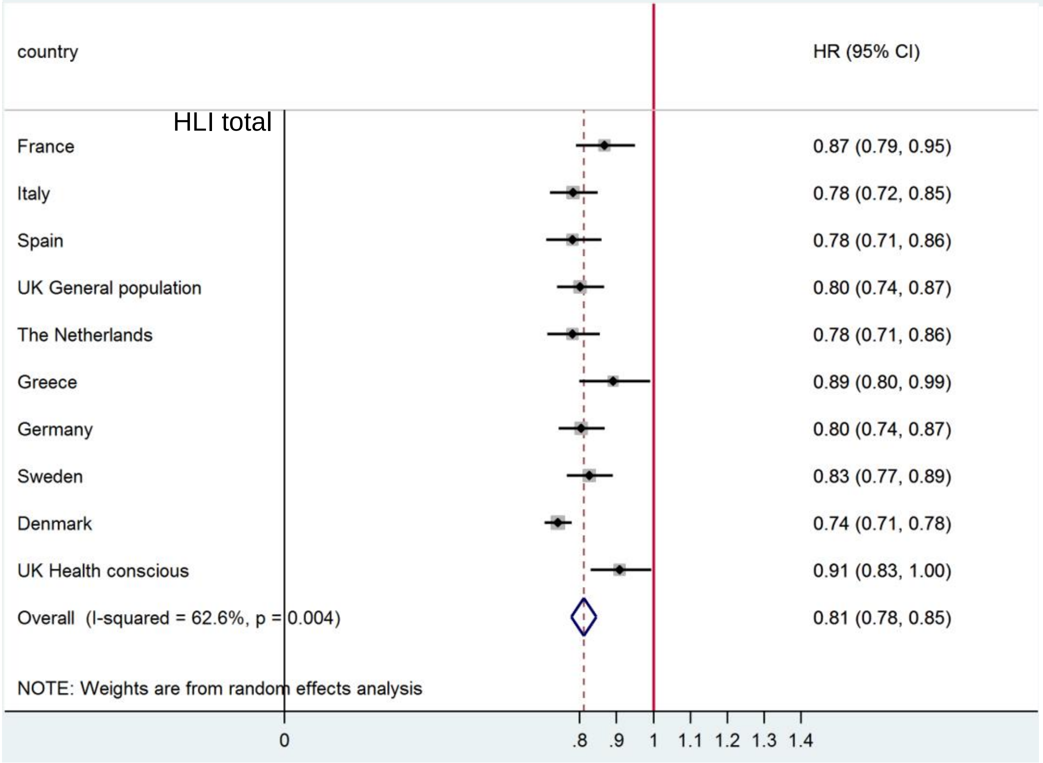
HLI total
country
HR (95% CI)
France 0.87 (0.79, 0.95)
Italy 0.78 (0.72, 0.85)
Spain 0.78 (0.71, 0.86)
UK General population 0.80 (0.74, 0.87)
The Netherlands 0.78 (0.71, 0.86)
Greece 0.89 (0.80, 0.99)
Germany 0.80 (0.74, 0.87)
Sweden 0.83 (0.77, 0.89)
Denmark 0.74 (0.71, 0.78)
UK Health conscious 0.91 (0.83, 1.00)
Overall (I-squared = 62.6%, p = 0.004) 0.81 (0.78, 0.85)
NOTE: Weights are from random effects analysis
0
.8
.9
1
1.1
1.2
1.3
1.4

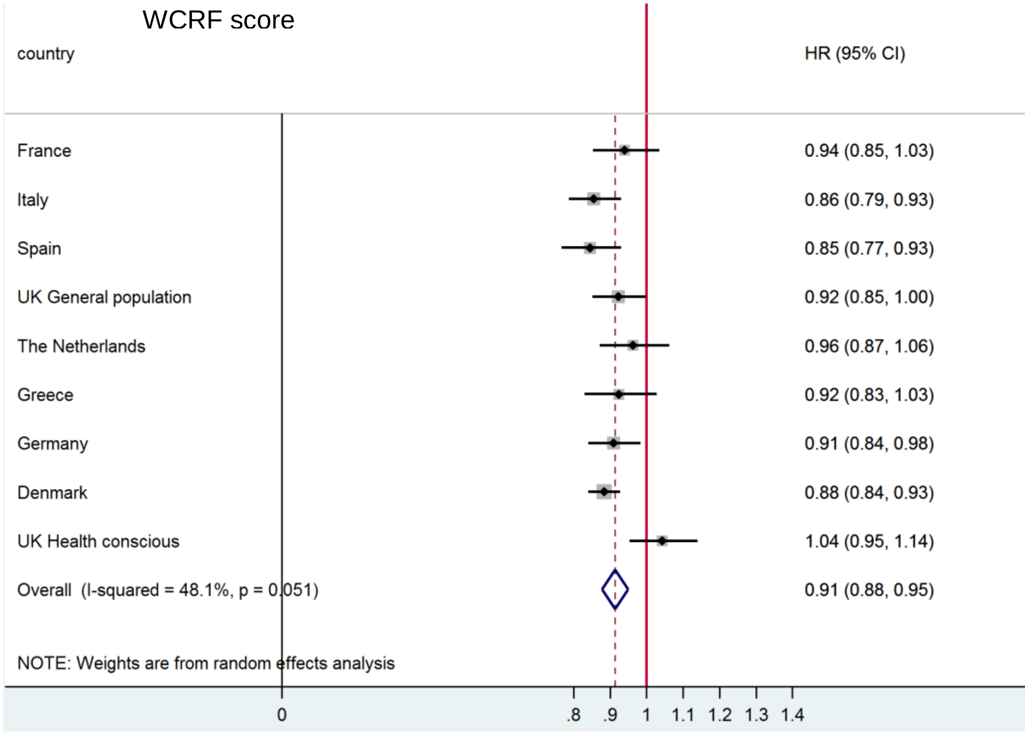
WCRF score
country
HR (95% CI)
France 0.94 (0.85, 1.03)
Italy 0.86 (0.79, 0.93)
Spain 0.85 (0.77, 0.93)
UK General population 0.92 (0.85, 1.00)
The Netherlands 0.96 (0.87, 1.06)
Greece 0.92 (0.83, 1.03)
Germany 0.91 (0.84, 0.98)
Denmark 0.88 (0.84, 0.93)
UK Health conscious 1.04 (0.95, 1.14)
Overall (I-squared = 48.1%, p = 0.051) 0.91 (0.88, 0.95)
NOTE: Weights are from random effects analysis
0
.8
.9
1
1.1
1.2
1.3
1.4
