# Supplementary material for: Diet Quality Scores and Prediction of All-Cause, Cardiovascular and Cancer Mortality in a Pan-European Cohort Study
Source: PLoS One. 2016 Jul 13;11(7):e0159025. doi: 10.1371/journal.pone.0159025 (PMC4943719; doi:10.1371/journal.pone.0159025)

S3a Fig. Discrimination (Harrell's C statistic) of diet/lifestyle quality scores in Model 2 for 10-year risk of all-cause mortality among 451,256 participants to the EPIC study, by country.

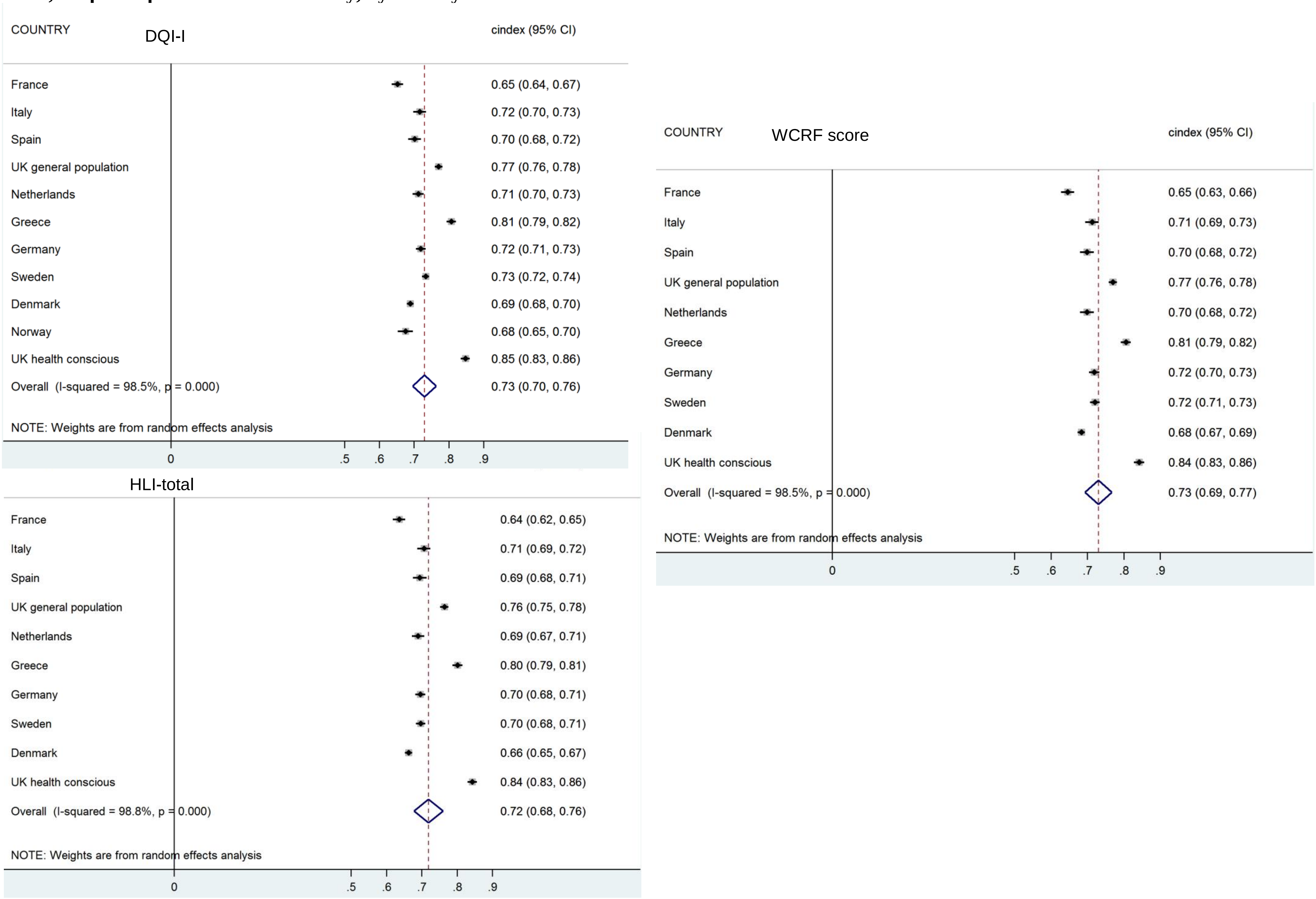

S3b Fig. Discrimination (Harrell's C statistic) of diet/lifestyle quality scores in Model 2 for 10-year risk of CVD mortality among 451,256 participants to the EPIC study, by country.

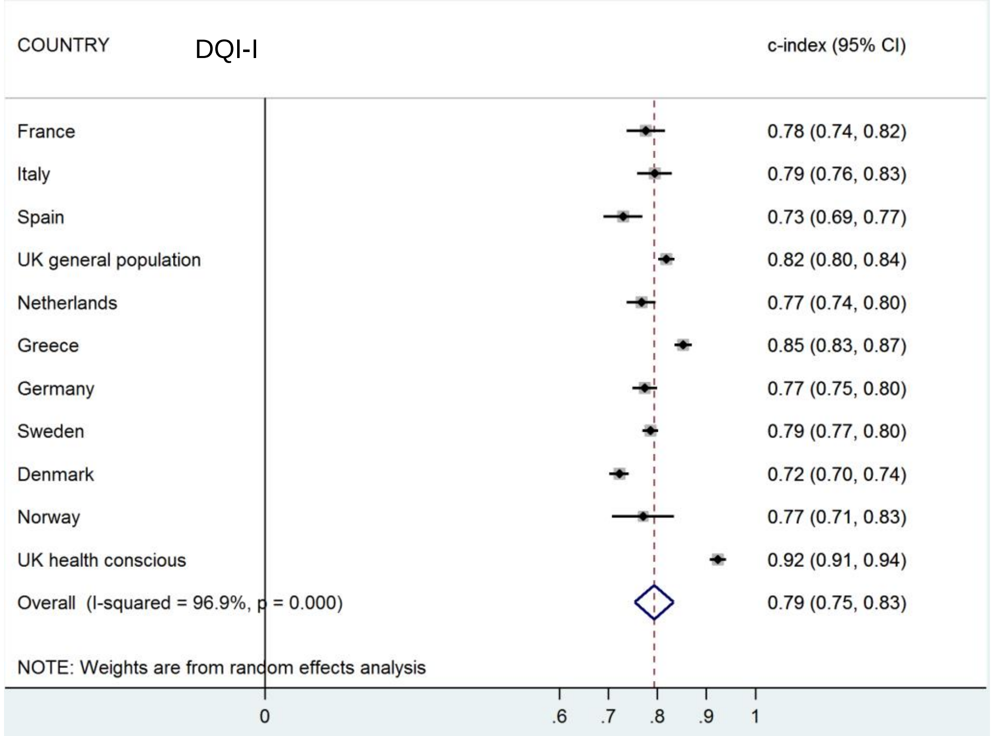

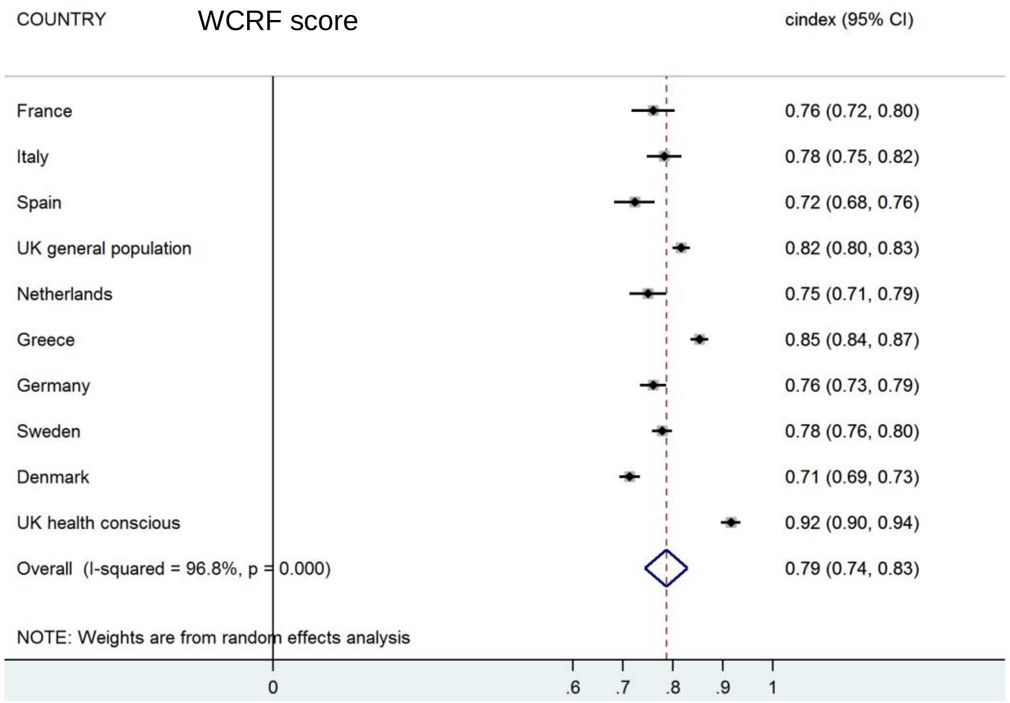

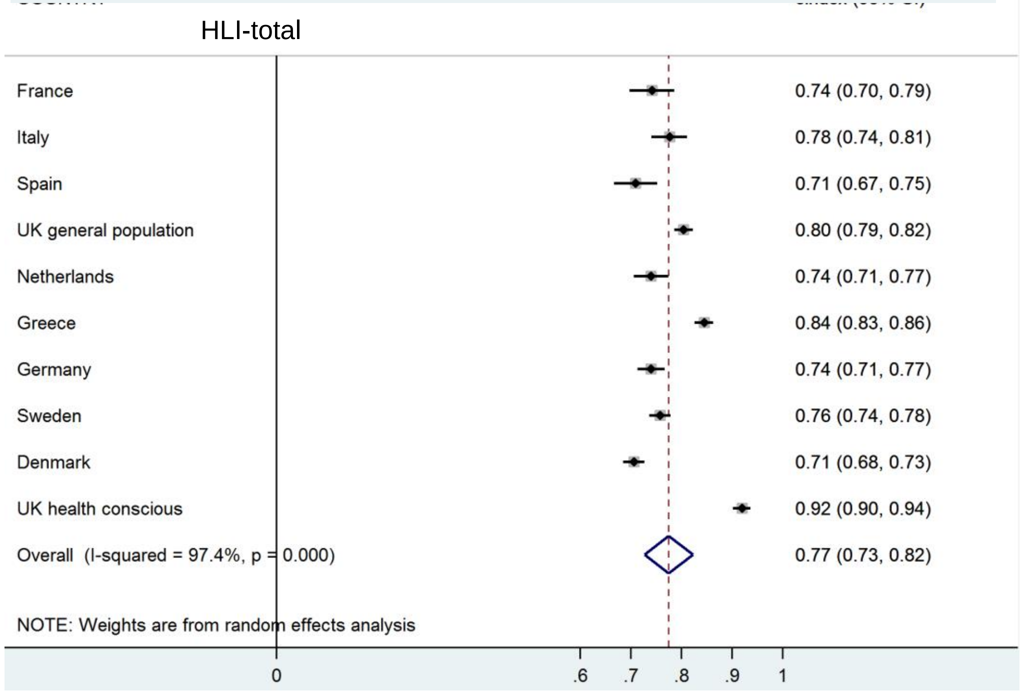

S3c Fig. Discrimination (Harrell's C statistic) of diet/lifestyle quality scores in Model 2 for 10-year risk of cancer mortality among 451,256 participants to the EPIC study, by country

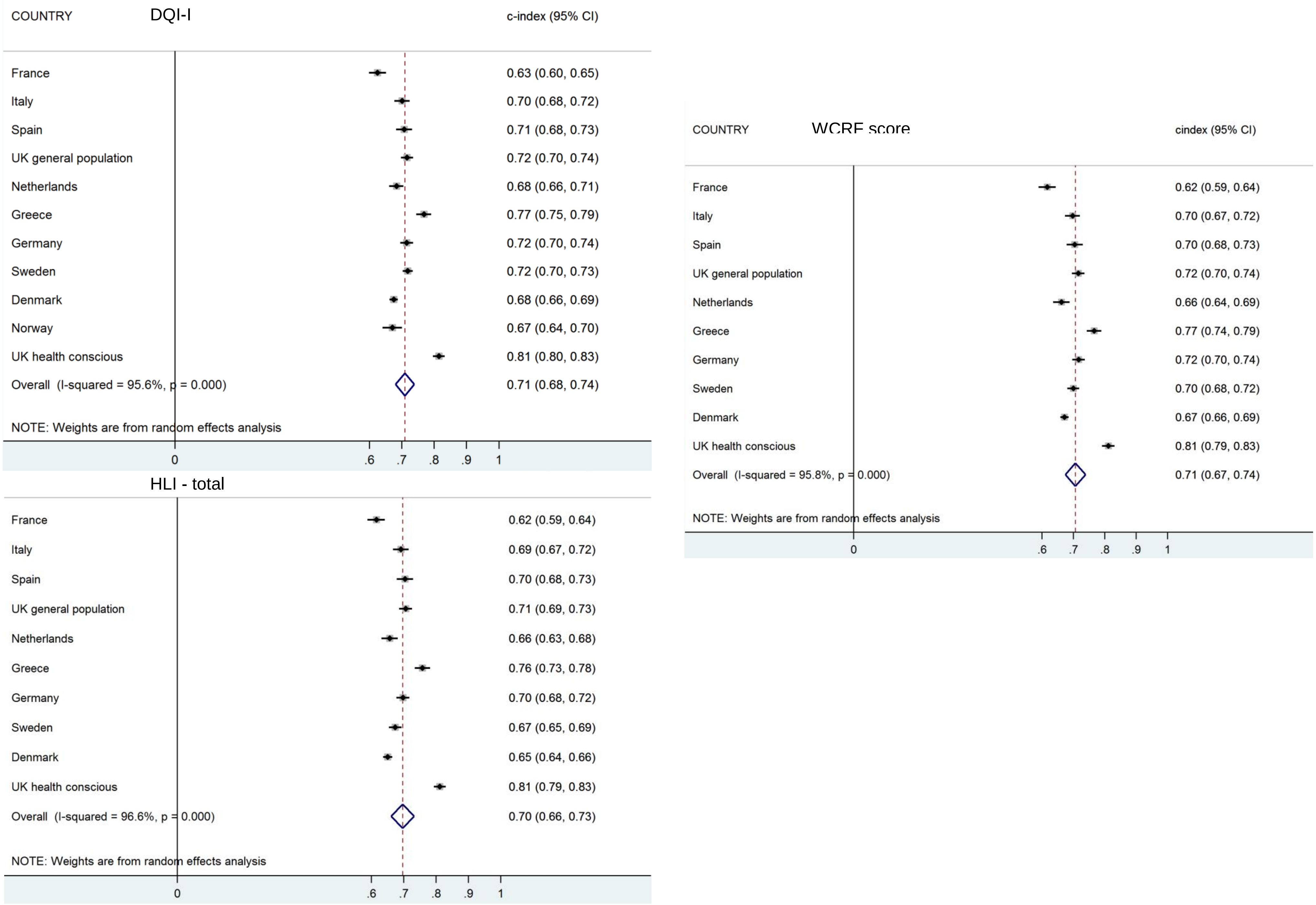

Supplement: S3 Fig — Discrimination (Harrell’s C statistic) of diet/lifestyle quality scores in Model 2 (predictors: age, diet quality score, physical activity, smoking, BMI, educational level, stratified by sex and center) for 10-year risk of all-cause (S3A Fig), CVD (S3B Fig), and cancer (S3C Fig) mortality among 451,256 participants of the EPIC study, by country. (PDF) [file pone.0159025.s003.pdf]
