# Supplementary material for: Diet Quality Scores and Prediction of All-Cause, Cardiovascular and Cancer Mortality in a Pan-European Cohort Study
Source: PLoS One. 2016 Jul 13;11(7):e0159025. doi: 10.1371/journal.pone.0159025 (PMC4943719; doi:10.1371/journal.pone.0159025)

S4a Fig. C statistic of the baseline model [a], Model 1 [b] and Model 2 [c] in 451,256 participants to the EPIC study, by sex.

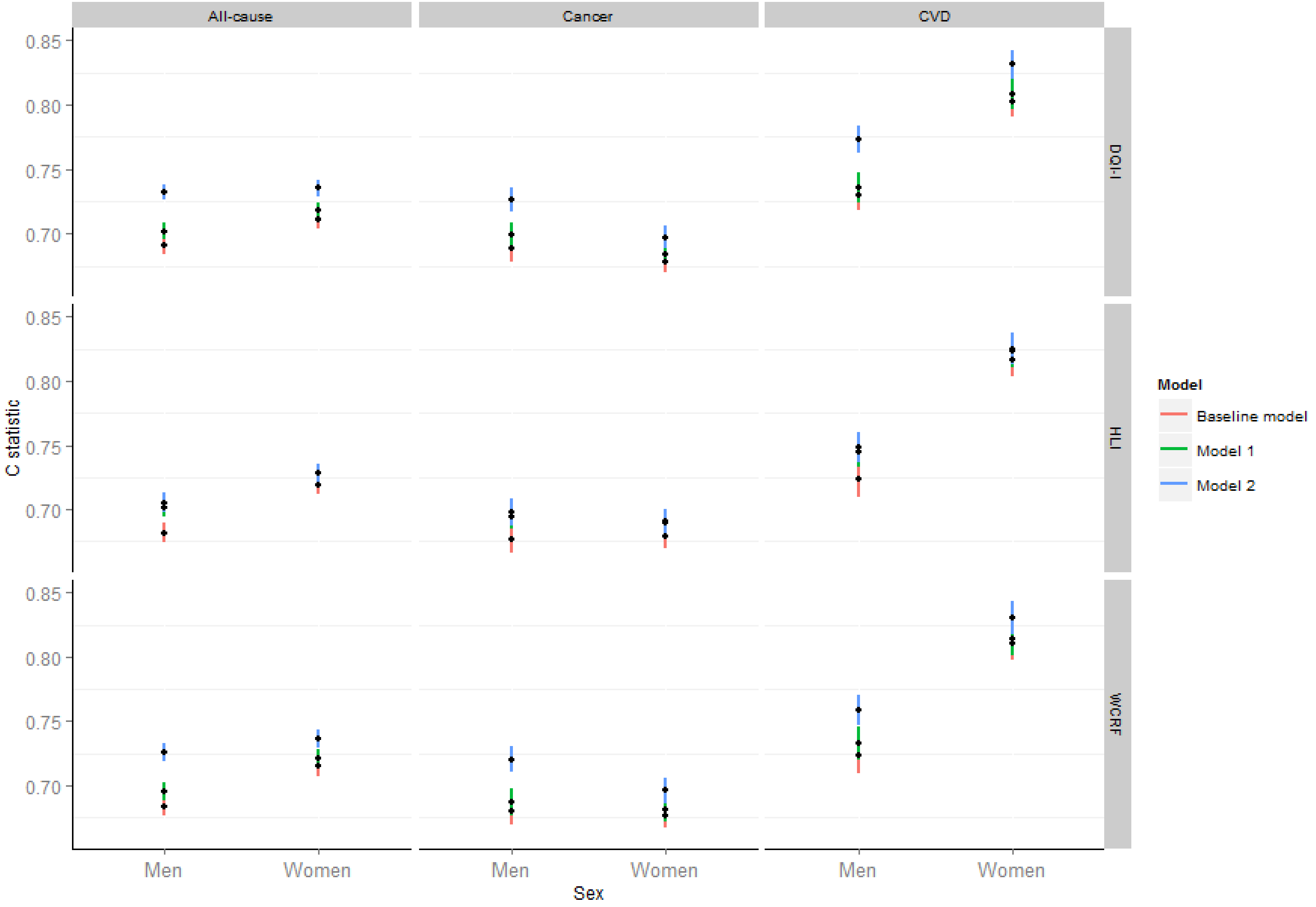

S4b Fig. C statistic of the baseline model [a], Model 1 [b] and Model 2 [c] in 451,256 participants to the EPIC study, by age.

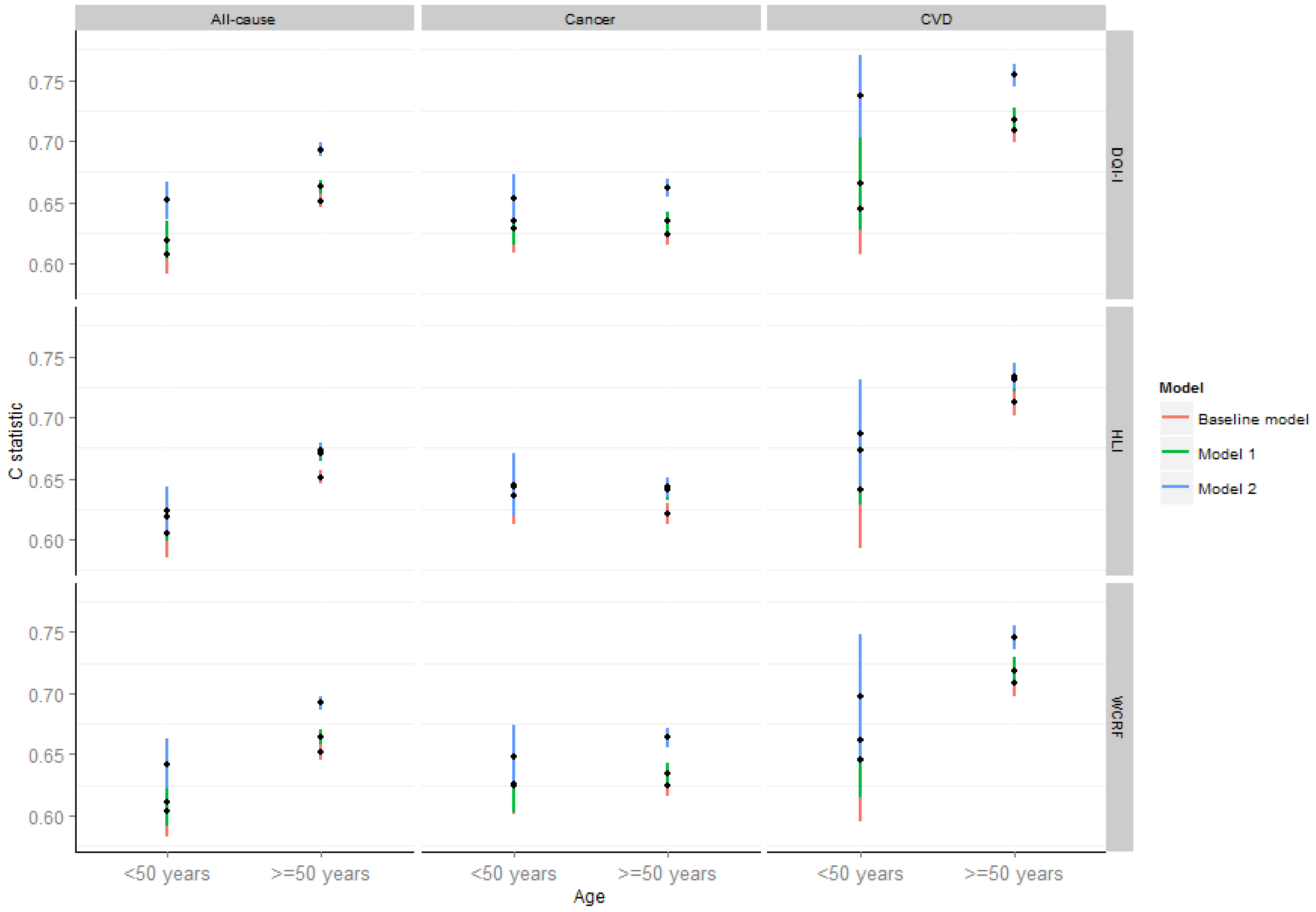

Supplement: S4 Fig — Cstatistic of the baseline model a, Model 1 b and Model 2 c in 451,256 participants to the EPIC study, by sex (S4A Fig) and by age category (S4B Fig). a Baseline model includes only age as a predictor, stratified by sex and center; b Model 1 also includes the dietary score; c Model 2 also includes lifestyle factors: smoking, BMI, physical activity, educational level for DQI-I, smoking and educational level for WCRF, educational level for HLI. (PDF) [file pone.0159025.s004.pdf]
