# Supplementary material for: Diet Quality Scores and Prediction of All-Cause, Cardiovascular and Cancer Mortality in a Pan-European Cohort Study
Source: PLoS One. 2016 Jul 13;11(7):e0159025. doi: 10.1371/journal.pone.0159025 (PMC4943719; doi:10.1371/journal.pone.0159025)
Supplement: S1 File — (PDF) [file pone.0159025.s005.pdf]

S1 File. Details on recruitment of participants to the European Prospective Investigation into Cancer and Nutrition, selection of analysis sample and dietary data management

521,448 participants aged 25-70 years were recruited between 1992 and 2000, from 23 study centres in 10 European countries: Denmark (Aarhus and Copenhagen); France; Germany (Heidelberg and Potsdam); Greece; Italy (Florence, Naples, Ragusa, Turin, and Varese); the Netherlands (Bilthoven and Utrecht); Norway (Tromsø); Spain (Asturias, Granada, Murcia, Navarra, and San Sebastian); Sweden (Malmo and Umea); and the United Kingdom (UK; Cambridge and Oxford).

Participants were recruited from the general population of their respective countries, with the following exceptions: the French cohort were teacher health insurance programme members; the Italian and Spanish cohorts included members of blood donor associations and the general population; the Utrecht (the Netherlands) and Florence (Italy) cohorts comprised women from mammographic screening programs; the Oxford (UK) cohort included health-conscious participants; finally, only women participated in the cohorts of France, Norway, Naples (Italy) and Utrecht (the Netherlands). Written informed consent was provided by all study participants.

Among the participants with complete dietary data at baseline (n=514,493), we excluded participants with missing follow-up information (n=2,712); participants in the highest and lowest 1% of the distribution for the ratio between energy intake to estimated energy requirement (n=10,132); participants who had cancer at baseline (n=23,243); who self-reported a history of heart disease (n=7,007), angina (n=8,335), stroke (n=4,156), or diabetes (n=13,844) at baseline; pregnant women (n=581), leaving 451,256 participants (130,370 men and 320,886 women).

To compute WCRF/AICR score, further exclusion criteria were applied: participants from Umea (Sweden) and from Norway, for which physical activity was not collected with sufficient detail and women with no information on breastfeeding or duration of breastfeeding (including all female participants from Bilthoven, Netherlands). This specific subsample comprised 363,207 participants.

To compute the Healthy Lifestyle Index, we excluded participants with missing data for physical activity (see above) and smoking status, leaving 376,553 subjects in the subsample.

Vital status, causes, and dates of death were obtained from record linkages with cancer registries, boards of health, and death indexes (Denmark, Italy, the Netherlands, Norway, Spain, Sweden, and the United Kingdom). For Germany, Greece, and France participants were actively followed-up.

Dietary intake assessment

Dietary intake was assessed by a number of different instruments that had been developed and validated previously in a series of studies within the various source populations participating in EPIC. Following the results of the methodological studies and taking into account the local context, three dietary assessment methods were adopted:

1. Extensive self-administrated quantitative dietary questionnaires, containing up to 260 food items and estimating individual average portions systematically, were used in northern Italy,

The Netherlands, Germany and Greece (where dietary questionnaires were interviewer-administered). Questionnaires, similar in content to the self-administered quantitative dietary questionnaires but structured by meals, were used in Spain, France and Ragusa (south Italy). To increase compliance, the centres in Spain and Ragusa performed a face-to-face dietary interview using a computerised dietary program, whereas the dietary questionnaire was self-reported in France.

2. Semi-quantitative food-frequency questionnaires (with the same standard portion(s) assigned to all subjects) were used in Denmark, Norway, Naples in Italy and Umea in Sweden.

3. Combined dietary methods were used in the UK and Malmo¨ (Sweden). The two British centres used both a semi-quantitative food-frequency questionnaire and a 7-day record, whereas a method combining a short non-quantitative food-frequency questionnaire with a 14-day record on hot meals (lunches and dinners) was developed in Malmo.
