# Supplementary material for: Diet Quality Scores and Prediction of All-Cause, Cardiovascular and Cancer Mortality in a Pan-European Cohort Study
Source: PLoS One. 2016 Jul 13;11(7):e0159025. doi: 10.1371/journal.pone.0159025 (PMC4943719; doi:10.1371/journal.pone.0159025)
Supplement: S1 Table — + indicates positive weighting (encourages consumption);—indicates negative weighting (limits consumption). (PDF) [file pone.0159025.s006.pdf]

S1 Table. Description of the food and nutrient components used for the calculation of the 11 dietary scores

	MDS	rMED	MSDPS	HEI2010	DQI-I	DASH	HNFI	WHO HDI	HLI-diet	WCRF
**Food**										
Alcohol	+	+	+ (wine)							-
Dairy	-	-	-			+ (low-fat)				
Egg			-							
Empty calorie food				-	-					-
Fish	+	+	+	+			+		+ (fatty)	
Fruit	+	+	+	+	+	+	+ (apples pears)	+	+	+
Grains	+	+			+					
Refined				-						
Whole			+	+		+	+ (rye bread)			
Legumes	+	+	+	+		+				
Nuts			+			+				
Olive oil		+	+							
Meat			-			-				
Poultry			+							
Red and processed	-	-								-
Potatoes			-							
Sugar sweetened beverages sweet products			-			-				-
Total protein foods				+						
Vegetables (excluding potatoes)	+	+	+	+	+	+	+ (root)	+	+	+
Variety					+					
**Nutrients**										
Dietary fibre					+			+	+	+
Protein					+			+		

Fat			-			
PUFA			+		+	+
MUFA	+		+			
SFA	-		-		-	-
Cholesterol			-		-	
Trans-fat						-
Free sugars					-	
Glycemic load						-
Iron			+			
Calcium			+			
Potassium						
Magnesium						
Vitamin C			+			
Folate						+
Sodium		-	-	-		

+ indicates positive weighting (encourages consumption)

- indicates negative weighting (limits consumption)

Abbreviations: MDS, Mediterranean Diet Scale; rMED, relative Mediterranean diet score; MSDPS, Mediterranean Style Dietary Pattern Score; DQI-I, Diet Quality Index – International; HNFI, Healthy Nordic Food Index; HEI-2010, Healthy Eating Index 2010; WHO HDI, World Health Organization Healthy Diet Index; DASH, Dietary Approach to Stop Hypertension; HLI, Healthy Lifestyle Index; HLI-diet, diet component of the HLI; WCRF, World Cancer Research Fund / American Institute for Cancer Research
