# Supplementary material for: Diet Quality Scores and Prediction of All-Cause, Cardiovascular and Cancer Mortality in a Pan-European Cohort Study
Source: PLoS One. 2016 Jul 13;11(7):e0159025. doi: 10.1371/journal.pone.0159025 (PMC4943719; doi:10.1371/journal.pone.0159025)
Supplement: S2 Table — a Model including the following predictors: age at baseline, Physical activity (Cambridge index), smoking status (3 categories) and educational level, unless otherwise stated. Stratified by study center and sex. b HR for the increase of 1 SD of score. c p-value for linear trend across quartiles. d Model only including HLI, age and educational level because BMI, physical activity, smoking are components of the Healthy Lifestyle Index, n = 376,553. e Model only including WCRF score, smoking and educational level as BMI and physical activity are components of the WCRF score, n = 363,207. (PDF) [file pone.0159025.s007.pdf]

S2 Table. Multivariate hazard ratios and C-statistic (Model 2[a]) for 10-year mortality risk due to obesity-related cancer for a 1SD increase of score among 451,256 participants of the EPIC study

	HR	HR (95% CI)	HR (95% CI)	HR (95% CI)	HR (95% CI)	P trend [c]	C-statistic
	Q1	Q2	Q3	Q4	Continuous [b]		
MDS	1 (ref)	0.91(0.82- 1.00)	0.91(0.82- 1.00)	0.87(0.79- 0.97)	0.95 (0.92 - 0.99)	0.51	0.690 (0.679 - 0.701)
rMED	1 (ref)	0.96(0.87- 1.06)	0.95(0.86- 1.05)	0.87(0.78- 0.96)	0.94 (0.90 - 0.97)	0.06	0.690 (0.679 - 0.701)
MSDPS	1 (ref)	0.96(0.87- 1.07)	0.99(0.89- 1.09)	0.85(0.76- 0.94)	0.95 (0.91 - 0.99)	0.02	0.690 (0.679 - 0.700)
DQI-I	1 (ref)	0.89(0.80- 0.98)	0.84(0.76- 0.94)	0.83(0.75- 0.92)	0.93 (0.90 - 0.97)	0.24	0.690 (0.679 - 0.701)
HNDS	1 (ref)	0.97(0.87- 1.07)	0.87(0.78- 0.97)	0.85(0.76- 0.95)	0.94 (0.90 - 0.98)	0.02	0.690 (0.679 - 0.701)
HEI 2010	1 (ref)	0.92(0.83- 1.02)	0.85(0.77- 0.95)	0.90(0.81- 0.99)	0.94 (0.90 - 0.97)	0.57	0.690 (0.679 - 0.700)
WHO HDI	1 (ref)	0.99(0.89- 1.10)	0.93(0.83- 1.04)	0.94(0.84- 1.05)	0.98 (0.94 - 1.02)	0.33	0.689 (0.678 - 0.700)
DASH	1 (ref)	0.97(0.87- 1.07)	0.94(0.84- 1.04)	0.93(0.84- 1.04)	0.95 (0.92 - 0.99)	0.51	0.689 (0.678 - 0.700)
HLI – diet	1 (ref)	0.90(0.81- 0.99)	0.87(0.78- 0.96)	0.88(0.79- 0.98)	0.95 (0.91 - 0.99)	0.74	0.690 (0.679 - 0.700)
HLI - total [d]	1 (ref)	0.81(0.73- 0.91)	0.76(0.68- 0.85)	0.62(0.55- 0.70)	0.83 (0.79 - 0.86)	<0.0001	0.682 (0.670 - 0.694)
WCRF [e]	1 (ref)	0.85(0.76- 0.94)	0.76(0.68- 0.85)	0.72(0.64- 0.80)	0.88 (0.84 - 0.91)	0.004	0.682 (0.671 - 0.694)

Abbreviations: MDS, Mediterranean Diet Scale; rMED, relative Mediterranean diet score; MSDPS, Mediterranean Style Dietary Pattern Score; DQI-I, Diet Quality Index – International; HNFI, Healthy Nordic Food Index; HEI-2010, Healthy Eating Index 2010; WHO HDI, World Health Organization Healthy Diet Index; DASH, Dietary Approach to Stop Hypertension; HLI, Healthy Lifestyle Index; HLI-diet, diet component of the HLI; WCRF, World Cancer Research Fund / American Institute for Cancer Research

[a] Model including the following predictors: age at baseline, Physical activity (Cambridge index), smoking status (3 categories) and educational level, unless otherwise stated. Stratified by study center and sex.

[b] HR for the increase of 1 SD of score

[c] p-value for linear trend across quartiles

[d] Model only including HLI, age and educational level because BMI, physical activity, smoking are components of the Healthy Lifestyle Index, n=376,553

[e] Model only including WCRF score, smoking and educational level as BMI and physical activity are components of the WCRF score, n=363,207
