# Supplementary material for: Diet Quality Scores and Prediction of All-Cause, Cardiovascular and Cancer Mortality in a Pan-European Cohort Study
Source: PLoS One. 2016 Jul 13;11(7):e0159025. doi: 10.1371/journal.pone.0159025 (PMC4943719; doi:10.1371/journal.pone.0159025)
Supplement: S3 Table — a Model including the following predictors: dietary score, energy intake and age at baseline, BMI (continuous), Physical activity (Cambridge index), smoking status (3 categories) and educational level, unless otherwise stated. Stratified by sex and study center. b HR for the increase of 1 SD of score. c p-value for linear trend across quartiles. d Model only including HLI, age, energy intake and educational level because BMI, physical activity, smoking are components of the Healthy Lifestyle Index, n = 376,553. e Model only including WCRF score, age, energy intake, smoking and educational level as BMI and physical activity are components of the WCRF score, n = 363,207. (PDF) [file pone.0159025.s008.pdf]

S3 Table. Multivariate hazard ratios adjusted for total energy intake a for 10-year mortality risk by quartile of score and for a 1SD increase of score among 451,256 participants of the EPIC study

	HR	HR (95% CI)	HR (95% CI)	HR (95% CI)	HR (95% CI)	P-trend [c]
**All-cause mortality**	**Q1**	**Q2**	**Q3**	**Q4**	**Continuous [b]**	
MDS	1 (ref)	0.89 (0.86- 0.93)	0.84 (0.80- 0.87)	0.78 (0.74- 0.82)	0.91 (0.89 - 0.92)	<0.0001
rMED	1 (ref)	0.87 (0.84- 0.91)	0.81 (0.77- 0.85)	0.77 (0.74- 0.81)	0.89 (0.88 - 0.91)	<0.0001
MSDPS	1 (ref)	0.92 (0.88- 0.96)	0.87 (0.84- 0.91)	0.79 (0.76- 0.83)	0.91 (0.90 - 0.93)	<0.0001
DQI-I	1 (ref)	0.88 (0.84- 0.92)	0.79 (0.76- 0.83)	0.74 (0.71- 0.77)	0.89 (0.87 - 0.90)	<0.0001
HNFI	1 (ref)	0.93 (0.89- 0.97)	0.85 (0.82- 0.89)	0.80 (0.77- 0.84)	0.92 (0.91 - 0.94)	<0.0001
HEI 2010	1 (ref)	0.89 (0.85- 0.93)	0.84 (0.80- 0.88)	0.81 (0.78- 0.85)	0.91 (0.90 - 0.93)	0.0001
WHO HDI	1 (ref)	0.93 (0.89- 0.98)	0.88 (0.84- 0.92)	0.87 (0.83- 0.92)	0.95 (0.94 - 0.97)	0.005
DASH	1 (ref)	0.90 (0.86- 0.94)	0.84 (0.80- 0.87)	0.80 (0.76- 0.83)	0.91 (0.89 - 0.92)	<0.0001
HLI – diet	1 (ref)	0.91 (0.87- 0.95)	0.85 (0.81- 0.89)	0.82 (0.78- 0.85)	0.93 (0.91 - 0.94)	<0.0001
HLI - total [d]	1 (ref)	0.75 (0.72- 0.78)	0.65 (0.62- 0.68)	0.55 (0.52- 0.57)	0.79 (0.77 - 0.80)	<0.0001
WCRF [e]	1 (ref)	0.87 (0.83- 0.91)	0.78 (0.74- 0.82)	0.70 (0.67- 0.74)	0.86 (0.85 - 0.88)	<0.0001
**CVD mortality**						
MDS	1 (ref)	0.81 (0.74- 0.88)	0.80 (0.73- 0.87)	0.80 (0.72- 0.88)	0.91 (0.88 - 0.94)	0.76
rMED	1 (ref)	0.88 (0.81- 0.96)	0.80 (0.73- 0.88)	0.77 (0.70- 0.85)	0.89 (0.86 - 0.92)	0.01
MSDPS	1 (ref)	0.93 (0.85- 1.01)	0.90 (0.83- 0.99)	0.81 (0.73- 0.89)	0.92 (0.89 - 0.95)	0.004
DQI-I	1 (ref)	0.90 (0.83- 0.98)	0.80 (0.73- 0.87)	0.75 (0.68- 0.83)	0.89 (0.87 - 0.92)	0.0002
HNFI	1 (ref)	0.91 (0.83- 0.99)	0.88 (0.80- 0.96)	0.81 (0.73- 0.89)	0.93 (0.90 - 0.96)	0.02
HEI 2010	1 (ref)	0.89 (0.82- 0.97)	0.85 (0.78- 0.93)	0.82 (0.75- 0.90)	0.93 (0.90 - 0.96)	0.10
WHO HDI	1 (ref)	0.95 (0.87- 1.04)	0.88 (0.80- 0.96)	0.84 (0.76- 0.92)	0.93 (0.90 - 0.96)	0.01
DASH	1 (ref)	0.86 (0.79- 0.94)	0.80 (0.74- 0.88)	0.76 (0.69- 0.84)	0.89 (0.86 - 0.92)	0.01
HLI – diet	1 (ref)	0.93 (0.85- 1.01)	0.84 (0.77- 0.92)	0.78 (0.71- 0.86)	0.91 (0.88 - 0.94)	0.0003
HLI - total [d]	1 (ref)	0.72 (0.66- 0.78)	0.59 (0.54- 0.65)	0.47 (0.42- 0.52)	0.74 (0.71 - 0.77)	<0.0001
WCRF [e]	1 (ref)	0.81 (0.74- 0.88)	0.76 (0.69- 0.83)	0.63 (0.57- 0.70)	0.83 (0.81 - 0.86)	<0.0001
**Cancer mortality**						
MDS	1 (ref)	0.95 (0.89- 1.01)	0.90 (0.85- 0.96)	0.82 (0.76- 0.87)	0.93 (0.90 - 0.95)	<0.0001
rMED	1 (ref)	0.92 (0.86- 0.97)	0.86 (0.81- 0.92)	0.82 (0.77- 0.88)	0.92 (0.90 - 0.94)	0.002
MSDPS	1 (ref)	0.96 (0.90- 1.02)	0.92 (0.87- 0.98)	0.83 (0.78- 0.89)	0.94 (0.91 - 0.96)	<0.0001
DQI-I	1 (ref)	0.87 (0.82- 0.93)	0.82 (0.77- 0.87)	0.77 (0.72- 0.82)	0.91 (0.89 - 0.93)	0.0002
HNFI	1 (ref)	0.98 (0.92- 1.04)	0.91 (0.85- 0.97)	0.84 (0.79- 0.90)	0.94 (0.92 - 0.96)	<0.0001
HEI 2010	1 (ref)	0.94 (0.88- 1.00)	0.86 (0.81- 0.91)	0.86 (0.81- 0.92)	0.92 (0.90 - 0.94)	0.02
WHO HDI	1 (ref)	0.95 (0.89- 1.02)	0.90 (0.84- 0.96)	0.91 (0.85- 0.97)	0.96 (0.94 - 0.99)	0.12
DASH	1 (ref)	0.94 (0.88- 1.00)	0.93 (0.87- 0.99)	0.85 (0.79- 0.91)	0.93 (0.91 - 0.96)	0.005
HLI – diet	1 (ref)	0.91 (0.85- 0.96)	0.88 (0.82- 0.93)	0.85 (0.79- 0.91)	0.94 (0.92 - 0.96)	0.06
HLI - total [d]	1 (ref)	0.75 (0.70- 0.80)	0.67 (0.63- 0.72)	0.56 (0.52- 0.60)	0.80 (0.78 - 0.82)	<0.0001
WCRF [e]	1 (ref)	0.88 (0.82- 0.94)	0.81 (0.76- 0.87)	0.78 (0.72- 0.83)	0.90 (0.88 - 0.92)	0.0004

Abbreviations: MDS, Mediterranean Diet Scale; rMED, relative Mediterranean diet score; MSDPS, Mediterranean Style Dietary Pattern Score; DQI-I, Diet Quality Index – International; HNFI, Healthy Nordic Food Index; HEI-2010, Healthy Eating Index 2010; WHO HDI, World Health Organization Healthy Diet Index; DASH, Dietary Approaches to Stop Hypertension; HLI, Healthy Lifestyle Index; HLI-diet, diet component of the HLI; WCRF, World Cancer Research Fund / American Institute for Cancer Research; Q, quartile of diet quality score

[a] Model including the following predictors: dietary score, energy intake and age at baseline, BMI (continuous), Physical activity (Cambridge index), smoking status (3 categories) and educational level, unless otherwise stated. Stratified by sex and study centre.

[b] HR for the increase of 1 SD of score

[c] p-value for linear trend across quartiles

[d] Model only including HLI, age, energy intake and educational level because BMI, physical activity, smoking are components of the Healthy Lifestyle Index, n=376,553

[e] Model only including WCRF score, age, energy intake, smoking and educational level as BMI and physical activity are components of the WCRF score, n=363,207
