# Supplementary material for: Diet Quality Scores and Prediction of All-Cause, Cardiovascular and Cancer Mortality in a Pan-European Cohort Study
Source: PLoS One. 2016 Jul 13;11(7):e0159025. doi: 10.1371/journal.pone.0159025 (PMC4943719; doi:10.1371/journal.pone.0159025)
Supplement: S4 Table — a Baseline model includes only age as a predictor, stratified by sex and center; b Model 1 = baseline + dietary score; c Model 2 = Model 1+ lifestyle factors: smoking, BMI, physical activity, educational level unless otherwise stated. d Model 2 = Model 1 + educational level because BMI, physical activity, smoking are components of the Healthy Lifestyle Index, n = 376,553. e Model 2 = Model 1 + smoking and educational level as BMI and physical activity are components of the WCRF score, n = 363,207. (PDF) [file pone.0159025.s009.pdf]

S4 Table. C statistic of the baseline model [a], Model 1 [b] and Model 2 [c] for the prediction of 10-year mortality risk in 451,256 participants to the EPIC study

Score	Baseline model [a]	Model 1 [b]	Difference Model 1 - baseline	Model 2 [c]	Difference Model 2 - Baseline
**All cause**					
MDS	0.704 (0.699-0.708)	0.709 (0.704-0.714)	0.005	0.733 (0.729-0.738)	0.029
rMED	0.704 (0.699-0.708)	0.711 (0.707-0.716)	0.007	0.734 (0.729-0.739)	0.030
MSDPS	0.704 (0.699-0.708)	0.709 (0.704-0.713)	0.005	0.733 (0.728-0.738)	0.029
DQI	0.704 (0.699-0.708)	0.712 (0.707-0.716)	0.008	0.734 (0.729-0.738)	0.030
HNFI	0.704 (0.699-0.708)	0.708 (0.703-0.712)	0.004	0.733 (0.728-0.737)	0.029
HEI-2010	0.704 (0.699-0.708)	0.709 (0.705-0.714)	0.005	0.733 (0.729-0.738)	0.029
WHOHDI	0.704 (0.699-0.708)	0.706 (0.701-0.710)	0.002	0.732 (0.728-0.737)	0.028
DASH	0.704 (0.699-0.708)	0.710 (0.705-0.714)	0.006	0.733 (0.728-0.738)	0.029
HLI - diet	0.704 (0.699-0.708)	0.708 (0.703-0.713)	0.004	0.733 (0.728-0.737)	0.029
HLI - total [d]	0.705 (0.700-0.710)	0.718 (0.713-0.723)	0.013	0.720 (0.715-0.725)	0.015
WCRF [e]	0.702 (0.697-0.708)	0.711 (0.706-0.716)	0.008	0.732 (0.727-0.737)	0.029
**CVD**					
MDS	0.77 (0.761-0.779)	0.774 (0.765-0.783)	0.004	0.804 (0.796-0.812)	0.034
rMED	0.77 (0.761-0.779)	0.776 (0.767-0.785)	0.006	0.805 (0.797-0.813)	0.035
MSDPS	0.77 (0.761-0.779)	0.774 (0.765-0.783)	0.004	0.804 (0.796-0.812)	0.034
DQI	0.77 (0.761-0.779)	0.776 (0.767-0.785)	0.006	0.805 (0.797-0.813)	0.035
HNFI	0.77 (0.761-0.779)	0.773 (0.764-0.782)	0.003	0.804 (0.796-0.812)	0.034
HEI-2010	0.77 (0.761-0.779)	0.774 (0.765-0.783)	0.004	0.804 (0.796-0.812)	0.034
WHOHDI	0.77 (0.761-0.779)	0.773 (0.764-0.782)	0.003	0.804 (0.796-0.812)	0.034
DASH	0.77 (0.761-0.779)	0.776 (0.767-0.785)	0.006	0.805 (0.796-0.813)	0.035
HLI - diet	0.77 (0.761-0.779)	0.775 (0.766-0.783)	0.005	0.804 (0.796-0.812)	0.034
HLI - total [d]	0.776 (0.766-0.786)	0.789 (0.78-0.799)	0.013	0.792 (0.782-0.801)	0.015
WCRF [e]	0.769 (0.759-0.779)	0.776 (0.766-0.785)	0.006	0.796 (0.787-0.805)	0.027
**Cancer**					
MDS	0.681 (0.675-0.688)	0.686 (0.68-0.693)	0.005	0.706 (0.700-0.713)	0.025
rMED	0.681 (0.675-0.688)	0.688 (0.681-0.694)	0.007	0.707 (0.700-0.713)	0.026
MSDPS	0.681 (0.675-0.688)	0.685 (0.678-0.692)	0.004	0.706 (0.699-0.712)	0.025
DQI	0.681 (0.675-0.688)	0.689 (0.682-0.696)	0.008	0.706 (0.700-0.713)	0.025
HNFI	0.681 (0.675-0.688)	0.685 (0.679-0.692)	0.004	0.706 (0.699-0.712)	0.025
HEI-2010	0.681 (0.675-0.688)	0.687 (0.68-0.694)	0.006	0.706 (0.700-0.713)	0.025
WHOHDI	0.681 (0.675-0.688)	0.683 (0.676-0.69)	0.002	0.705 (0.698-0.711)	0.024
DASH	0.681 (0.675-0.688)	0.686 (0.679-0.693)	0.005	0.706 (0.699-0.712)	0.025
HLI - diet	0.681 (0.675-0.688)	0.686 (0.679-0.693)	0.005	0.706 (0.699-0.712)	0.025
HLI - total [d]	0.679 (0.671-0.686)	0.692 (0.684-0.699)	0.013	0.693 (0.686-0.700)	0.014
WCRF [e]	0.678 (0.67-0.685)	0.684 (0.676-0.691)	0.006	0.705 (0.698-0.712)	0.027

Abbreviations: MDS, Mediterranean Diet Scale; rMED, relative Mediterranean diet score; MSDPS, Mediterranean Style Dietary Pattern Score; DQI-I, Diet Quality Index – International; HNFI, Healthy Nordic Food Index; HEI-2010, Healthy Eating Index 2010; WHO HDI, World Health Organization Healthy Diet Index; DASH, Dietary Approach to Stop Hypertension; HLI, Healthy Lifestyle Index; HLI-diet, diet component of the HLI; WCRF, World Cancer Research Fund / American Institute for Cancer Research

[a] Baseline model includes only age as a predictor, stratified by sex and center;

[b] Model 1 = baseline + dietary score;

[c] Model 2= Model 1+ lifestyle factors: smoking, BMI, physical activity, educational level unless otherwise stated

[d] Model 2 = Model 1 + educational level because BMI, physical activity, smoking are components of the Healthy Lifestyle Index, n=376,553

[e] Model 2 = Model 1 + smoking and educational level as BMI and physical activity are components of the WCRF score, n=363,207
