# Supplementary material for: Diet Quality Scores and Prediction of All-Cause, Cardiovascular and Cancer Mortality in a Pan-European Cohort Study
Source: PLoS One. 2016 Jul 13;11(7):e0159025. doi: 10.1371/journal.pone.0159025 (PMC4943719; doi:10.1371/journal.pone.0159025)
Supplement: S5 Table — (PDF) [file pone.0159025.s010.pdf]

S5 Table. C statistic of the dietary scores alone for 10-year risk of all-cause, CVD and cancer mortality in 451,256 participants to the EPIC study

Score	C-statistic (95% CI)
**All-cause**	
MDS	0.55 (0.545 - 0.555)
rMED	0.556 (0.551 - 0.561)
MSDPS	0.547 (0.542 - 0.552)
DQI	0.549 (0.543 - 0.554)
HNFI	0.511 (0.506 - 0.516)
HEI-2010	0.54 (0.535 - 0.546)
WHO HDI	0.523 (0.518 - 0.528)
DASH	0.541 (0.536 - 0.546)
HLI diet	0.534 (0.529 - 0.539)
HLI total	0.568 (0.563 - 0.574)
WCRF	0.587 (0.581 - 0.592)
**CVD**	
MDS	0.559 (0.548 - 0.569)
rMED	0.563 (0.553 - 0.574)
MSDPS	0.551 (0.54 - 0.561)
DQI	0.553 (0.542 - 0.563)
HNFI	0.506 (0.496 - 0.516)
HEI-2010	0.543 (0.532 - 0.554)
WHO HDI	0.536 (0.526 - 0.546)
DASH	0.551 (0.54 - 0.561)
HLI diet	0.543 (0.533 - 0.554)
HLI total	0.586 (0.575 - 0.597)
WCRF	0.612 (0.602 - 0.623)
**Cancer**	
MDS	0.539 (0.532 - 0.547)
rMED	0.542 (0.534 - 0.549)
MSDPS	0.535 (0.527 - 0.542)
DQI	0.535 (0.528 - 0.543)
HNFI	0.509 (0.502 - 0.517)
HEI-2010	0.529 (0.522 - 0.537)
WHO HDI	0.519 (0.512 - 0.527)
DASH	0.532 (0.525 - 0.54)
HLI diet	0.526 (0.519 - 0.533)
HLI total	0.562 (0.554 - 0.569)
WCRF	0.563 (0.555 - 0.571)
