# Supplementary material for: Diet Quality Scores and Prediction of All-Cause, Cardiovascular and Cancer Mortality in a Pan-European Cohort Study
Source: PLoS One. 2016 Jul 13;11(7):e0159025. doi: 10.1371/journal.pone.0159025 (PMC4943719; doi:10.1371/journal.pone.0159025)
Supplement: S6 Table — (PDF) [file pone.0159025.s011.pdf]

S6 Table. Geographical differences in dietary scores across EPIC centres

			MDS		rMED		MSDPS		HLI diet		HNFI		WHO HDI		HEI 2010		DQI-I		DASH		WCRF	
		N	Mean	SD	Mean	SD	Mean	SD	Mean	SD	Mean	SD	Mean	SD	Mean	SD	Mean	SD	Mean	SD	Mean	SD
Norway	South-East	19021	3.7	1.3	8.1	2.4	24.0	6.5	31.6	7.9	3.0	1.3	3.2	0.8	57.9	7.0	56.9	7.7	18.8	3.1	.	.
	North-West	15488	3.6	1.2	7.8	2.2	22.0	6.1	31.5	7.7	3.0	1.3	3.1	0.8	57.4	6.9	56.8	7.5	18.5	3.0	.	.
France	Ile-de-France	12436	4.4	1.5	8.9	2.7	25.3	6.8	31.6	8.0	3.0	1.3	3.3	0.8	63.2	7.0	58.4	6.7	22.9	3.2	2.4	1.0
	North-West	11527	4.4	1.4	9.1	2.7	24.6	6.6	31.5	7.8	3.0	1.3	3.3	0.8	63.1	6.6	58.4	6.5	23.1	3.2	2.3	1.0
	North-East	14318	4.1	1.5	8.5	2.7	23.6	6.5	31.7	7.9	3.0	1.3	3.3	0.8	62.5	6.9	57.5	6.7	22.7	3.2	2.3	1.0
	Rhone-Alpes	8850	4.4	1.4	9.3	2.6	24.7	6.6	31.6	7.8	3.0	1.3	3.5	0.8	63.3	6.4	59.5	6.5	23.6	3.1	2.5	1.0
	Provence	8865	4.6	1.5	9.3	2.6	25.2	6.9	31.4	7.8	3.0	1.3	3.5	0.8	63.5	6.9	59.2	6.7	23.5	3.1	2.5	1.0
	South-West	9609	4.6	1.5	9.1	2.6	24.9	6.7	31.4	7.8	3.0	1.3	3.4	0.8	63.5	6.6	58.9	6.6	23.5	3.2	2.5	1.0
Italy	Florence	12218	5.1	1.3	10.8	2.3	28.4	6.8	31.5	7.8	2.6	1.3	3.2	0.8	58.4	7.6	58.1	6.9	21.6	3.3	2.7	1.0
	Varese	10756	4.6	1.4	10.1	2.3	26.3	6.8	31.5	7.8	3.0	1.2	3.1	0.8	59.0	7.7	55.0	6.6	20.7	3.4	2.5	1.0
	Ragusa	5729	5.1	1.1	10.9	2.0	25.4	6.1	31.5	7.3	2.6	1.2	3.7	0.8	55.5	6.7	61.1	7.2	20.6	3.1	2.6	1.0
	Turin	9384	5.0	1.3	11.2	2.4	28.5	6.9	31.6	7.6	2.8	1.3	3.2	0.8	58.9	8.0	57.4	6.7	21.2	3.4	2.8	1.0
	Naples	4720	6.6	1.0	13.1	1.9	31.2	6.2	31.4	7.2	2.5	1.4	3.7	0.8	63.5	5.7	61.7	6.2	25.7	2.8	2.2	0.9
Spain	Asturias	8023	5.0	1.3	10.3	2.4	28.9	8.0	31.8	7.7	3.0	1.1	3.2	0.9	65.5	7.7	56.4	8.6	23.3	3.4	2.3	1.0
	Granada	6785	5.5	1.3	11.5	2.3	31.8	8.0	31.7	7.9	4.0	0.7	3.5	0.8	63.9	7.0	58.7	7.5	24.6	3.2	2.6	0.9
	Murcia	7411	5.9	1.2	11.9	2.4	28.7	7.2	31.5	7.6	3.5	0.9	3.8	0.8	64.7	6.8	60.9	6.9	25.0	3.1	2.7	1.0
	Navarra	7484	5.4	1.2	10.7	2.5	29.0	7.7	31.6	7.6	3.5	0.9	3.4	0.8	65.1	7.8	57.4	7.3	23.6	3.2	2.4	1.0
	San Sebastian	7627	5.6	1.2	11.3	2.5	30.0	8.0	31.3	7.3	3.2	1.0	3.4	0.9	64.3	7.8	59.6	8.1	24.1	3.4	2.5	1.0
UK	Cambridge	20514	4.1	1.5	8.3	2.6	21.5	6.7	31.8	8.3	2.6	1.3	3.4	0.9	60.6	8.8	58.9	8.5	21.7	3.7	2.6	1.0
	Oxford health conscious	43163	5.0	1.4	10.8	2.4	23.6	6.5	31.4	7.4	2.6	1.3	4.0	0.9	61.0	8.0	61.3	8.2	24.0	3.7	3.1	1.1
	Oxford general population	6318	4.4	1.5	9.3	2.5	24.0	6.2	31.7	8.3	2.6	1.4	3.4	0.9	60.8	7.9	59.6	8.2	21.7	3.7	2.4	1.0
Holland	Bilthoven	20210	2.6	1.3	6.2	2.5	17.7	5.7	31.5	7.7	2.6	1.3	3.4	0.8	54.5	8.9	55.2	7.3	18.7	3.2	2.7	1.0
	Utrecht	14631	2.4	1.2	6.0	2.4	18.5	5.7	31.5	7.8	2.6	1.2	3.6	0.8	60.6	7.4	58.0	6.7	20.3	3.2	2.6	1.0
Greece	Greece	22965	6.0	1.0	12.6	1.9	31.0	6.8	31.5	6.9	3.0	1.2	4.1	0.5	64.9	6.4	62.7	4.8	25.2	2.7	3.1	1.0
Germany	Heidelberg	22468	2.8	1.3	7.8	2.6	19.3	6.1	31.4	7.5	2.5	1.3	2.9	0.7	50.7	9.0	53.4	7.9	18.7	3.2	2.3	1.0
	Potsdam	23045	2.7	1.3	7.1	2.4	18.6	5.6	31.5	7.1	2.5	1.3	3.0	0.8	51.2	8.2	56.1	6.9	18.3	2.9	2.4	1.0
Sweden	Malmo	22705	2.9	1.4	6.2	2.5	16.9	5.6	31.5	8.2	2.7	1.2	2.9	0.8	57.0	8.3	54.7	7.8	18.4	3.5	2.4	1.0
	Umea	23796	2.6	1.3	5.3	2.1	11.5	4.5	31.4	8.0	2.9	1.4	3.3	0.7	49.1	8.4	52.6	9.5	17.8	3.2		
Denmark	Aarhus	15508	3.0	1.4	6.7	2.6	20.2	6.4	31.5	8.3	2.5	1.4	3.0	0.9	54.1	8.8	56.8	7.3	17.9	3.5	2.6	1.0
	Copenhagen	35682	3.2	1.4	7.1	2.8	21.1	6.8	31.5	8.4	2.5	1.4	3.1	0.9	53.3	9.7	57.0	7.9	18.3	3.6	2.5	1.0

Abbreviations: MDS, Mediterranean Diet Scale; rMED, relative Mediterranean diet score; MSDPS, Mediterranean Style Dietary Pattern Score; DQI-I, Diet Quality Index – International; HNFI, Healthy Nordic Food Index; HEI-2010, Healthy Eating Index 2010; WHO HDI, World Health Organization Healthy Diet Index; DASH, Dietary Approach to Stop Hypertension; HLI, Healthy Lifestyle Index; HLI-diet, diet component of the HLI; WCRF, World Cancer Research Fund / American Institute for Cancer Research
