# Supplementary material for: Diet Quality Scores and Prediction of All-Cause, Cardiovascular and Cancer Mortality in a Pan-European Cohort Study
Source: PLoS One. 2016 Jul 13;11(7):e0159025. doi: 10.1371/journal.pone.0159025 (PMC4943719; doi:10.1371/journal.pone.0159025)
Supplement: S7 Table — a Model including the following predictors: age at baseline, BMI (continuous), Physical activity (Cambridge index), smoking status (3 categories) and educational level, unless otherwise stated. Stratified by study center. b Model including the following predictors: age at baseline, BMI (continuous), Physical activity (Cambridge index), smoking status (3 categories) and educational level, unless otherwise stated. Stratified by sex and study center. c HR for the increase of 1 SD of score. d Model only including HLI, age and educational level because BMI, physical activity, smoking are components of the Healthy Lifestyle Index, n = 376,553. e Model only including WCRF score, smoking and educational level as BMI and physical activity are components of the WCRF score, n = 363,207. (PDF) [file pone.0159025.s012.pdf]

S7 Table. Age and sex specific multivariate hazard ratios (Model 2: adjusted for age and lifestyle risk factors) for 10-year mortality risk for a 1SD increase of score among 451,256 participants of the EPIC study

	Sex [a]		Age [b]	
	**Men**	**Women**	**<50y**	**>=50y**
**All-cause mortality**	HR (95% CI) [c]	HR (95% CI) [c]	HR (95% CI) [c]	HR (95% CI) [c]
MDS	0.91 (0.89 - 0.93)	0.92 (0.90 - 0.94)	0.92 (0.88 - 0.96)	0.91 (0.90 - 0.93)
rMED	0.87 (0.85 - 0.90)	0.91 (0.89 - 0.93)	0.91 (0.87 - 0.95)	0.89 (0.88 - 0.91)
MSDPS	0.91 (0.88 - 0.93)	0.93 (0.90 - 0.95)	0.91 (0.88 - 0.95)	0.92 (0.90 - 0.93)
DQI-I	0.90 (0.88 - 0.93)	0.89 (0.87 - 0.91)	0.89 (0.86 - 0.93)	0.90 (0.88 - 0.91)
HNDS	0.93 (0.91 - 0.95)	0.93 (0.91 - 0.95)	0.93 (0.89 - 0.97)	0.93 (0.92 - 0.95)
HEI 2010	0.90 (0.88 - 0.92)	0.92 (0.90 - 0.94)	0.93 (0.89 - 0.97)	0.91 (0.90 - 0.93)
WHO HDI	0.95 (0.93 - 0.97)	0.96 (0.93 - 0.98)	0.96 (0.92 - 1.00)	0.95 (0.94 - 0.97)
DASH	0.91 (0.89 - 0.93)	0.92 (0.90 - 0.94)	0.91 (0.88 - 0.95)	0.92 (0.90 - 0.94)
HLI – diet	0.94 (0.92 - 0.96)	0.93 (0.91 - 0.95)	0.96 (0.92 - 1.00)	0.93 (0.91 - 0.95)
HLI - total [d]	0.77 (0.76 - 0.79)	0.80 (0.79 - 0.82)	0.82 (0.79 - 0.86)	0.78 (0.77 - 0.80)
WCRF [e]	0.86 (0.84 - 0.88)	0.86 (0.84 - 0.88)	0.90 (0.86 - 0.95)	0.86 (0.84 - 0.87)
**CVD mortality**				
MDS	0.91 (0.87 - 0.95)	0.91 (0.87 - 0.96)	0.91 (0.82 - 1.01)	0.91 (0.88 - 0.95)
rMED	0.89 (0.85 - 0.93)	0.89 (0.84 - 0.93)	0.86 (0.78 - 0.96)	0.89 (0.86 - 0.93)
MSDPS	0.94 (0.90 - 0.98)	0.89 (0.85 - 0.94)	0.86 (0.78 - 0.96)	0.93 (0.90 - 0.96)
DQI-I	0.92 (0.88 - 0.96)	0.86 (0.82 - 0.90)	0.89 (0.80 - 0.99)	0.90 (0.87 - 0.93)
HNDS	0.93 (0.89 - 0.97)	0.93 (0.89 - 0.98)	0.87 (0.78 - 0.96)	0.94 (0.91 - 0.97)
HEI 2010	0.93 (0.89 - 0.97)	0.92 (0.87 - 0.96)	0.86 (0.78 - 0.95)	0.93 (0.90 - 0.97)
WHO HDI	0.95 (0.91 - 0.99)	0.91 (0.86 - 0.95)	0.85 (0.77 - 0.95)	0.94 (0.91 - 0.97)
DASH	0.90 (0.86 - 0.94)	0.89 (0.85 - 0.94)	0.84 (0.76 - 0.94)	0.90 (0.87 - 0.94)
HLI – diet	0.91 (0.87 - 0.95)	0.91 (0.87 - 0.96)	0.88 (0.80 - 0.98)	0.91 (0.88 - 0.95)
HLI - total [d]	0.73 (0.7 - 0.77)	0.76 (0.72 - 0.80)	0.74 (0.66 - 0.83)	0.74 (0.72 - 0.77)
WCRF [e]	0.83 (0.80 - 0.87)	0.83 (0.79 - 0.88)	0.87 (0.77 - 0.98)	0.83 (0.80 - 0.86)
**Cancer**				
MDS	0.93 (0.90 - 0.96)	0.92 (0.90 - 0.95)	0.92 (0.86 - 0.98)	0.93 (0.90 - 0.95)
rMED	0.90 (0.87 - 0.94)	0.93 (0.90 - 0.96)	0.94 (0.88 - 1.00)	0.91 (0.89 - 0.94)
MSDPS	0.92 (0.89 - 0.95)	0.95 (0.92 - 0.98)	0.95 (0.89 - 1.01)	0.93 (0.91 - 0.96)
DQI-I	0.91 (0.88 - 0.94)	0.91 (0.88 - 0.94)	0.89 (0.84 - 0.95)	0.91 (0.89 - 0.93)
HNDS	0.93 (0.90 - 0.97)	0.94 (0.91 - 0.97)	0.95 (0.90 - 1.02)	0.93 (0.91 - 0.96)
HEI 2010	0.90 (0.87 - 0.93)	0.94 (0.91 - 0.97)	0.97 (0.92 - 1.03)	0.92 (0.89 - 0.94)
WHO HDI	0.95 (0.92 - 0.99)	0.97 (0.95 - 1.00)	0.98 (0.92 - 1.04)	0.96 (0.94 - 0.99)
DASH	0.93 (0.90 - 0.97)	0.93 (0.90 - 0.96)	0.92 (0.86 - 0.98)	0.93 (0.91 - 0.96)
HLI – diet	0.95 (0.91 - 0.98)	0.93 (0.90 - 0.96)	0.94 (0.88 - 0.99)	0.94 (0.91 - 0.96)
HLI - total [d]	0.77 (0.75 - 0.80)	0.82 (0.79 - 0.85)	0.84 (0.79 - 0.90)	0.80 (0.77 - 0.82)
WCRF [e]	0.90 (0.87 - 0.93)	0.90 (0.87 - 0.93)	0.97 (0.90 - 1.04)	0.89 (0.87 - 0.91)

Abbreviations: MDS, Mediterranean Diet Scale; rMED, relative Mediterranean diet score; MSDPS, Mediterranean Style Dietary Pattern Score; DQI-I, Diet Quality Index – International; HNFI, Healthy Nordic Food Index; HEI-2010, Healthy Eating Index 2010; WHO HDI, World Health Organization Healthy Diet Index; DASH, Dietary Approach to Stop Hypertension; HLI, Healthy Lifestyle Index; HLI-diet, diet component of the HLI; WCRF, World Cancer Research Fund / American Institute for Cancer Research

[a] Model including the following predictors: age at baseline, BMI (continuous), Physical activity (Cambridge index), smoking status (3 categories) and educational level, unless otherwise stated. Stratified by study center.
[b] Model including the following predictors: age at baseline, BMI (continuous), Physical activity (Cambridge index), smoking status (3 categories) and educational level, unless otherwise stated. Stratified by sex and study center.
[c] HR for the increase of 1 SD of score
[d] Model only including HLI, age and educational level because BMI, physical activity, smoking are components of the Healthy Lifestyle Index, n=376,553
[e] Model only including WCRF score, smoking and educational level as BMI and physical activity are components of the WCRF score, n=363,207
